# The measurement of competitiveness of forest green food industry in Yunnan Province

**DOI:** 10.1371/journal.pone.0261133

**Published:** 2021-12-10

**Authors:** Zhongming Li, Wei Fu, Thomas Bilaliib Udimal, Mingcan Luo, Jiancheng Chen

**Affiliations:** 1 School of Economics and Management, Southwest Forestry University, Kunming, Yunnan, China; 2 School of Economics and Management, Beijing Forestry University, Beijing, China; Szechenyi Istvan University: Szechenyi Istvan Egyetem, HUNGARY

## Abstract

Improvement in living standards has led to the development and utilization of forest green foods. The study seeks to examine the foundation and potential of forest green food industry in Yunnan Province. By constructing the industrial competitive advantage model, this paper measured and analyzed the competitiveness of forest green food industry in Yunnan Province from 2016 to 2020 by using fuzzy evaluation method and AHP. The conclusions were as follows: (1) The competitiveness of forest green food industry in Yunnan Province was at a medium level with competitiveness index of 83.98. (2) The competitive advantage of forest green food industry in Yunnan Province mainly depended on key factors such as natural endowment and education level. The area is however not having comparative advantage in general factors and important factors. Therefore, there is the need to put in place measures to realise the full potential of forest green food industry in the area by providing players in the sector with requisite skills.

## Introduction

Serious concerns expressed during the last two decades regarding the use of chemicals in agriculture in terms of their adverse impact on the human health, environment and sustainable agricultural production. These concerns have led to the worldwide promotion of organic food production [1]. The market for organic food products is growing rapidly worldwide, such foods meet certified organic standards for production, handling, processing, and marketing [2]. With increasing awareness on safety consumption, healthy lifestyle and environmental protection, the competition within green organic food industry is becoming increasingly fierce [3]. Developing the green industry is China’s national strategy to achieve a sustainable path and prevent further degradation of the environment [4]. In 2021, the No.1 Central Document has put forward some recommendations for the development of green agricultural products and organic agricultural products through quality certifications of edible agricultural products. As the most important milestone in the industry and green environmental protection industry in the 21st century, forest green food industry has gradually become the mainstream direction of today’s green industry, which provides support for sustainable development of China’s forestry and has become a new economic growth point. Yunnan Province, which contains abundant forest resources in China, has issued a number of relevant policies in recent years to promote the development of forest green food industry and to enhance competitiveness of forest green food industry in Yunnan Province.

Both in theoretical research and empirical research, it is shown that organic food industries in Europe, America and other countries had quite complete systems [5]. Although there were differences in the degree of development, market operation and market management of organic food industry in developed countries are characterized by standardized operation, strict management and perfect industrial system of organic food market [6]. The theoretical research on organic food was more in-depth and systematic, forming a relatively complete theoretical system in the aspects of organic food consumer behavior, market competition, policy support and so on. International studies have classified forest green food as organic food without independent analysis and certification. However, the research results of organic food theory had a good reference and guidance for the development of forest green food industry. The existing research has mainly carried out from four aspects: concept classification, consumption preference, policy support and industrial competitiveness evaluation.

In terms of concept classification, the research on forest food began in the early 20th century. King [7] published the book "four thousand years of farmers", which pointed out that human and animal manure and straw used in China’s agricultural planting are good organic fertilizers, and the fungal food cultivated with this fertilizer is the earliest organic food. Norikane [8] used "big business" to describe the industrial status of forest food industry in the forest. Forest food was also known as "safe food" in Liberia. Forest food was an emerging multi-functional perennial mixed food production system [9]. By solving the three main components of food security: supply, access and utilization, it is possible to promote food security and alleviate malnutrition in urban and suburban areas [10]. Only forest food that has passed social certification, organic certification, product quality certification and international forest certification has qualified forest food [11]. Forest food had an important contribution to the quality of human diet. According to the research, there were four use modes of forest food: forest food dependence, limited use of forest food, forest food supplement and professional consumers of forest food [12]. Green Food was officially defined to include food grown under the conditions of strict supervision, control and regulation in production, processing, packing, storage and transportation. Forest green foods were a subset of forest foods and refer to as uncultivated foods from forested areas [13]. Forest green food was mostly in wild states, rich in nutrition, delicious tastes, clean and pollution-free features which meet the growing demand for green and organic food. Green food adopted the whole-some quality control from field to table, while it required reasonable applications of inputs, including pesticide, fertilizer, veterinary drug and additive etc. to prevent any pollution of toxic and harmful matters in various stages of food processing so as to ensure environmental and product safety [14]. Forest green food industry referred to as the industry that cultivates, processes, circulates, sells and provides related services for forest green food based on sustainable utilization of forest resources and good ecological environments, which included multiple categories of primary industry, secondary industry and tertiary industry of national economy [15].

In terms of consumption preference, consumers of organic food were the guide of consumption opinions and should pay attention to the oral effect among consumers. Most consumers believed that organic food has health and environmental benefits, but lack of information, inappropriate marketing practices and high price are some of barriers that hinder the consumption of organic food [16]. Different store business models and different types of families had different price sensitivity of organic food, which would form specific family purchase loyalty and affect each family’s willingness to pay for organic food [17]. Because consumers were more rational when consuming organic food, they are more sensitive to the price of organic food, and were willing to pay more for expensive organic food [18]. In fact, where there were only small visual and sensory differences between organic products and conventional products, consumers could only choose a product based on trust in the producers, so reputation was particularly important in the organic food system [19]. Some organic food consumers have preference for food quality and safety, and sometimes offer suggestions for improvement in supply chains to promote the sales of organic food [20].

From the policy support aspect in recent years, the sustainable development of forest food could drive the sustainable development of forest resources, which is essential in attainment of different sustainable development goals [21]. The introduction of No.1 Central Document has provided a strategic direction for the development of forest green food industry [22]. The development of forest green food industry is still at the primary stage, so there is was the need to seize opportunities at national macro policy level, and establish a forest green food development system with regional characteristics as soon as possible [23]. Nigeria has also put forward many relevant policies aimed at accelerating the sustainable development, management and environmental protection of forests, but the expected results have not been achieved [24]. In 2013, the national food security act of India marked a historic change in food security from a welfare based approach to a rights based approach. However, Bose’s [25] research showed that the public distribution system of the act was not suitable for ensuring the food security of indigenous people and did not promote the development of forest food industry.

In terms of industrial competitiveness evaluation, previous studies usually divided the development level and development potential of forestry industry into four types: "high level—high potential", "high level—low potential", "low level—high potential" and "low level—low potential" [26]. On this basis, the research results showed that the development potential of plantation industry was huge [27], and the production potential of forestry industry in most areas was greater than the sales potential [28]. Industrial development potential was an important index to evaluate the degree of industrial development. It could improve the development potential of forestry industry from three aspects: brand construction, trust maintenance and service [29]. Whether a domestic enterprise could give play to its competitive advantage was greatly affected by the domestic economic environment. Among them, production factors, demand, related industries and supporting industries, as well as its enterprise strategic combination and competition were the most influential and direct factors, in which opportunity and the government played an auxiliary role. With the continuous improvement of product quality standards, high-tech organic food had more advantages in market competition [30]. Song [31] analyzed the actual situation by calculating the index parameters in the diamond industry analysis system, and concluded Heilongjiang has comparative advantage in green organic food industry. Wang et al. [32] established a competitive advantage evaluation model based on diamond system theory to evaluate the competitiveness of forest green food industry in Heilongjiang Province. Liu et al. [33] analyzed the international competitiveness of China’s nut forest food by using international market share rate, trade competition index and revealed symmetric comparative advantage index. Raynolds [34] believed that the competitive advantage of the organic food industry could be achieved through product innovation.

At both domestic and international levels, scholars have made rich contributions on forest green food industry, but there is the need to further explore the topic from territorial perspectives. At present, domestic and foreign scholars have studied the competitiveness of forest green food industry from the national perspective, and studies based on scale of Yunnan Province are still needed. In view of this, the study looks at the connotation and characteristics of forest green food industry constructing competitiveness evaluation index system of forest green food industry from four aspects: production factors, demand conditions, enterprise strategy, structure and horizontal competition, and related and supporting industries, and made empirical analysis by using fuzzy evaluation method and AHP. The study focuses mainly on the competitiveness of forest green food industry in Yunnan Province by analyzing the competitiveness index of key factor level, general factor level and important factor level criteria factors.

## Methods

Firstly, the evaluation index system of forest green food industry competitiveness is first constructed. Fuzzy evaluation method [35] and AHP [36] are used for the empirical analysis of the competitiveness of forest green food industry in Yunnan Province. Fuzzy evaluation method has the characteristics of clear results and strong systematicness. It can better solve fuzzy and difficult to quantify problems, and is suitable for solving various uncertain problems. AHP can effectively analyze the non sequential relationship between the levels of the objective index system, and effectively measure the judgment and comparison of decision makers. Therefore, these two methods are selected to evaluate the competitiveness of forest green food industry in Yunnan Province clearly and objectively, and using the two methods at the same time can enhance the authenticity and reliability of the evaluation results.

### Study area

The study is carried out in Yunnan province, China. Yunnan is located at 21°8’ - 29°15’ N and 97°31’ - 106°11’ E, and borders Guizhou province, Sichuan province, and Guangxi province. With a total area of 394,100 km^2^, it contains 16 prefecture-level administrative divisions [37]. The current resident population of Yunnan Province is 48.583 million.

Yunnan is a low latitude region with high terrain in the northwest and low terrain in the southeast. It falls step by step from north to south. It is a mountainous plateau terrain, and the mountainous area accounts for 88.64% of the total area of the province. With gorgeous natural scenery, it is one of the important birthplaces of human civilization. Yunnan Province is extremely rich in forest food resources, with many kinds and wide distribution. According to the records of Yunnan statistical yearbook, in 2020, the forest area of the whole province reached 23.9265 million hm^2^, the forest coverage rate of the whole province reached 62.4%, and the forestry output value reached 39.554 billion yuan.

### Data sources

The competitive advantage evaluation system of forest green food industry includes four primary indicators: production factors, demand conditions, enterprise strategy, structure and horizontal competition, and related and supporting industries, and sixteen secondary indicators such as total area, investment amount and number of employees. The data of secondary indicators directly affect the measurement of the competitiveness of forest green food industry in Yunnan Province. The data used for the analysis span from 2016 to 2020.

Secondary indicators include "total value", "qualitative value", "ratio value" and "quantitative value". Where, "total value" is the sum of annual data of the indicator; "qualitative value" refers to the score given by experts; "ratio value" is the ratio of indicators; "quantitative value" is the value of the indicator. "total value" indicators such as total area and investment amount and "quantitative value" indicators such as technical status are directly obtained from Yunnan Statistical Yearbook; natural endowment, food safety and other "qualitative value" indicators are obtained from the investigation of relevant experts; "ratio value" indicators such as per capital disposable income and market share are sorted out according to Yunnan statistical yearbook.

### Forest green food industry competitiveness model

The index system of forest green food industry competitiveness evaluation is constructed by considering the indexes used in forest green food industry competitiveness and other related industries competitiveness evaluations which are frequently used. Finally, four indexes are selected which include production factors, demand conditions, enterprise strategy, structure and horizontal competition, and related and supporting industries.

The comprehensive evaluation of competitive advantage is divided into general factor layer and specific factor layer. The general factor layer, namely the main criterion layer B, which is referred to as the first level index; the specific factor level which is referred to as sub criteria layer C, is also known as secondary indicators. There are two ways to determine the score of specific factor level. First, if the relevant statistical index value can be obtained directly, the score can be determined by referring to the corresponding score interval. Second, if it is difficult to obtain data through relevant indicators, experts will score them. Based on this the latter, the index system structure model of forest green food industry competitive advantage (Table 1) and identification index (Table 2) are constructed.

**Table 1:** Index system structure model of competitive advantage.

Target layer A	Main criteria layer B	Sub criteria layer C
Comprehensive evaluation system of industrial competitive advantage X	Factors of production X_1_	total area W_11_
		investment amount W_12_
		natural endowment W_13_
		number of employees W_14_
	Demand conditions X_2_	market demand W_21_
		food safety W_22_
		education level W_23_
		per capita disposable income W_24_
	Enterprise strategy, structure and competition in the same industry X_3_	market share W_31_
		product export rate W_32_
		total assets W_33_
		industrial environment W_34_
	Related and supporting industries X_4_	credit environment W_41_
		social environment W_42_
		technical status W_43_
		Intermediary organization W_44_

**Table 2:** Identification index, meaning and test method of competitive advantage of forest green food.

Serial number	Index name	category	meaning	test method
**1**	total area	total value	total planting area of forest green food	planting area
**2**	investment amount	total value	total investment amount of the industry	total investment
**3**	natural endowment	qualitative value	the superiority and monopolization of natural endowment	questionnaire survey (good, average, poor)
**4**	number of employees	total value	total number of employees in forest green food enterprises	total number of employees
**5**	market demand	total value	annual sales revenue	annual sales
**6**	food safety	qualitative value	people’s attention to food safety	questionnaire survey (high, average, low)
**7**	education level	qualitative value	popularization of green knowledge and environmental protection education	questionnaire survey (high, average, low)
**8**	per capita disposable income	ratio value	per capita disposable income of urban population	per capita disposable income
**9**	market share	ratio value	share of forest green agricultural products in agricultural products	output value of forest green agricultural products / output value of agricultural products × 100%
**10**	product export rate	ratio value	Proportion of export sales in total sales	exports / sales × 100%
**11**	total assets	total value	total assets of forest green food enterprises at the end of the year	total assets at the end of the year
**12**	industrial environment	qualitative value	the average profit level of the industry in which the enterprise is located	questionnaire survey (good, average, poor)
**13**	credit environment	qualitative value	can the payment for goods be recovered in time, and is the default between enterprises serious	questionnaire survey (good, average, poor)
**14**	social environment	qualitative value	unreasonable burden distribution, local government departments provide services to practitioners	questionnaire survey (good, average, poor)
**15**	technical status	quantitative value	number of technicians	number of technicians
**16**	intermediary organization	qualitative value	whether the intermediary organizations are well-informed has a great impact on the promotion of industrial development	questionnaire survey (good, average, poor)

Firstly, membership function is established. We use X = (*X*_1_, *X*_2_, *X*_3_, *X*_4_) to describe the competitive advantage of forest green food industry, and evaluate the industry’s competitive advantage in each factor. Suppose the evaluation set is Y = {*Y*_1_, *Y*_2_, *Y*_3_}, where Y is the fuzzy comprehensive evaluation of the industry’s competitive advantage from the perspective of secondary index X_*ij*_. The membership functions of the three grades are as follows:

$$U_{Y_{1}}\;(\delta )=\left\{ \begin{array}{c} \frac{1}{15}\;(\delta -85),85\leq \delta \leq 100\\ 0,\delta <85 \end{array}\right.$$ (1)

$$U_{Y_{2}}\;(\delta )=\left\{ \begin{array}{c} -\frac{1}{15}\;(\delta -85),70\leq \delta \leq 85\\ \frac{1}{10}\;(\delta -60),\delta <70 \end{array}\right.$$ (2)

$$U_{Y_{3}}\;(\delta )=\left\{ \begin{array}{c} -\frac{1}{10}\;(\delta -70),60\leq \delta \leq 70\\ 1,\delta <60 \end{array}\right.$$ (3)

Secondly, the fuzzy evaluation matrix is established. The main criteria layer fuzzy evaluation matrix is established to determine the fuzzy evaluation results of the evaluation object:

$$\bar{A}=(a_{1},a_{2},a_{3})=B\times \left[\begin{array}{c} A_{1}\\ \begin{array}{c} A_{2}\\ A_{3} \end{array}\\ A_{4} \end{array}\right],\;a_{j}={⋁}_{i=1}^{3}(B_{i}{⋀}_{i=1}^{4}a_{ij})$$ (4)

Normalize $\bar{A}$ and use fuzzy distribution method to evaluate industrial competitive advantage, and the final score of fuzzy evaluation of industrial competitive advantage is obtained.

After analyzing the industrial competitive advantage, its strength is further assessed. On the one hand, it can objectively evaluate the competitive advantage of the industry. On the other hand, with more important observations, it can assess both constraints and successes of the industry and improve the competitive advantage through the evaluation of the intensity. Through the attributes of each evaluation index, it can appropriately adjust the business activities to further optimize and improve the competitive advantage of the industry.

### Empirical analysis of Yunnan

Taking Yunnan Province as a sample, the reasons for testing and analyzing the competitive advantage model of forest green food industry are: firstly, the forest green food industry in Yunnan Province is developed earlier and the data is relatively abundant; secondly, the forest green food industry in Yunnan Province has developed rapidly and has representative characteristics of the whole country.

### Descriptive statistics

The quantitative data classified as "total value" "ratio value" and "quantitative value" in the index system are from the statistical yearbook of Yunnan Province as shown in (Table 3).

**Table 3:** Data of forest green food industry in Yunnan Province from 2016 to 2020.

Category	Company	2016	2017	2018	2019	2020
**total area**	ten thousand hectares	714.94	714.82	716.46	689.08	695.95
**investment amount**	USD 100 mn	0.28	0.26	0.23	0.32	0.14
**number of employees**	ten thousand people	5.48	5.03	4.19	3.55	2.89
**market demand**	RMB 100 mn	6390.83	7222.73	8194.79	9197.26	10158.23
**per capita disposable income**	ten thousand yuan	5.50	6.36	7.35	8.05	9.18
**market share**	%	14.71	14.53	16.14	15.08	12.86
**product export rate**	%	16.91	10.42	9.07	9.05	9.63
**total assets**	RMB 100 mn	151.45	158.96	204.48	210.28	204.68
**technical status**	ten thousand people	0.03	0.02	0.02	0.01	0.32

The data are from Yunnan Statistical Yearbook (2016–2020).

The weight of each index of the impact assessment object and the evaluation value of "qualitative value" in the index system are all obtained through questionnaire administering involving forest green food experts in Yunnan Province. Through the questionnaire survey of experts, the relevant evaluation values are obtained, and the survey results sorted out by taking the average value (Table 4).

**Table 4:** Average results of expert investigation.

influence factor	W_11_	W_12_	W_13_	W_14_	W_21_	W_22_	W_23_	W_24_	W_31_	W_32_	W_33_	W_34_	W_41_	W_42_	W_43_	W_44_
**Weight(%)**	30	20	40	10	10	20	40	30	30	40	10	20	10	50	30	10
**Evaluation value**	-	-	good	-	-	average	average	-	-	-	-	average	poor	good	-	average

Using the formulas (1)—(3) to analyze the membership of the secondary factors.

Then the fuzzy evaluation matrix R_i_ is:

$$R_{1}=[\begin{array}{ccc} 0 & 0.33 & 0\\ 0 & 0.67 & 0\\ 0.33 & 0 & 0\\ 0 & 0.67 & 0 \end{array}]\;R_{2}=[\begin{array}{ccc} 0.33 & 0 & 0\\ 0 & 0.67 & 0\\ 0 & 0.33 & 0\\ 0.33 & 0 & 0 \end{array}]$$

$$R_{3}=[\begin{array}{ccc} 0 & 0.67 & 0\\ 0 & 0.67 & 0\\ 0 & 0.33 & 0\\ 0 & 0.33 & 0 \end{array}]\;R_{4}=[\begin{array}{ccc} 0 & 0 & 0.5\\ 0.33 & 0 & 0\\ 0.33 & 0 & 0\\ 0 & 0.33 & 0 \end{array}]$$

By *A*_*i*_ = *W*_*ij*_×*R*_*i*_ can be calculated as follows:

A_1_ = (0.13,0.30,0); A_2_ = (0.13,0.27,0); A_3_ = (0,0.57,0); A_4_ = (0.26,0.03,0.05)

Then, the fuzzy relation matrix $\bar{A}$ of evaluation target is constructed by using formula (4):

$$\bar{A}=(0.4,0.3,0.1,0.2)\times [\begin{array}{ccc} 0.13 & 0.30 & 0\\ 0.13 & 0.27 & 0\\ 0 & 0.57 & 0\\ 0.26 & 0.03 & 0.05 \end{array}]=(0.15,0.26,0.01)$$

And normalize $\bar{A}$,

$${\bar{A}}^{\prime }=\left[\begin{array}{ccc} \frac{0.15}{0.15+0.26+0.01} & \frac{0.26}{0.15+0.26+0.01} & \frac{0.01}{0.15+0.26+0.01} \end{array}\right]=[0.35\;0.63\;0.02]$$

Based on this result, there is a 35% certainty that Yunnan forest green food industry has strong competitive advantage, 63% certainty that its competitive advantage is average, and 2% certainty that its competitive advantage is weak. According to the principle of maximum membership degree, it can be considered that the competitive advantage of forest green food industry in Yunnan Province is at the general level.

The determination of evaluation index weight directly reflects the role of indicators in the development of enterprises, and directly affects the evaluation results under certain conditions. Therefore, the determination of index weight is the key to scientific identification and evaluation of competitive advantage. AHP is used to determine the weight of each index. The judgment matrix is constructed based on expert opinions, and the sum product method is used to solve the comprehensive judgment matrix. The weight of each index is determined by combining expert experience and mathematical model (Tables 5 and 6). Each matrix passed the consistency test of CR<0.1 fulfilling the condition for calculation of the weight of each index to the target layer.

**Table 5:** Influence weight of main criterion layer on target layer.

Criterion	Factors of production	Demand conditions	Enterprise strategy, structure and competition in the same industry	Related and supporting industries
**competitive edge**	0.558	0.263	0.057	0.122

**Table 6:** The final composite weight of sub criteria layer to target layer.

W _11_	W _12_	W _13_	W _14_	W _21_	W _22_	W _23_	W _24_	W _31_	W _32_	W _33_	W _34_	W _41_	W _42_	W _43_	W _44_
0.146	0.067	0.310	0.031	0.015	0.032	0.154	0.069	0.015	0.031	0.003	0.007	0.008	0.076	0.029	0.008

The index that can specifically measure the competitiveness of forest green food industry in Yunnan Province is been calculated. A study by Wang et al. [38] measured the competitiveness of forest green food industry in Yunnan Province and obtained 85–100, 70–85 and 60–70 for high level, medium level and low level, respectively (Table 7).

**Table 7:** Evaluation standard of forest green food industry competitiveness in Yunnan Province.

Competitiveness index of forest green food industry in Yunnan Province	Competitiveness level of forest green food industry in Yunnan Province
85–100	higher
70–85	secondary
60–70	low grade

### Result analysis

The influence degree of sub criteria factors on industrial competitive advantage is measured by the weight value, which is divided into three categories: key factors (>0.1), important factors (0.03–0.1) and general factors (<0.03) (Table 8). By calculating the product of the influence weight of each factor on the target and the dimensionless value of each factor, the competitiveness index of each factor is obtained, which is the competitiveness of the final evaluation target (Table 9).

**Table 8:** Comparison of influencing factors of forest green food industry in Yunnan Province.

Category	Influencing factors and degree
**critical factor**	natural endowment(0.310)> education level(0.154)> total area(0.146)
**important factor**	social environment(0.076)>per capita disposable income(0.068)>investment amount(0.067)>food safety(0.032)>number of employees(0.031) = product export rate(0.031)> technical status(0.029)
**general factor**	market demand(0.015) = market share(0.015)>credit environment(0.008) = intermediary organization(0.008)> industrial environment(0.007)> total assets(0.003)

**Table 9:** Competitiveness index of forest green food industry in Yunnan Province.

Main criteria layer factors	Sub criteria layer factors	Weight	Fraction	Competitiveness index
**Factors of production (X**_**1**_)	total area (W_11_)	0.146	80.0	11.68
	investment amount (W_12_)	0.067	75.0	5.03
	natural endowment (W_13_)	0.310	90.0	27.90
	number of employees (W_14_)	0.031	75.0	2.33
	subtotal	0.554		46.93
**Demand conditions (X**_**2**_)	market demand (W_21_)	0.015	90.0	1.35
	food safety (W_22_)	0.032	75.0	2.40
	education level (W_23_)	0.154	80.0	12.32
	per capita disposable income (W_24_)	0.069	90.0	6.12
	subtotal	0.269		22.19
**Enterprise strategy, structure and competition in the same industry (X**_**3**_)	market share (W_31_)	0.015	75.0	1.13
	product export rate (W_32_)	0.031	75.0	2.33
	total assets (W_33_)	0.003	80.0	0.24
	industrial environment (W_34_)	0.007	80.0	0.56
	subtotal	0.056		4.25
**Related and supporting industries (X**_**4**_)	credit environment (W_41_)	0.008	65.0	0.52
	social environment (W_42_)	0.076	90.0	6.84
	technical status (W_43_)	0.029	90.0	2.61
	intermediary organization (W_44_)	0.008	80.0	0.64
	subtotal	0.121		10.61
**total**				83.98

The result of AHP shows that the comprehensive competitiveness index of forest green food industry in Yunnan Province is 83.98, which is at the middle level. It is similar to the evaluation result under the principle of maximum membership degree of fuzzy comprehensive evaluation, and the conclusion that 63% membership degree is used to judge the competitive advantage of forest green food industry in Yunnan Province is same as the result of AHP, all of them are in the medium level.

## Conclusion and suggestion

Taking Yunnan Province as the study area, the evaluation index system of forest green food industry competitiveness based on four dimensions which include production factors, demand conditions, enterprise strategy, structure and horizontal competition, and related and supporting industries, and makes recommendations for policy consideration.

### Conclusion

The above empirical analysis of the fuzzy evaluation model and the AHP model for the competitive advantage of the forest green food industry in Yunnan Province showed that the industrial competitive advantage model based on the competitive advantage theory has strong pertinence and applicability in analyzing the competitive advantage of the growing forest green food industry. The model evaluation results showed that it is fully in line with the competitive advantage level of forest green food industry in Yunnan Province. After more than 20 years of efforts, the forest green food industry in Yunnan Province developed rapidly and was entering a new period of sustained and accelerated development. However, from the low export rate of forest green food in Yunnan Province, the competitive advantage of forest green food industry in Yunnan Province mainly came from its good ecological environment, large monitoring area and planting area, and extensive management, and these factors would no longer have a significant competitive advantage with the development of other regions, so it was difficult to sustain.

At present, the overall competitiveness index of forest green food industry in Yunnan Province was 83.98, which indication that it has a strong competitive advantage in the country; among the influencing factors, the competitiveness indexes of natural endowment, total area and education level of the key factors were 27.90, 11.68 and 12.32, respectively, an indication of comparative advantage, while the competitiveness indexes of per capita disposable income, investment amount of the important factors and industrial environment of the general factors were 6.12, 5.03 and 0.56, respectively, showing that green food industry has no comparative advantage in those areas.

Industry in Yunnan Province mainly depended on the critical factor, the sub criterion factors of general factors and important factors are at a disadvantage. There was no major inferior item in the sub criterion factors of production factor level. The sub criteria of the demand condition level, which was the second most important level, were not high as a whole, and individual projects had serious disadvantages. The sub criterion factors of the related supporting industries with the third importance level fluctuated greatly. Therefore, it is possible that the most important factor for Yunnan forest green food to gain competitive advantage is the competitiveness of key factors, but the influence of non-key factors could not be ignored. The next step to gain competitive advantages should start with the promotion of all important factors. Most of the important factors could be improved through deliberate policies, which was different from the key factors, which mainly relied on resource monopoly. Although general factors had an impact on competitive advantage, it was difficult to play a decisive role because of its low weight.

### Suggestion

Though the forest green food industry in Yunnan Province has a strong competitive advantage, the per capita disposable income, the amount of investment and the competitiveness index of industrial environment in general factors were areas that green food industry has no comparative advantage. Based on this, regulations should be forward for consideration to encourage investments in the sector.

#### Policy support

Firstly, there is the need to formulate and implement targeted industrial policies. The competitive advantage of forest green food industry in Yunnan Province mainly comes from the advantages of natural resources, such as variety, yield and location. It is not difficult to maintain these advantages at present; the difficulty is to transform the existing advantages of key factors into long-term advantages and eliminate the instability of short-term advantages. It is necessary to expand the competitive advantage of all the important factors and make these advantages play a positive role in promoting the positive development of relevant competitive factors. These factors mainly depend on the government, system, science and technology. Governments at all levels should adopt targeted industrial policies according to their geographical location and product characteristics, promote the development of industries and enterprises, and improve the comprehensive competitiveness of the industry.

Secondly, there is the need to strengthen the development of relevant supporting industries. The value of related industries and supporting industries lies not only in the ability to provide input for the leading industry with the lowest price, but also the geographical distance with the leading industry, which enables enterprises to transmit product information frequently and quickly, exchange innovation ideas, promote the technological upgrading of enterprises and create a benign and interactive "local economy".

#### Enterprise development

Firstly, using marketing strategy to stimulate demands. Because of the lack of general publicity of forest green food and the consumers’ lack of understanding of the value of forest green food, it is unable to form a stable concept of forest green food consumption, which limits the sectors’ growth. The public has a high awareness of the word "green food", but many people do not know about forest green food. Therefore, there is the need to meet the existing needs through objective and practical advertising and marketing strategies, and strengthen the guidance of consumers’ demands, so that the consumption awareness of forest green food is deeply rooted in the whole public.

Secondly, foster the concept of cooperation to promote win-win development. In the process of market internationalization, whether the products enter the international market and continue to expand the market coverage and share are important indicators to test the overall level of industry and competitiveness of enterprises. In this process, it is very important to cultivate the concept of cooperation and competition, and promote the development strategy of win-win scenarios. At present, the low market concentration, small enterprise scale, less well-known brand and weak market competitiveness of China forest green food industry are directly related to the cultivation and development of cooperative competition concept. In order to make China’s forest green food enter the international market, it is necessary to innovate. Through cooperation and competition, optimize product structure and develop domestic and international markets.

## Supporting information

S1 File The database of the Yunnan statistical yearbook (2016–2020). http://stats.yn.gov.cn/.
(Z01)

## References

[1] Dholakia J, Shukul M. Organic food: an assessment of knowledge of homemakers and influencing reasons to buy/not to buy. Journal of Human Ecology. 2012; 37(3): 221–227.

[2] Brantsaeter AL, Ydersbond TA, Hoppin JA, Haugen M, Meltzer HM. Organic food in the diet: exposure and health implications. Annual review of public health. 2017; 38: 295–313. doi: 10.1146/annurev-publhealth-031816-044437 27992727

[3] Yin S, Wu L, Du L, Chen M. Consumers’ purchase intention of organic food in China. Journal of the Science of Food and Agriculture. 2010; 90(8): 1361–1367. doi: 10.1002/jsfa.3936 20474056

[4] Sun C, Huang D, Li H, Chen C, Wang C, Li M, et al. Green food industry in China: Spatial pattern and production poncentration drivers. Frontiers in Environmental Science. 2021: 429. doi: 10.3389/fenvs.2021.665990

[5] Kaminska N, Gaworski M, Kazmierska P, Klepacka AM. Pilot study of variability on demand and knowledge concerning organic food on an example of two Polish regions. Agronomy Research. 2016; 14(1): 67–74.

[6] Kahl J. Organic food quality: from field to fork. 2012; 92(14): 2751–2752. doi: 10.1002/jsfa.5888 23077006

[7] King FH. Farmers of forty centuries or permanent agriculture in China Korea and Japan. Beijing: Oriental Publishing House; 2011.

[8] Norikane A. Big business in the forest: trading forest products to increase food sovereignty in Liberia. LEISA Magazine. 2009; 25 (3): 38–49.

[9] Wang JM, Nguyen N, Bu XZ. Exploring the roles of green food consumption and social trust in the relationship between perceived consumer effectiveness and psychological wellbeing. International Journal of Environmental Research and Public Health. 2020; 17(13): 4676. doi: 10.3390/ijerph17134676 32610596 PMC7369759

[10] Nytofte LSN, Henriksen CB. Sustainable food production in a temperate climate–a case study analysis of the nutritional yield in a peri-urban food forest. Urban Forestry & Urban Greening. 2019; 45: 126326. doi: 10.1016/j.ufug.2019.04.009

[11] Fungo R, Muyonga JH, Kabahenda M, Okia CA, Snook L. Factors influencing consumption of nutrient rich forest foods in rural Cameroon. Appetite. 2016; 97: 176–184. doi: 10.1016/j.appet.2015.12.005 26686583

[12] Rowland D, Ickowitz AMY, Powell B, Nasi R, Sunderland T. Forest foods and healthy diets: quantifying the contributions. Environmental Conservation. 2017; 44 (2): 102–114.

[13] Iqbal M. Trade restrictions affecting international trade in non-wood forest products. Food and Agriculture Organization of the United Nations, 1995.

[14] Yu X, Gao Z, Zeng Y. Willingness to pay for the “Green Food” in China. Food Policy. 2014; 45: 80–87.

[15] Zheng DS, Zhu ZF. Study on the development of green food industry chain in state-owned forest areas. Issues of Forestry Economics. 2019; 39(06): 621–627. (in Chinese)

[16] Shaheen MAR, El-Nakhlawy FS, Al-Shareef AR. Main factors influencing the spread and consumption of organic food in Saudi Arabia. Journal of Food Agriculture & Environment. 2013; 11(1): 231–233.

[17] Hsieh MF, Stiegert KW. Store format choice in organic food consumption. American Journal of Agricultural Economics. 2012; 94(2): 307–313.

[18] De YR. Encouraging environmentally appropriate behavior: the role of intrinsic motivation. Journal of Environmental Systems. 1985;l5(4): 281–292.

[19] Thorsoe MH. Maintaining trust and credibility in a continuously evolving organic food system. Journal of Agricultural & Environmental Ethics. 2015; 28(4): 767–787.

[20] Vietoris V, Kozelova D, Mellen M, Chrenekova M, Potclan JE, Fikselova M, et al. Analysis of consumer preferences at organic food purchase in Romania. Polish journal of food and nutrition sciences. 2016; 66(2): 139.

[21] Nischalke SM, Abebe M, Wondimagegnhu BA, Kriesemer SK, Beuchelt T. Forgotten forests? food potential of ancient coffee forests and agroforestry systems in the Southwestern Ethiopian mountains, seen through a gender lens. Mountain Research and Development. 2017; 37(3): 254–262.

[22] Zhai XJ, Ma GF. Identification and analysis of the development stage of forest food industry in Heilongjiang Province—based on the perspective of rural revitalization strategy. Forestry economy. 2019; 41(09): 80–86. (in Chinese)

[23] Wang YZ, Song Y. Strategic orientation of Heilongjiang forest food industry development based on “SWOT AHP” method. Issues of Forestry Economics. 2009; 29(05): 438–442. (in Chinese)

[24] Odebode SO. Contributions of selected non-timber forest products to household food security in Nigeria. Journal of Food Agriculture & Environment. 2005; 3(3–4): 138–141.

[25] Bose P. India’s Right To Food Act: human rights for tribal communities’ forest food. European Institute for Food Law series. 2019.

[26] Wu J. Landscape sustainability science: ecosystem services and human well-being in changing landscapes. Landscape Ecology. 2013; 28(6): 999–1023.

[27] Sudarmalik S, Kartodihardjo H, Soedomo S, Adiwibowo S. The state and the development of industrial plantation forest. Jurnal Manajemen Hutan Tropika. 2014; (3): 159–166.

[28] Czerepko J, Goos P, Hilszczanski J. The importance of forests and forest-timber sector as complements to agriculture and as tools for the regional development of Poland. International Journal of Agricultural Resources, Governance and Ecology. 2016; 12 (3): 215–225.

[29] Tokarczyk J, Hansen E. Creating intangible competitive advantages in the forest products industry. Forest Products Journal. 2006; 56 (7/8): 4–13.

[30] Claro DP, de Oliveira Claro PB. Coordinating B2B cross-border supply chains: the case of the organic coffee industry. Journal of Business & Industrial Marketing. 2004; 19(6): 405–414.

[31] Song XD. Analysis of green organic food industry in Heilongjiang Province under the background of comparative advantage. International Journal of Intelligent Information and Management Science. 2020; 9(1): 184–190.

[32] Wang DZ, Zhao DW, Du YQ. Optimization and policy innovation of Chinese green food industrial structure. China Industrial Economics. 2009; (09): 67–76. (in Chinese)

[33] Liu QB, Liu JC, Chen WH. Analysis on the international competitiveness of Chinese forestry Nnuts. Journal of Northwest Forestry University. 2013; 28(01): 265–268. (in Chinese)

[34] Raynolds LT. The globalization of organic agro-food networks. World Development. 2004; 32(5): 725–743.

[35] Zhou R, Chan AHS. Using a fuzzy comprehensive evaluation method to determine product usability: A test case. Work. 2017; 56(1): 21–29. doi: 10.3233/WOR-162473 28035942 PMC5302047

[36] Singer H OZSAHIN S. Employing an analytic hierarchy process to prioritize factors influencing surface roughness of wood and wood-based materials in the sawing process. Turkish Journal of Agriculture and Forestry. 2018; 42(5): 364–371.

[37] Fu W, Luo M, Chen J, Udimal TB. Carbon footprint and carbon carrying capacity of vegetation in ecologically fragile areas: A case study of Yunnan. Physics and Chemistry of the Earth, Parts A/B/C. 2020;120: 102904. doi: 10.1016/j.pce.2020.102904

[38] Wang DZ, Zhao DW, Chen JM. Model of industrial competitive advantage based on an empirical study of green food industry in Heilongjiang Province. China Industrial Economy. 2006; (05): 32–39. (in Chinese)

