# Supplementary material for: The measurement of competitiveness of forest green food industry in Yunnan Province
Source: PLoS One. 2021 Dec 10;16(12):e0261133. doi: 10.1371/journal.pone.0261133 (PMC8664197; doi:10.1371/journal.pone.0261133)
Supplement: S1 File — http://stats.yn.gov.cn/. (Z01) [file pone.0261133.s001.z01 › the Yunnan statistical yearbook (2016-2020)/2016/10/十、交通运输、通信和服务业.doc]

主要统计指标解释

**铁路营业里程** 又称营业长度（包括正式营业和临时营业里程），指办理客货运输业务的铁路正线总长度。凡是全线或部分建成双线及以上的线路，以第一线的实际长度计算；复线、站线、段管线、岔线和特殊用途线以及不计算运费的联络线都不计算营业里程。该指标可以反映铁路运输业基础设施的发展水平，也是计算客货周转量、运输密度和机车车辆运用效率等指标的基础资料。

**公路里程**  指报告期末公路的实际长度。统计范围：包括城间、城乡间、乡（村）间能行驶汽车的公共道路，公路通过城镇街道的里程，公路桥梁长度、隧道长度、渡口宽度。不包括城市街道里程，断头路里程，农（林）业生产用道路里程，工（矿）企业等内部道路里程。统计原则：按已竣工验收或交付使用的实际里程计算；两条或多条公路共同经由同一路段的重复里程，只计算一次。

**内河航道里程** 也称内河通航里程，指在一定时期内，能通航运输船舶及排筏的天然河流、湖泊水库、运河及通航渠道的长度。包括全年季节性通航累计三个月以上的航道，不包括仅供零散流放竹、木排的河道。该指标可以反映内河水运网的规模、水平和发展情况。

**民用航空航线里程** 指统计期间内全部民用航空航线的航线总长度。航线长度指民用航空航线的计费距离。计算航线里程可按重复和不重复两种方法，前者是指各航线长度相加的总和；后者则要扣除各航线之间相同航段重复计算的部分。

**货(客)运量** 指在一定时期内，各种运输工具实际运送的货物（旅客）数量。该指标是反映运输业为国民经济和人民生活服务的数量指标，也是制订和检查运输生产计划、研究运输发展规模和速度的重要指标。货运按吨计算，客运按人计算。货物不论运输距离长短、货物类别，均按实际重量统计。旅客不论行程远近或票价多少，均按一人一次客运量统计；半价票、小孩票也按一人统计。

**货物（旅客）周转量** 指在一定时期内，由各种运输工具运送的货物（旅客）数量与其相应运输距离的乘积之总和。该指标可以反映运输业生产的总成果，也是编制和检查运输生产计划，计算运输效率、劳动生产率以及核算运输单位成本的主要基础资料。计算货物周转量通常按发出站与到达站之间的最短距离，也就是计费距离计算。计算公式为：

货物（旅客）周转量=Σ（货物（旅客）运输量×运输距离）

**邮电业务总量** 指以货币形式表示的邮电企业为社会提供各类邮电服务的总数量，是用于观察邮电业务发展变化总趋势的综合性总量指标。分别按邮政业务总量和电信业务总量统计。邮电业务总量是以各类业务的实物量分别乘以相应的不变单价，得出各类业务的货币量再加总求得。

移动电话用户 指在电信运营企业营业网点办理开户登记手续，通过移动电话交换机进入移动电话网，占用移动电话号码的各类电话用户。包括各类签约用户、智能网预付费用户、无线上网卡用户。

固定电话用户  指在电信企业营业网点办理开户登记手续并已接入固定电话网上的全部电话用户。包括普通电话用户、无线市话用户、公用电话用户、窄带综合业务数字网（N—ISDN）用户、智能网专用接入终端用户等。

城市电话用户 指按行政区划属于中央直辖市、省辖市、地级市、县级市的市区，市郊区及县城区范围内的电话用户数。包括分布在农村地区但以县团级以上建制的独立工矿区、林区、驻军的电话用户。

农村电话用户 指按行政区划属于城市范围以外的乡（镇）、村电话用户。

重点服务业 指交通运输、仓储和邮政业，信息传输、软件和信息技术服务业，租赁和商务服务业，科学研究和技术服务业，水利、环境和公共设施管理业，教育，卫生和社会工作；以及物业管理、房地产中介服务、自有房地产经营活动和其他房地产业等行业年营业收入1000万元及以上，或年末从业人员50人及以上服务业法人单位；居民服务、修理和其他服务业，文化、体育和娱乐业年营业收入500万元及以上，或年末从业人员50人及以上服务业法人单位。

从事服务业活动从业人员平均人数 指报告期内(年度、月度)平均拥有的从事服务业活动的人员数。按“谁用工，谁统计”的原则实施统计，包括参加企业服务业活动的正式人员，劳务派谴人员和临时聘用人员。不包括在本企业领取工资、股息、红利未参加服务业活动的人员。

**Explanatory Notes on Principle Statistical Indicators**

**Length of Railways in Operation** refers to the total length of the trunk line under passenger and freight transportation （including both full operation and temporary operation）. The calculation is based on the actual length of the first line even if this line has a full or partial double track or more tracks, excluding double tracks, station sidings, tracks under the charge of stations, branch lines, special purpose lines and the non payable connecting lines. The length of railways in operation is an important indicator to show the development of the infrastructure for the railway transport, and also the essential data to calculate volume of passenger freight transport, traffic density and utilization efficiency of the locomotives and carriages.

**Length of Highways** refers to the actual length of highways at the end of reference period. It covers public roads running vehicles among cities, city and rural areas, township (villages), highways passing through streets at small cities and towns, length of bridges and tunnels, width of ferry piers. It does not include the length of streets in cities, dead end highways, the length of streets built for agricultural (forest) production and inside factories (mines). It can only be calculated with the actual mileage having been completed, checked and accepted or put into operation. If two or more highways go the same section of the way, the length of the section is only calculated for once.

**Length of Navigable Inland Waterways** it is an indicator reflecting the size and development of inland water network, it refers to the length of the natural rivers, lakes, reservoirs, canals, and ditches open to navigation during a given period, which enables the transport by ships and rafts. It includes the channels open to navigation for over an accumulative 3 months in a year, yet this does not include the river courses, which are only used to float odd logs and bamboo rafts. This indicator can reflect the scale, level and development situation of the inland waterway network.

**Length of Civil Aviation Routes** refers to the length of all routes for regular civil aviation flights, which is used to account the freight, during the period of statistics. There are usually two ways to calculate the route length: duplicated calculation and non-duplicated calculation, the former is the sum of length of all civil aviation routes, and latter should deduct the duplication length of same route among all routes.

**Freight** （**Passenger**） **Traffic** refers to the volume of freight （passenger） transported with various means. The freight （passenger）traffic provides a quantitative measure to show how the transport industry serves the national economy and people, and is also an important indicator for planning the transport industry and for studying the development scale and speed of the transport industry. Freight transport is calculated in tons and passenger traffic is calculated in the number of persons. Despite the type of freight and traveling distance, the freight transport is calculated in the actual weight of the goods, and despite the traveling distance and ticket price, the passenger traffic is calculated by the principle that one person can be counted only once in one travel. The passengers who travel with a half price ticket or a child ticket is also calculated as one person.

**Freight Ton-kilometres** （**Passenger-kilometres**） refer to the sum of the products of the volume of transported cargo （passengers） multiplying by the transport distance. It is an important indicator to reflect the achievement of transportation industry. Normally, the shortest distance between the departure station and the destination station （i.e., the payable distance） is the basis to calculate the freight ton-kilometers. This is an important indicator to show the total results of the transport industry, to prepare and examine the transport plan and to measure the efficiency, the labour productivity and the unit cost of transport. The formula is as follows:

Freight ton-kilometres	freight	distance of

（passenger-kilometres）=Σ（passenger）traffic × transportation

Business Volume of Post and Telecommunications refers to the total amount of post and telecommunication services, expressed in value terms, provided by the post and telecommunications departments for the society. This indicator reflects the overall results of development of postal and telecommunication services. It can be classified as postal services and telecommunication services. Business Volume of Post and Telecommunications is the sum of all services in kind multiplying with the unit price（constant price） to get the total business value.

**Mobile Telephone Subscribers** refer to persons who have gone through registration procedures in the operation points of enterprises engaged in telecommunications and are hence connected with the mobile telephone communication network through the mobile telephone switchboards and occupy mobile phone numbers. Included are various types of subscriber, prepaid users for intelligent network and wireless network card users.

**Local Telephone Subscribers** refer to all subscribers who have gone through registration procedures in the operation points of enterprises engaged in telecommunications and are hence connected to the local telecommunications service provider through fixed line network. Included are general subscribers, wireless local telephone subscribers, public telephones subscribers, N-ISDN subscribers and intelligent network terminal subscribers.

**Urban Telephone Subscribers** refer to the number of telephone subscribers, located at the municipalities directly under the Central Government, cities under the jurisdiction of province, cities at prefecture level, downtown and suburb of city at county level town and county towns according to the administrative division, including subscribers in rural mineral area, forest area, military area that are at or above county level.

**Rural Telephone Subscribers** refer to telephone subscribers, located at the towns and villages outside the coverage of urban areas according to the administrative division.

**Key service industry** refer to following 9 industries:industry of transport,storage and post, industry of information transmission,industry of ware and information technology services,industry of leasing and business, services, Industry of scientific arch,technical services and geologic prospecting, industry of management of water conservancy, environment and public facilities, industry of services to households, repairing and other services, industry of education,health, social works,industry of culture,sports and entertainment. own real estate business activities and other real estate business activities .Enterprises of annual revenue reaches 10 million yuan and above, and number of employees at the end of the year reaches 50 persons of property management services and real estate intermediary services industry are included in key service industry. Legal institutions which carry out enterprises management and accounting system are also included in key service industry.
