# Supplementary material for: The measurement of competitiveness of forest green food industry in Yunnan Province
Source: PLoS One. 2021 Dec 10;16(12):e0261133. doi: 10.1371/journal.pone.0261133 (PMC8664197; doi:10.1371/journal.pone.0261133)
Supplement: S1 File — http://stats.yn.gov.cn/. (Z01) [file pone.0261133.s001.z01 › the Yunnan statistical yearbook (2016-2020)/2016/11/金融保险业.docx]

主要统计指标解释

存款 指企业、机关、团体或居民根据资金必须收回的原则，把货币资金存入银行或其他信贷机构保管并取得一定利息的一种信用活动形式。根据存款对象或性质的不同可划分为单位存款、个人存款、财政性存款、临时性存款、委托存款、其他存款等科目。它是银行信贷资金的主要来源。

贷款 指银行或其他信贷机构根据资金必须归还的原则，按一定利率，为企业、个人等提供资金的一种信用活动形式。我国银行贷款分为短期贷款、中长期贷款、融资租赁、票据融资、各项垫款、境外贷款等。

保险金额 指保险人承担赔偿或者给付保险金责任的最高限额。

保费 指投保人为取得保险人在约定范围内所承担赔偿责任而支付给保险人的费用。

赔款 指保险人根据保险公司合同的规定，向被保险人支付的赔偿保险责任损失的金额。

**Explanatory Notes on Principle Statistical Indicators**

**Deposit**  is a form of credit by which enterprises, institutions, organizations or households can put money into banks and other credit institutions for safekeeping and interest earning under the principle of free withdrawal. According to different depositors, deposits are divided into  unit deposits, personal deposits, fiscal deposits, temporary deposits, entrusted deposits and other deposits. Deposits are major sources of the credit funds of banks.

**Loan**  is a form of credit by which banks and other credit institutions provide funds at certain interest rate to enterprises and individuals in the light of the principle of unconditional repayment. Loans from Chinese banks include short-term loan, medium-term and long-term loans, financial lease, bill financing, various money advanced, foreign loans.

**Amount Insured** refers to the maximum amount that the insurant will get for the claim of the case insured.

**Premium** is the fee paid by the insurant to the insurer to obtain the obligation of compensation from the insurance within the agreed terms.

**Settled Claim** is the compensation paid by the insurer to the insurant in accordance with the insurance contract.
