# Supplementary material for: The measurement of competitiveness of forest green food industry in Yunnan Province
Source: PLoS One. 2021 Dec 10;16(12):e0261133. doi: 10.1371/journal.pone.0261133 (PMC8664197; doi:10.1371/journal.pone.0261133)
Supplement: S1 File — http://stats.yn.gov.cn/. (Z01) [file pone.0261133.s001.z01 › the Yunnan statistical yearbook (2016-2020)/2016/12/经贸.docx]

主要统计指标解释

入境游客  指报告期内来中国（大陆）观光、度假、探亲访友、就医疗养、购物、参加会议或从事经济、文化、体育、宗教活动的外国人、港澳台同胞等游客（即入境旅游人数）。统计时，入境游客按每入境一次统计1人次。入境旅游人数包括入境过夜游客和入境一日游游客。

出境人数（出境游客）  指中国（大陆）居民因公或因私出境前往其他国家、中国香港特别行政区、澳门特别行政区和台湾省观光、度假、探亲访友、就医疗养、购物、参加会议或从事经济、文化、体育、宗教活动的人数（即出境游客）。统计时，出境游客按每出境一次统计1人次。

国内游客  指报告期内在中国（大陆）观光游览、度假、探亲访友、就医疗养、购物、参加会议或从事经济、文化、体育、宗教活动的中国（大陆）居民人数，其出游的目的不是通过所从事的活动谋取报酬。统计时，国内游客按每出游一次统计1人次。

国际旅游(外汇)收入  指入境游客在中国（大陆）境内旅行、游览过程中用于交通、参观游览、住宿、餐饮、购物、娱乐等全部花费。

国内旅游收入(旅游总花费)  指国内游客在国内旅行、游览过程中用于交通、参观游览、住宿、餐饮、购物、娱乐等全部花费。

**Overseas Visitor Arrivals** refer to the number of tourists  of foreigners, Chinese compatriots from Hong Kong, Macao and Taiwan who come to China (mainland) within the reference period for sight-seeing, vacation, visiting relatives, medical treatment, shopping, attending conference, or to engage in economic, cultural, sports and religious activities (namely the number of overseas visitor arrivals). In compiling statistics, each arrival is counted as one person-time. The number of overseas visitor arrivals includes inbound overnight tourists and one-day tourists.

**Number of Chinese Residents Going Abroad (Chinese Outbound Visitors)** refers to the number of Chinese (mainland) residents going to other countries, Hong Kong Special Administrative region, Macao Special Administrative region and Taiwan for on official or private purposes, for sight-seeing, vacation, visiting relatives, medical treatment, shopping, attending conference, or to engage in economic, cultural, sports and religious activities (namely the Chinese outbound visitors). In compiling statistics, each time of leaving is counted as one person-time.

**Number of Domestic Tourists** refers to the number of Chinese (mainland) residents who travel within China (mainland) for sight-seeing, vacation, visiting relatives, medical treatment, shopping, attending conference, or to engage in economic, cultural, sports and religious activities. In compiling statistics, each time of travelling is counted as one person-time.

**Foreign Exchange Earnings from International Tourism**  refer to the total expenditure of foreigners, overseas Chinese, Chinese compatriots from Hong Kong, Macao and Taiwan during their stay in the mainland of China on transportation, sighting, accommodation, food, shopping and entertainment.

**Income from Domestic Tourism**  refer to expenditure of domestic tourists on transportation, sighting, accommodation, food, shopping and entertainment while they travel.
