# Supplementary material for: The measurement of competitiveness of forest green food industry in Yunnan Province
Source: PLoS One. 2021 Dec 10;16(12):e0261133. doi: 10.1371/journal.pone.0261133 (PMC8664197; doi:10.1371/journal.pone.0261133)
Supplement: S1 File — http://stats.yn.gov.cn/. (Z01) [file pone.0261133.s001.z01 › the Yunnan statistical yearbook (2016-2020)/2016/13/十三、教育、科技和文化.doc]

主要统计指标解释

普通高等学校 指按照国家的审批程序批准举办，通过全国统一招生考试，招收高级中等学校毕业和具有同等学历者，实施高等教育,培养高等专门人才的学校。包括大学、专门学院、专科学校和短期职业大学。

成人高等学校 指按国家规定的审批程序批准举办，招收职业高中毕业或同等学历者,利用多种形式对成人实施高等教育，培养相当于普通高等专科或本科毕业水平的专门人才的学校。包括广播电视大学、职工高等学校、农民高等学校、干部管理学院、教育学院、独立函授学院以及普通高等学校举办的函授、夜大学等。

小学学龄儿童入学率 指调查范围内已入小学学习的学龄儿童占该地区校内外学龄儿童总数（包括弱智儿童在内，但不包括盲聋哑儿童）的比重。计算公式为：

小学学龄儿童入学率=已入学的小学学龄儿童数/校内外小学学龄儿童总数×100%

科技活动 指在自然科学、农业科学、医药科学、工程与技术科学、人文与社会科学领域（简称科学技术领域）中，与科技知识的产生、发展、传播和应用密切相关的有组织的活动。可分为研究与试验发展（R&D）、研究与试验发展成果应用及相关的科技服务三类活动。该定义是联合国教科文组织考虑成员国特别是发展中国家开展科技统计工作的需要，而对科技活动所做的统计界定。

科技活动人员 指直接从事科技活动，以及专门从事科技活动管理和为科技活动提供直接服务，累计的实际工作时间占全年制度工作时间10%及以上的人员。（1）直接从事科技活动的人员，包括：在独立核算的科学研究与技术开发机构、高等学校、各类企业及其他事业单位内设的研究室、实验室、技术开发中心及中试车间（基地）等机构中从事科技活动的研究人员、工程技术人员、技术工人及其他人员，虽不在上述机构工作，但编入科技活动项目（课题）组的人员，科技信息与文献机构中的专业技术人员，从事论文设计的研究生等。（2）专门从事科技活动管理和为科技活动提供直接服务的人员，包括独立核算的科学研究与技术开发机构、科技信息与文献机构、高等学校、各类企业及其他事业单位主管科技工作的负责人，专门从事科技活动的计划、行政、人事、财务、物资供应、设备维护、图书资料管理等工作的各类人员，但不包括保卫、医疗保健人员、司机、食堂人员、茶炉工、水暖工、清洁工等为科技活动提供间接服务的人员。该指标用来反映投入科技活动人力的规模。

科学家与工程师 指科技活动人员中具有高、中级技术职称（职务）的人员和不具有高、中级技术职称（职务）的大学本科及以上学历人员。该指标用来反映投入科技活动人力的素质。

研究与试验发展（R&D） 指在科学技术领域，为增加知识总量，以及运用这些知识去创造新的应用进行的系统的创造性的活动，包括基础研究、应用研究、试验发展三类活动。国际上通常采用R&D活动的规模和强度指标反映一国的科技实力和核心竞争力。

专业技术人员 指从事专业技术工作和专业技术管理工作的人员，即企事业单位中已经聘任专业技术职务从事专业技术工作和专业技术管理工作的人员，以及未聘任专业技术职务、现在专业技术岗位上工作的人员。包括工程技术人员、农业技术人员、科学研究人员、卫生技术人员、教学人员。

科技活动经费筹集 指从各种渠道筹集到的计划用于科技活动的经费，包括政府资金、企业资金、事业单位资金、金融机构贷款、国外资金和其他资金等。反映各社会经济主体对促进科技进步所做的努力。

政府资金 指从各级政府部门获得的计划用于科技活动的经费，包括科学事业费、科技三项费、科研基建费、科学基金、教育等部门事业费中计划用于科技活动的经费，以及政府部门预算外资金中计划用于科技活动的经费等。

科技活动经费内部支出 指报告年内用于科技活动的实际支出，包括劳务费、科研业务费、科研管理费、非基建投资购建的固定资产、科研基建支出以及其他用于科技活动的支出。不包括生产性活动支出、归还贷款支出及转拨外单位支出。反映科技投入实际完成情况。

劳务费 指以货币或实物形式直接或间接支付给从事科技活动人员的劳动报酬及各种费用。包括各种形式的工资、津贴、奖金、福利、离退休人员费用、人民助学金等。反映改善科技人员的待遇情况。

固定资产购建费 指报告年内使用非基建投资购建的固定资产和用于科研基建投资的实际支出额，即固定资产实际支出和科研基建投资实际完成额之和。固定资产是指长期使用而不改变原有实物形态的主要物资设备、图书资料、实验材料和标本以及其他设备和家具、房屋、建筑物。反映用于改善科研条件和科研手段方面的投入情况。

新产品 指采用新技术原理、新设计构思研制、生产的全新产品，或在结构、材质、工艺等某一方面比原有产品有明显改进，从而显著提高了产品性能或扩大了使用功能的产品，既包括政府有关部门认定并在有效期内的新产品，也包括企业自行研制开发，未经政府有关部门认定，从投产之日起一年之内的新产品。用来反映科技产出及对经济增长的直接贡献。

专利 是专利权的简称，是对发明人的发明创造经审查合格后，由专利局依据专利法授予发明人和设计人对该项发明创造享有的专有权。包括发明、实用新型和外观设计。反映拥有自主知识产权的科技和设计成果情况。

发明 指对产品、方法或者其改进所提出的新的技术方案。是国际通行的反映拥有自主知识产权技术的核心指标。

实用新型 指对产品的形状、构造或者其结合所提出的适于实用的新的技术方案。反映具有一定技术含量的技术成果情况。

外观设计 指对产品的形状、图案、色彩或者其结合所做出的富有美感并适于工业上应用的新设计。反映拥有自主知识产权的外观设计成果情况。

文化事业机构 指从事专业文化工作和为专业文化工作服务的单独核算、独立建制的单位。不包括半工半艺、半农半艺的业余剧团。

艺术表演观众人数 指售票、包场演出或民族地区免费演出的艺术表演观众人数。不包括彩排审查和内部观摩演出的观看人次数。

等级运动员人数 指经考核正式批准授予等级运动员称号的人数。运动员等级分为国际级运动健将、运动健将、一级运动员、二级运动员、三级运动员、少年级运动员。

等级裁判员人数 指经考核正式批准授予等级裁判员称号的人数。裁判员等级分为国际裁判、国家级裁判、一级裁判、二级裁判、三级裁判。

**Explanatory Notes on Principal Statistical Indicators**

**Regular Institutions of Higher Learning** refer to the educational institutions set up according to the government evaluation and approval procedures, enrolling graduates from senior high schools and providing higher education courses and training senior professionals. They include fulltime universities and colleges, junior colleges and short-term schools for professional training.

**Institutions of Higher Learning for Adults** refer to the educational institutions, set up in line with relevant rules approved by the government, enrolling staff and workers with senior high school or equivalent education, and providing higher education courses in many forms of full time, part time, spare time, or correspondence for adults. Professionals thus trained receive a qualification equivalent to graduates studying regular courses at regular universities, colleges and professional colleges. Institutions of higher learning for adults include Radio and TV universities, colleges for staff and workers and for farmers, management colleges for cadres, education colleges, independent correspondence college and correspondence schools, night schools and the like run by regular institutions of higher learning.

**Enrollment Rate of Primary School-age Children** refers to the proportion of school-age children enrolled at school to the total number of school-age children both at and out of school （including retarded children, but excluding blind, deaf and mute children）. The formula is as follows:

Enrollment Rate of Primary School-age Children = （Total Primary School-age Children at School）/（Total Primary School-age Children Both at and out of School）×100%

**Scientific and Technological Activities** （**S&T Activities**） refer to organized activities which are closely related to the creation, development, dissemination and application of scientific and technological knowledge in the fields of natural sciences, agricultural science, medical science, engineering and technological science, humanities and social sciences （referred to as scientific and technological fields）. S&T activities can be divided into three categories: research and development （R&D） activities, application of R&D results, and related S&T services. This statistical definition is made by UNICHIEF for scientific and technological activities to meet the need for carrying out statistical work in this field in its member countries, especially those developing countries.

**Personnel Engaged in S&T Activities** refer to personnel directly engaged in S&T activities, in the management of S&T activities, and in providing direct service to S&T activities, who spend over 10% of the total working hours in a year in S&T activities. 1）.Personnel directly engaged in S&T activities include researchers, engineers, technicians and other related personnel engaged in S&T activities in independent-accounting R&D institutions, institutions of higher learning, and in research rooms and institutes, laboratories, technological development centers and central experiment workshops under enterprises and institutions. Also included are people working in S&T research project teams, professional and technical personnel working in S&T information and literature institutions, and graduate students working on the design of their theses. 2）.Personnel engaged in the management of S&T activities and in providing direct service to S&T activities include administrative personnel responsible for S&T activities in independent-accounting R&D institutions, S&T information and literature institutions, institutions of higher learning, and enterprises and institutions where S&T activities are undertaken. Also included are people responsible for the planning, administration, personnel management, financial management, logistics supply, equipment maintenance, information and library management that are related to S&T activities. People providing indirect services are excluded, such as security personnel, medical staff, drivers, plumbers, cleaners and those providing food and related services. This indicator reflects the scale of personnel engaged in S&T activities.

**Scientists and Engineers** refer to persons engaged in S&T activities who have obtained technical or professional titles of senior and middle rank, and those without such title but have completed university or higher education. This indicator reflects the quality of personnel engaged in S&T activities.

**Research and Development** （**R&D**） refers to systematic and creative activities in the field of science and technology aiming at increasing and using the knowledge for new application. R&D falls into 3 categories of activities: basic research, applied research and experiment and development. The scale and intensity of R&D are widely used internationally to reflect the strength of S&T and the core competitiveness of a country in the world.

**Professional and Technical Personnel** refer to persons engaged in professional and technical work or in the management of professional and technical activities, i.e., personnel with professional or technical titles who are engaged in professional and technical work or in the management of professional and technical activities, and personnel without professional or technical titles but working on professional or technical posts in enterprises and institutions. They include professionals and technicians working in the fields of engineering, agriculture, scientific research, health care and education.

**Funding for S&T Activities** refers to funds obtained from various sources for S&T activities, including government funds, self-raised funds by enterprises, self-raised funds by institutions, loans from financial institutions, foreign funds and other funds. This indicator reflects the efforts made by various social economic entities in promoting the development of S&T.

**Government Funds** refer to funds obtained from government departments at all levels for S&T activities, including funds for scientific undertakings, 3 kinds of funds for S&T activities, funds for capital construction for scientific research, science funds, funds from educational expenditures by educational departments for S&T activities, and extra-budgetary funds from government departments for S&T activities.

**Internal Expenditures on S&T Activities** refer to the actual expenditures on S&T activities during the report year, including service charges, operating expenses on research activities, overhead charges on research, fixed assets excluded in the investment in capital construction, expenditures on capital construction for scientific research, and other expenditures on S&T activities. Not included are expenditures on production activities, repayment of loans and transfer expenditures. This indicator reflects the real completion of input in S&T.

**Service Charges** refer to direct or indirect payments, in cash or in kind, made to personnel engaged in S&T activities as remuneration and other charges, including salaries, subsidies, bonus, benefits, retirement pensions, stipends, etc. This indicator reflects the improvement of treatment toward S&T personnel.

**Expenditure on Purchase and Construction of Fixed Assets** refers to the fixed assets purchased or constructed by using funds excluded in the investment in capital construction and the actual expenditures on capital construction for scientific research within the report year, i.e. the sum total of the actual expenditures on fixed assets and the actual investments in capital construction for scientific research. Fixed asset refers to main materials and equipment, literature and documents in libraries, materials for experiments, specimen, instruments, furniture, buildings and constructions that can be used for a long time without changing their original forms and shapes. This indictor reflects the input in improving the conditions and means of scientific research.

**New Products** refer to new products produced with new technology and design, or products that represent noticeable improvement in terms of structure, material, or production process so as to improve significantly the character or function of the older versions. They include new products certified by relevant government departments within the period of certification, and those designed and produced by enterprises within a year without certification by government departments. This indicator reflects the S&T output and its direct contribution to economic growth.

**Patent** is an abbreviation for patent right and refers to the exclusive right of ownership by the inventors or designers for their creations or inventions, conferred by the patent offices after the due process of assessment and approval in accordance with the Patent Law. Patent is granted for inventions, utility models and designs. This indicator reflects the achievements of S&T and design with independent intellectual property.

**Inventions** refer to the new technical proposals on products or methods or their modifications. This is universal core indicator reflecting the technologies with independent intellectual property.

**Utility Models** refer to the practical and new technical proposals on the shape and structure of the product or their combination. This indicator reflects the technological results with certain technical content.

**Designs** refer to the aesthetic and industrially applicable new designs for the shape, pattern and color of the product or their combinations. This indicator reflects the exterior design achievements with independent intellectual property.

**Cultural Institutions** refer to entities which have their own organizational systems and independent accounting systems and specialize in or serve cultural development, excluding other establishments run by these cultural institutions and amateur cultural groups established by various departments.

**Number of Spectators at Art Performance** refers to the number of attendants at commercial shows, completely booked shows or a free show offered in minority nationality areas, and does not include the number of spectators at rehearsals of examination and internal shows for observation.

**Number of Athletes in Grades** refers to the number of athletes who have been conferred titles after examination. The titles of athletes include international masters of sports, masters of sports, first-grade, second-grade and third-grade sportsmen and young athletes.

**Number of Referees in Grades** refers to the number of referees who have been conferred titles after examination. They are classified as international-level referees, national-level referees and referees of the first, second and third grades.
