# Supplementary material for: The measurement of competitiveness of forest green food industry in Yunnan Province
Source: PLoS One. 2021 Dec 10;16(12):e0261133. doi: 10.1371/journal.pone.0261133 (PMC8664197; doi:10.1371/journal.pone.0261133)
Supplement: S1 File — http://stats.yn.gov.cn/. (Z01) [file pone.0261133.s001.z01 › the Yunnan statistical yearbook (2016-2020)/2016/14/十四、卫生、体育和社会管理.doc]

主要统计指标解释

卫生机构 指从卫生行政部门取得“医疗机构执业许可证”，或从民政、工商行政、机构编制管理部门取得法人单位登记证书，为社会提供医疗保健、疾病控制、卫生监督服务或从事医学科研和教育等工作的单位。卫生机构包括医院、疗养院、社区卫生服务中心（站）、卫生院、门诊部、诊所（卫生所、医务室）、急救中心（站）、采供血机构、妇幼保健院（所、站）、专科疾病防治院（所、站）、疾病预防控制中心（防疫站）、卫生监督所、卫生监督检验（监测、检测）机构、医学科研机构、医学在职培训机构、健康教育所(站)等其他卫生机构。

医疗机构 指从卫生行政部门取得“医疗机构执业许可证”的机构，包括医院、疗养院、社区卫生服务中心（站）、卫生院、门诊部、诊所（卫生所、医务室）、妇幼保健院（所、站）、专科疾病防治院（所、站）、急救中心（站）和临床检验中心。

社区卫生服务中心（站） 指为本社区居民提供预防、医疗、保健、康复、健康教育、计划生育技术服务等的基层卫生机构。包括社区卫生服务中心和社区卫生服务站。

卫生人员 指在医疗、预防保健、医学科研和在职教育等卫生机构工作的职工，包括卫生技术人员、其他技术人员、管理人员和工勤人员。

卫生技术人员 包括执业（助理）医师、注册护士、药剂人员、检验和影像人员等卫生专业人员，不包括从事管理工作的卫生技术人员（一律计入管理人员）。

执业医师 指具有“医师执业证”及其“级别”为“执业医师”且实际从事医疗、预防保健工作的人员，不包括实际从事管理工作的执业医师。执业医师类别分为临床、中医、口腔和公共卫生。

执业助理医师 指具有“医师执业证”及其“级别”为“执业助理医师”且实际从事医疗、预防保健工作的人员，不包括实际从事管理工作的执业助理医师。执业助理医师类别同样分为临床、中医、口腔和公共卫生四类。

城市社会福利事业单位 包括社会福利院、儿童福利院和民政部门所属的精神病院等。

城乡社会救济费社会救济 是指国家或集体用于生活困难人员的财物支出。本指标包括城镇社会救济费、乡村社会救济费、精简退职的老职工救济费。

1.城镇社会救济费 包括民政部门支出的城镇困难户救济费和机关企事业单位支付的职工生活困难补助费。

2.乡村社会救济费 包括民政部门支出的农村五保户、困难户及麻风病人救济费。本指标包括农村集体支付的散居五保户、贫困户救济折款（包括实物）。

3.精减退职的老职工救济费 指民政部门支出的精减退职的老职工救济费（包括按原工资40%发给的救济费和其他困难救济费）。

自然灾害受灾人数 指遭受自然灾害人数中的成灾人数。所谓成灾是指遭受自然灾害，作物收成减产三成以上的单位，这种单位的全部农业人口即为成灾人口。

优抚事业单位 指革命残废军人休养院、荣复军人疗养院和复退军人精神病院、光荣院。

优抚对象 优抚是指我国人民群众对革命烈士家属、病故革命军人家属、革命残废军人、革命残废工作人员以及参战负伤致残的民兵、民工的优待和对这些人的抚恤。“优抚对象”包括烈军属、复退军人、革命残废人员。

优抚事业费 指民政部门开支的抚恤事业费。包括牺牲费、烈军属及复员退伍军人补助费、退伍军人安置费、优抚事业单位经费和其他抚恤事业费。

城镇职工基本养老保险

1.（参保）职工人数 指报告期末按照国家法律、法规和有关政策规定参加基本养老保险并在社保经办机构已建立缴费记录档案的职工人数，包括中断缴费但未终止养老保险关系的职工人数（不包括只登记未建立缴费记录档案的人数）。

2.（参保）离退休人员人数 指报告期末参加基本养老保险的离休、退休和退职人员的人数。

3.基本养老保险基金收入 指根据国家有关规定，由纳入基本养老保险范围的缴费单位和个人按国家规定的缴费基数和缴费比例缴纳的养老保险基金，以及通过其他方式取得的形成基金来源的收入（包括单位和职工个人缴纳的基本养老保险费、基本养老保险基金利息收入、上级补助收入、下级上解收入、转移收入、财政补贴和其他收入）。

4.基本养老保险基金支出 指按照国家政策规定的开支范围和开支标准从养老保险基金中支付给参加基本养老保险的个人的养老金、丧葬抚恤补助，以及由于保险关系转移、上下级之间调剂资金等原因而发生的支出（包括离休金、退休金、退职金、各种补贴、医疗费、死亡丧葬补助费、抚恤救济费、社会保险经办机构管理费、补助下级支出、上解上级支出、转移支出、其他支出等）。

5.基本养老保险基金累计结余 指截止报告期末基本养老保险基金收支相抵后的累计余额。

基本医疗保险

1.参保人数 指报告期末按国家有关规定参加基本医疗保险的人数（包括参加保险的职工人数和退休人员人数）。

2.基金收入 指根据国家有关规定，由纳入基本医疗保险范围的缴费单位和个人，按国家规定的缴费基数和缴费比例缴纳的基金，以及通过其他方式取得的形成基金来源的款项（包括单位缴纳的社会统筹基金收入、个人缴纳的个人账户基金收入、财政补贴收入、利息收入、其他收入）。

3.基金支出 指按照国家政策规定的开支范围和开支标准从社会统筹基金中支付给参加基本医疗保险的职工和退休人员的医疗保险待遇支出，和从个人账户基金中支付给参加基本医疗保险的职工和退休人员的医疗费用支出，以及其他支出（包括住院医疗费用支出、门急诊医疗费用支出、个人账户基金支出、其他支出）。

4.基金累计结余 指截止报告期末基本医疗保险的社会统筹和个人账户基金累计结余金额（包括银行存款、财政专户、债券投资和其他）。

失业保险

1.参保人数 指报告期末按照国家法律、法规和有关政策规定参加了失业保险的城镇企业、事业单位的职工及地方政府规定参加失业保险的其他人员的人数。

2.失业保险基金收入 指按照规定从企业、事业及其他单位筹集的失业保险费及其他并入失业保险基金收入的总额。包括单位和个人缴纳的失业保险费、失业保险基金利息收入、上级补助收入、下级上解收入、转移收入、财政补贴和其他收入。

3.失业保险基金支出 指报告期内为保障失业人员和下岗职工基本生活、促进其再就业等支出的基金总额（包括失业救济金、医疗费、死亡丧葬补助费、抚恤救济费、转业训练费支出、失业保险经办机构管理费、补助下级支出、上解上级支出、转移支出和其他支出）。

4.基金累计结余 指截止报告期末失业保险基金收支相抵后的累计余额。

工伤保险

1.参加保险人数: 指报告期末依据国家有关规定参加工伤保险的职工人数。

2.享受保险待遇人数 指劳动者因工负伤致残、死亡或因患职业病致残，根据有关规定享受工伤保险待遇职工或供养直系亲属人数（包括伤残人数、职业病人数、因工死亡人数、供养直系亲属人数）。

3.基金收入 指根据国家有关规定，由参加工伤保险的单位按国家规定的缴费基数和缴费比例缴纳的工伤保险基金，以及通过其他形式取得的形成基金来源的款项（包括单位缴纳的社会统筹基金收入、财政补贴收入、利息收入、其他收入）。

4.基金支出 指按照国家政策规定的开支范围和开支标准从工伤保险基金中支付给参加工伤保险的人员及供养直系亲属工伤保险待遇支出及其他支出（包括工伤医疗费、伤残补助金、工亡补助金、护理费、丧葬补助费、工伤预防费用、职业康复费用和其他支出）。

5.基金累计结余 指截止报告期末工伤保险基金累计结余金额（包括银行存款、财政专户、债券投资和其他）。

生育保险

1.参保人数 指报告期末依据有关规定参加生育保险的职工人数。

2.基金收入 指根据国家有关规定，由参加生育保险的单位按照国家规定的缴费基数和缴费比例缴纳的生育保险基金，以及通过其他方式取得的形成基金来源的款项（包括单位缴纳的基金收入、利息收入和其他收入）。

3.基金支出 指按照国家政策规定的开支范围和开支标准，从生育保险基金中支付给参加生育保险的职工，因妊娠、分娩和计划生育手术而享受的待遇及其他支出（包括生育津贴、医疗费用支出及其他支出）。

4.基金累计结余 指截止报告期末生育保险基金累计结余金额（包括银行存款、财政专户、债券投资和其他）。

律师 指受聘参加法律顾问工作，担任法律顾问、刑（民）事代理人、刑事辩护人，办理非诉讼事件，解答法律询问，代写法律事务文书等主要从事律师业务的专职法律工作者和兼职律师。

公证人员 指在国家公证机关依法办理公证事务的司法人员，包括公证员、助理公证员和公证处公证的其他人员。

**Explanatory Notes on Principal Statistical Indicators**

**Health Care Institutions** refer to the units which have been qualified the Certification of Health Care Institution by the administration of public health, or qualified the Certification of Corporate Unit by the civil affairs, administration for industry and commerce, commission office for public sector reform, and engaging in medical care, disease prevention and control, health supervision and inspection, medicine research and health education, etc., including: hospitals, sanatoriums, community health service centers （stations）, health centers, clinics （health stations and infirmaries）, first-aid centres （stations）, blood gathering and supplying institutions, women and children care agencies （centres and stations）, special disease prevention and curing agencies （centres and stations）, disease prevention and control centres （epidemic prevention stations）, health supervision and inspection agencies, sanitary inspection institutions, medicinal scientific research and on-job training institutions, health education centres and so on.

**Medical Organizations** refer to the institutions which have been qualified the Certification of Health Care Institution by the administration of public health, including: hospitals, sanatoriums, community health service centers （stations）, health centers, clinics （health stations and infirmaries）, women and children care agencies （centres and stations）, special disease prevention and curing agencies （centres and stations）, first-aid centres （stations） and clinic inspection centers.

**Community Health Service Centres** （**stations**） refer to the primary units that provide the health care for community residents, such as disease prevention and control, medical treatment, health care, rehabilitation, health education, family planning technical services, including community health service centres and community health service stations.

**Health Care Employee** refer to all employee engaged in the health care institutions, such as medical organizations, disease prevention and control centres, health care agencies, medicinal scientific research and on-job training institutions, including medical technical personnel, other technical personnel, manager and labour.

**Medical Technical Personnel** refer to the professional staff engaged in health care, including licensed （assistant） doctors, registered nurse, pharmacists, laboratory technician, and imaging staff, excluding the medical technical personnel engaged in management job （included as the management staff）.

**Licensed Doctors** refer to the medical workers who have obtained the licenses of qualified doctors and are employed in medical treatment, disease prevention or healthcare institutions, excluding the licensed doctors engaged in management job. The classification of licensed doctors is clinician, Chinese medicine, dentist and public health.

**Licensed Assistant Doctors** refer to the medical workers who have obtained the licenses of qualified assistant doctors and are employed in medical treatment, disease prevention or healthcare institutions, excluding the licensed assistant doctors engaged in management job. The classification of licensed assistant doctors is clinician, Chinese medicine, dentist and public health.

**Urban Social Welfare Institutions** include social welfare institutions, children welfare institutions, metal hospitals subordinated to the civil affairs departments.

**Urban and Rural Social Relief Funds** refer to the financial expenditure for the needy by the state or collectives. This indicator includes urban social relief funds, rural social relief funds and relief funds for reduced or resigned old staff and workers.

**1.Urban social relief funds** include the relief funds paid by the civil affairs departments to urban needy households and the living allowances paid by government departments, enterprises and institutions to staff and workers with financial difficulties.

**2.Rural social relief funds** include the relief funds paid by the civil affairs departments to rural households （of infirm and childless old persons） enjoying the five guarantees, needy households and lepers. This indicator also includes the relief in money and in kind paid by rural collectives to scattered-living households （of infirm and childless old persons） enjoying the five guarantees and needy households.

**3.Relief funds for reduced** **or resigned old staff and workers** refer to the relief funds paid by the civil affairs departments to reduced or resigned old staff and workers （including the relief funds paid at 40% of their original wages and other relief funds）.

**Number of Natural Disaster Victims** refers to the number of people stricken by natural disaster of a certain extent. The so-called “disaster of a certain extent” means any natural disaster that causes crop yield to reduce by over 30% and the total agricultural population hit by it is the stricken population.

**Institutions for Special Care** refer to rest homes for disabled revolutionary servicemen, the sanatoriums for honorably retired servicemen and mental homes or honor homes for retired servicemen.

**Persons Enjoying Special Care** Special care means the special treatment and compensation given by the state to family members of revolutionary martyrs, family members of revolutionary servicemen died of illness, disabled revolutionary servicemen, disabled revolutionary working staff and militias and laborers wounded and disabled in war. “Person Enjoying Special Care” refers to family members of revolutionary martyrs, retired servicemen and disabled revolutionary persons.

**Funds for Special Care** refer to the funds spent by the civil affairs departments for special care, which include sacrifice pensions, allowances for family members of martyrs and retired servicemen, placement allowances for retired servicemen, funds of institutions for special care and other special funds.

**Basic Pension Insurance**

**1.Number of staff and workers covered** refers to staff and workers participating in the basic pension insurance programme according to national laws, regulations and related policies at the end of the reference period, who have already had payment records in social security management agencies, including those who have interrupt payment without terminating the insurance programme. Those who have registered in the programme but with no payment records are not included.

**2. Number of retirees participating in the basic pension insurance programme** refers to the number of retirees participating in basic pension insurance programmes by the end of the reference period.

**3. Revenue of the basic pension insurance programme** refers to payments made by employers and individuals participating in the pension insurance programme in accordance with the basis and proportion stipulated in State regulations, and income from other sources that become source of pension insurance fund, including the premium paid by employers and staff and workers, interest income, subsidies from higher level agencies, income as transfer from subordinate agencies, transferred income, government financial subsidies and other income.

**4. Expenditure of basic pension insurance programme** refers to payment made on pensions and funeral subsidies to those retired and resigned people covered in pension insurance programmes according to related national policies on scopeand standard of expenditure. Also included are expenditure which arises due to shift of the insurance relationship or adjustment of funds among agencies. More specifically, included are pensions for resigned people, pensions for retired people, pension for people quitting jobs, various subsidies, medical fees, funeral subsidies, compensation payments, management fees for social security agencies, expenses on subsidies to lower subordinates, expenses as transfer to agencies at higher level, transferred expenditure and other expenditure.

**5. Balance of basic pension insurance programme** refers to the balance of basic pension insurance funds at the end of the reference period after deducting expenses from revenue.

**Basic Medical Care Insurance**

**1. Number of people participating in the insurance programme** refers to people participating in the basic medical care insurance programme according to related regulations as at the end of reference period, including number of staff and workers and retirees participating in this insurance programme.

**2. Revenue of the insurance programme** refers to payments made by employers and individuals participating in the medical care insurance programme in accordance with the basis and proportion stipulated in State regulations, and income from other sources that become source of medical insurance fund, including income of social comprehensive funds paid by employers, income from individual accounts, government financial subsidies, interest income and other income.

**3. Expenditure of the insurance programme** refers to payment made from social comprehensive funds to those retired and resigned people covered in basic medical care insurance within the scope and standards of expenditure according to related national policies, and medical care payment made from individual accounts to staff and workers and retirees, and other expenses, including medical expenses of hospital inpatients, medical expenses for outpatients and emergency patients, payment from individual accounts and other expenditure.

**4. Balance of the basic medical care insurance programme** refer to the balance of medical care insurance of social comprehensive funds and individual accounts at the end of the reference period, including bank savings, special fiscal accounts, investment in bonds and others.

**Unemployment Insurance**

**1. Number of people covered** refers to staff and workers in urban enterprises or institutions who have participated in the unemployment insurance programme according to relevant policies and regulations, and other people who have participated according to local government regulations, as at the end of reference period.

**2. Revenue of the unemployment insurance programme** refers to payments made by employers and individuals participating in unemployment insurance programme in accordance with relevant regulations and other income contributed to this programme, including unemployment insurance premium made by employers and individuals, interest income, subsidies from higher level agencies, income as transfer from subordinate agencies, transferred income, government financial subsidies and other income.

**3. Expenditure of the unemployment insurance programme** refers to total expenses during the reference period to guarantee the basic livelihood of unemployed people and laid-off staff and workers and to encourage their re-employment. Included are unemployment relief, medical fees, funeral subsidies, compensation payments, training expenses, management fees for unemployment insurance agencies, subsidies to lower level agencies, expenses as transfer to higher level agencies, transferred expenditure and other expenditure.

**4. Balance of the unemployment insurance programme** refers to the balance of revenue of the programme after deducting expenses at the end of the reference period.

**Work Injury Insurance**

**1. Number of people covered** refers to staff and workers who have participated in the work injury insurance programme according to relevant national regulations.

**2. Number of beneficiaries** refers to staff and workers and their direct dependents who can, in accordance with relevant regulations, benefit from work injury insurance, as a result of work injury leading to disability or death of the staff/worker, or occupational disease leading to disability. Included in this category are number of injured and disabled people, number of people with occupational diseases, number of deaths at work places, and number of direct dependents.

**3. Revenue of the work injury insurance programme** refers to payments made by employers participating in the work injury insurance programme in accordance with the basis and proportion stipulated in State regulations, and income from other sources that become source of work injury insurance fund, including income of social comprehensive funds paid by employers, government financial subsidies, interest income and other income.

**4. Expenditure of the work injury insurance programme** refers to payments made from work injury insurance funds to those who participated in the work injury insurance programme and their direct dependents within the scope and standards of expenditure according to related national policies, and other expenditure, including medical fees for work injury, injury and disability subsidies, death subsidies, nursing fees, funeral subsidies, injury prevention fees, occupational rehabilitation fees and other expenditure.

**5. Balance of the work injury insurance programme** refers to the balance of the work injury funds at the end of the reference period, including bank savings, special fiscal account, investment in bonds and others.

**Maternity Insurance**

**1. Number of people covered** refers to staff and workers who have participated in the maternity insurance programme according to relevant regulation at the end of the reporting period.

**2. Revenue of maternity insurance** refers to payments made by employers participating in the maternity insurance programme in accordance with the basis and proportion stipulated in State regulations, and income from other sources that become source of maternity insurance fund, including income of funds paid by employers, interest income and other income.

**3. Expenditure of the maternity insurance programme** refers to payments made from maternity insurance funds to staff and workers who participate in the maternity insurance programme within the scope and standards of expenditure in accordance with related national policies, expenses paid for pregnancy, child delivery or surgeries related to family planning, and other expenditure, including allowance for child bearing, medical fees and other expenditure.

**4. Balance of the maternity programme** refers to the balance of the maternity insurance funds at the end of reference period, including bank savings, special fiscal account, investment in funds and others.

Lawyers refer to the full-time legal workers and the part-time lawyers engaged in the law practices, employed by legal counseling firms to act as legal advisers, agents in criminal or civil lawsuits and defenders in criminal lawsuits, to handle non-lawsuit legal matters, advise on matters of law and write legal papers for others.

**Notary Personnel** refer to the judicial officers who handle the notary affairs under the law in the national notary organs, including notaries, assistant notaries and other workers who handle the notary affairs in the notary offices.
