# Supplementary material for: The measurement of competitiveness of forest green food industry in Yunnan Province
Source: PLoS One. 2021 Dec 10;16(12):e0261133. doi: 10.1371/journal.pone.0261133 (PMC8664197; doi:10.1371/journal.pone.0261133)
Supplement: S1 File — http://stats.yn.gov.cn/. (Z01) [file pone.0261133.s001.z01 › the Yunnan statistical yearbook (2016-2020)/2016/15/十五、人口与就业.doc]

主要统计指标解释

人口数 指一定时点、一定地区范围内有生命的个人总和。年度统计的年末人口数指每年12 月31 日24 时的人口数。年度统计的全国人口总数内未包括香港、澳门特别行政区和台湾省以及海外华侨人数。

城镇人口和乡村人口 城镇人口是指居住在城镇范围内的全部常住人口；乡村人口是除上述人口以外的全部人口。

人口密度 指一定时点一定地区的人口数与该地区的面积数之比,即一定时点的单位土地面积上的人口数通常以每平方公里的居民人数来表示。计算公式：

人口密度（人/平方公里）=该地区的人口数/该地区的土地面积

出生率 出生率（又称粗出生率）指一定时期内（通常为一年内）平均每千人所出生的人数的比例，一般用千分率表示。计算公式：出生率（‰）=年出生人数/年平均人数×1000‰

出生人数是指活产婴儿,即胎儿脱离母体时(不管怀孕月数)有过呼吸或其他生命现象。年平均人数是年初、年末人口数的平均数,也可用年中人口数代替。

死亡率 指在一定时期内(通常为一年内)一定地区的死亡人数与同期平均人数(或期中人数)之比,一般用千分率表示。计算公式：

死亡率（‰）=年死亡人数/年平均人数 ×1000‰

人口自然增长率 在一定时期内(通常为一年内)人口自然增加数(出生人数减死亡人数)与平均人数(或期中人数)之比,一般用千分率表示。计算公式：

人口自然增长率=（本年出生人口数-本年死亡人口数）/年平均人口数×1000%

人口自然增长率（‰）=人口出生率-人口死亡率

性别比 反映男性与女性人口比例的指标,指在总人口中或各年龄组人口中,男性人数与女性人数之比。

通常以每100 个女性人口相对应的男性人口数。计算公式：

性别比=男性人口/女性人口×100

农业、非农业人口 根据公安部门下发的“户口簿”的户口性质统计。

经济活动人口 指年龄在16 周岁及以上，有劳动能力，参加或要求参加社会经济活动的人口。包括就业人员和失业人员。

就业人员 指年龄在16 周岁及以上，从事一定社会劳动并取得劳动报酬或经营收入的人员。这一指标反映了一定时期内全部劳动力资源的实际利用情况，是研究我国基本国情国力的重要指标。

单位就业人员 指在各级国家机关、政党机关、社会团体及企业、事业单位中工作，取得工资或其他形式的劳动报酬的全部人员。包括在岗职工、再就业的离退休人员、民办教师以及在各单位中工作的外方人员和港澳台方人员、兼职人员、借用的外单位人员和第二职业者。不包括离开本单位仍保留劳动关系的职工。各单位的就业人员反映了各单位实际参加生产或工作的全部劳动力。

城镇私营和个体就业人员 城镇私营就业人员指在工商管理部门注册登记，其经营地址设在县城关镇（含县城关镇）以上的私营企业就业人员，包括私营企业投资者和雇工。城镇个体就业人员指在工商管理部门注册登记，并持有城镇户口或在城镇长期居住，经批准从事个体工商经营的就业人员，包括个体经营者和在个体工商户劳动的家庭帮工和雇工。

职工 指在国有、城镇集体、联营、股份制、外商和港、澳、台投资、其他单位及其附属机构工作，并由其支付工资的各类人员。不包括下列人员：(1)乡镇企业就业人员；(2)私营企业就业人员；(3)城镇个体劳动者；(4)离休、退休、退职人员；(5)再就业的离、退休人员；(6)民办教师；(7)在城镇单位中工作的外方及港、澳、台人员；(8)其他按有关规定不列入职工统计范围的人员。(1998 年及以后的数据均为在岗职工数据，其他相关指标如职工工资总额，职工平均工资等指标也从1998 年按此口径进行了相应调整)。

国有单位 指资产归国家所有的经济组织。包括按《中华人民共和国企业法人登记管理条例》规定登记注册的非公司制的经济组织，以及中央、地方各级国家机关、事业单位和社会团体。

集体单位 指生产资料归集体所有，并按《中华人民共和国企业法人登记管理条例》规定登记注册的经济组织。

其他单位包括股份合作单位、联营单位、有限责任公司、股份有限公司、港澳台商投资单位以及外商投资单位等其他登记注册类型单位。

在岗职工 指在本单位工作并由单位支付工资的人员，以及有工作岗位，但由于学习、病伤产假等原因暂未工作，仍由单位支付工资的人员。

工资总额 指各单位在一定时期内直接支付给本单位全部职工的劳动报酬总额。工资总额的计算原则应以直接支付给职工的全部劳动报酬为根据。各单位支付给职工的劳动报酬以及其他根据有关规定支付的工资，不论是计入成本的还是不计入成本的，不论是按国家规定列入计征奖金税项目的，还是未列入计征奖金税项目的，不论是以货币形式支付的还是以实物形式支付的，均包括在工资总额内。

平均工资 指企业、事业、机关单位的职工在一定时期内平均每人所得的货币工资额。它表明一定时期职工工资收入的高低程度，是反映职工工资水平的主要指标。计算公式为:

平均工资=报告期实际支付的全部职工工资总额 ×100%

报告期全部职工平均人数

平均工资指数 指报告期职工平均工资与基期职工平均工资的比率，是反映不同时期职工货币工资水平变动情况的相对数。计算公式为:

平均工资指数=报告期职工平均工资×100%

基期职工平均工资

平均实际工资指数职工平均实际工资指扣除物价变动因素后的职工平均工资。职工平均实际工资指数是反映实际工资变动情况的相对数，表明职工实际工资水平提高或降低的程度。计算公式为:

平均实际工资指数=	报告期职工平均工资指数	×100%

报告期城镇居民消费价格指数

城镇单位就业人员劳动报酬指各单位在一定时期内直接支付给本单位全部就业人员的劳动报酬总额。包括职工工资总额和其他就业人员劳动报酬总额。

平均劳动报酬指企业、事业、机关等单位的全部就业人员在一定时期内平均每人所得的货币工资额。

计算公式为：

报告期实际支付的全部

平均劳动报酬=	就业人员劳动报酬	×100%

报告期全部就业人员平均人数

城镇登记失业人员指有非农业户口，在一定的劳动年龄内（16 周岁至退休年龄），有劳动能力，无业而要求就业，并在当地就业服务机构进行求职登记的人员。

城镇登记失业率城镇登记失业人员与城镇单位就业人员（扣除使用的农村劳动力、聘用的离退休人员、港澳台及外方人员）、城镇单位中的不在岗职工、城镇私营业主、个体户主、城镇私营企业和个体就业人员、城镇登记失业人员之和的比。计算公式为：

城镇登记	城镇登记失业人数	×100%

失业率	=	(城镇单位就业人员-使用的农村劳动力-

聘用的离退休人员-聘用的港澳台及外方人员)+

不在岗职工+城镇私营业主+城镇个体户主+

城镇私营企业及个体就业人员+城镇登记失业人数

**Explanatory Notes on Principle Statistical Indicators**

**Total Population** refers to the total number of people alive at a certain point of time within a given area.The annual statistics on total population is taken at midnight, the 3lst of December, not including residents in Taiwan province, Hong Kong SAR and Macao SAR and Chinese national residing abroad.

**Urban Population and Rural Population** Urban population refers to all people residing in cities and towns,while rural population refers to population other than urban population.

**Population Density** refers to the ratio of population to the area at a certain point of time within a given area,i.e., the population of unit land area at a certain point of time, which is often expressed as number of inhabitants per square kilometer:

Population density = number of population in the region/land area in the region

**Birth Rate or (crude Birth Rate)** refers to the ratio of the number of births to the average population during a certain period of time (usually a year) which is often expressed in ‰. The formula is as follows:

Birth Rate = Number of Births/Annual Average Number of Population×1000‰.

Number of births refers to live births, i.e., the births when babies had showed any vital phenomena regardless of the length of pregnancy.

Annual Average Number of Population is the average of the number of population at the beginning of the year and that at the end of the year. Sometimes it is substituted for with the mid year population.

**Death Rate (or Crude Death Rate)** refers to the ratio of the number of deaths to the average population (or mid-period population) during a certain period of time (usually a year) which is often expressed in ‰. The formula is as follows:

Death Rate = Number of Deaths/Annual Average Number of Population×1000‰.

**Natural Growth Rate of Population** refers to the ratio of natural increase in population (number of births minus number of deaths) in a certain period of time (usually a year) to the average population (or mid-period population)of the same period which is often expressed in ‰. The formulas are as follows:

Natural Growth of Population = (Number of Birth－Number of Deaths)/Average Number of Population×1000‰.

Natural Growth Rate of Population = Birth Rate－Death Rate

**Sex Ratio** reflects the indicator of male population and female population, which refers to the ratio of male population to female population in total population or population by age group. It usually means relevant male population per 100 female populations. The formula is as follows:

Sex ratio = male population/female population ×100.

**Agricultural and Non-agricultural Population** is classified according to people’s residence registration nature recorded on their permanent residence booklets issued by public security organs.

**Economically Active Population** refers to the population aged 16 and over who are capable of working, are participating in or willing to participate in economic activities, including employed persons and unemployed persons.

**Employed Persons** refer to persons aged 16 and over who are engaged in gainful employment and thus receive remuneration payment or earn business income. This indicator reflects the actual utilization of total labour force during a certain period of time and is often used for the research on China’s economic situation and national power.

**Persons Employed in Various Units** refer to all the persons working in government agencies of various levels, political and party organizations, social organizations, enterprises and institutions, and receiving wages or other forms of payment. They include fully-employed staff and workers, re-employed retirees, teachers in the schools run by the local people, foreigners and Chinese compatriots from Hong Kong, Macao, and Taiwan working in various units, part-time employees, employees of other units working temporarily at current posts, and employees holding the second job, but do not include persons who have left their working units while keeping their labour contract (employment relation) unchanged. This indicator reflects the total number of laborers actually engaged in production or other operations in various units.

**Persons Employed in Private Enterprises and Self-Employed Individuals in Urban Areas Persons employed in private enterprises** refer to the persons employed in the private enterprises which have been registered at the departments of industrial and commercial administration for which the business operation are situated at a county town (i.e. a town where the county government is located), or at urban areas with administrative hierarchy higher than a county town. The self-employed individuals in urban areas refer to persons who hold the certificates of residence in urban areas or have resided in the urban areas for a long time and have been registered at the departments of industrial and commercial administration and approved to be engaged in individual industrial or commercial business, including self-employed persons as well as helpers and hired labourers who work in individual households.

**Staff and Workers** refer to persons working in, and receive payment from units of state ownership, collective ownership, joint ownership, share holding ownership, foreign ownership, and ownership by entrepreneurs from Hong Kong, Macao, and Taiwan, and other types of ownership and their affiliated units. They do not include 1)persons employed in township enterprises, 2) persons employed in private enterprises, 3) urban self-employed persons, 4) retirees, 5) re-employed retirees, 6) teachers in the schools run by the local people, 7) foreigners and persons from Hong Kong, Macao and Taiwan who work in urban units, and 8) other persons not to be included byrelevant regulations. (Data since 1998 refer to fully employed staff and workers. Other related statistics indicators,such as total wage bill and average wage are adjusted since 1998 accordingly).

**State-owned Units** refer to economic units whose assets are owned by the state, including non-corporation units registered according to Regulation of the People’s Republic of China on the Registration of Enterprises and Corporations, state organs, institutions and social organizations at the central-level and local levels.

**Collective-owned Units** refer to economic units registered according to Regulation of the People’s Republic of China on the Registration of Enterprises and Corporations where the means of production are collectively owned.

**Units of Other Types of Ownership** refer to units registered with other types of ownership, including cooperative units, joint ownership units, limited liability corporations, share holding corporations, units funded by entrepreneurs from Hong Kong, Macao, and Taiwan, and foreign- funded units.

**Employed Staff and Workers** refer to persons who work in, and receive wages from their working units,including persons who have their work posts but are temporarily absent from work for reasons of study or on sick,injury or maternal leave and still receive wages from their working units.

**Total Wage Bill** refers to the total remuneration payment to staff and workers in various units during a certain period of time. The calculation of total wage bill is based on the total remuneration payment to the staff and workers. Therefore, all the wages and salaries and other payments to staff and workers are included in the total wage bill regardless of sources, reckoning the cost of production or not, category, listing as items of premium taxation or not, and forms, paying in cash or in kind.**Average Wage** refers to the average wage in money terms per person during a certain period of time for staffand workers in enterprises, institutions, and government agencies, which reflects the general level of wage incomeduring a certain period of time and is calculated as follows:

Total Wage Bill of Staff and

Average Wage = Workers at Reference Time

Average Number of Staff and

Workers at Reference Time

**Average Wage Indices** refers to the ratio of average wage of staff and workers at the reference period to that at the base period, which reflects the change of wage of staff and workers at the different period. It is calculated as follows:

Average	Average Wage of Staff and

Wage Indices = Workers at Reference Time	×100%

Average Wage of Staff and

Workers at Base Period

**Average Real Wage Indices** average real wage of staff and workers refers to the average wage of staff and workers after removing the effects of the price changes and average real wage indices of staff and workers refers to the change of real wage, which reflects the relative increasing or decreasing level of real wage of staff and workers,which is calculated as follows:

Average Real	Average Wage Indices of Staff and

Wage Indices =	Workers at the Reference Time	×100%

Urban Consumer Price

Indices at Reference Time×100%

**Earning** refer to total remuneration payment to all employees in various units in urban areas (did not include urban private units and self-employed individuals) during a certain period of time, including staff and workers and other employees (i.e., reemployed retirees or those who are from Hong Kong, Macao, Taiwan province or other countries).

**Average Earning** refer to average earning level in money terms per employee in the enterprise, institution and government organ during a certain period of time, it is calculated as follows:

Average Earning	Total Earnings of Employees

Of Employees	=	at Reference Period

Average Number of

Employees at Reference Period

**Registered Unemployed Persons in Urban Areas** refer to the persons with non-agricultural household registration at certain working ages (16 years old to retirement age), who are capable of working, unemployed and willing to work, and have been registered at the local employment service agencies to apply for a job.

**Registered Unemployment Rate in Urban Areas** refers to the ratio of the number of the registered unemployed persons to the sum of the number of persons employed in various units (minus the employed rural labour force, re-employed retirees, and Hong Kong, Macao, Taiwan or foreign employees), laid-off staff and workers in urban units, owners of private enterprises in urban areas, owners of self-employed individuals in urban areas, employees of private enterprises in urban areas, employee of self-employed individuals in urban areas, and the registered unemployed persons in urban areas. The formula is as follows:

Registered	number of registered urban	×100%

unemployment =	unemployed persons

rate in urban areas	number of persons employed in urban units-

employed rural labour force unemployed persons

in urban areas urban self - employed individuals

registered of urban private enterprises employees

of urban self - employed individuals employees

urban private enterprises owners of laid - off staff

and workers owners of Macao, Taiwan or foreign

employees re - employed retirees - Hong Kong,
