# Supplementary material for: The measurement of competitiveness of forest green food industry in Yunnan Province
Source: PLoS One. 2021 Dec 10;16(12):e0261133. doi: 10.1371/journal.pone.0261133 (PMC8664197; doi:10.1371/journal.pone.0261133)
Supplement: S1 File — http://stats.yn.gov.cn/. (Z01) [file pone.0261133.s001.z01 › the Yunnan statistical yearbook (2016-2020)/2016/16/十六、资源与环境.doc]

主要统计指标解释

自然资源 指人类可以直接从自然界获得，并用于生产和生活的物质资源。自然资源一般可以分为可再生资源和非再生资源两大类。可再生资源指在较短时间内可以再生、可以循环利用的资源，包括土地资源、水资源、气候资源、生物资源和海洋资源等。非再生资源指在使用后不能再生的资源，包括矿产资源和地热能源。

土地资源 土地指陆地的表层部分，它主要由岩石、岩石的风化物和土壤构成。土地资源按利用类型可以分为农用地、建筑用地和未利用地。农用地包括耕地、园地、林地、牧草地和水面。建筑用地包括居民点及工矿用地、交通用地和水利设施用地。未利用地指农用地和建筑用地以外的土地，包括滩涂、荒漠、戈壁、冰川和石山等。

耕地面积 指经过开垦用以种植农作物并经常进行耕耘的土地面积。包括种有作物的土地面积、休闲地、新开荒地和抛荒未满三年的土地面积。

林业用地面积 指生长乔木、竹类、灌木、沿海红树林等林木的土地面积，包括有林地、灌木林、疏林地、未成林造林地、迹地、苗圃等。

牧草地面积 指牧区和农区用于放牧牲畜或割草，植被覆盖度在5%以上的草原、草坡、草山等面积。包括天然的和人工种植或改良的草地面积。

森林资源 指森林、林木、林地以及依托森林、林木、林地生存的野生动物、植物和微生物。林木指树木和竹子。森林指以乔木为主体的植物群落，是集生的乔木及与共同作用的植物、动物、微生物和土壤、气候等的总体。

森林面积 指由乔木树种构成，郁闭度0.2以上（含0.2）的林地或冠幅宽度10米以上的林带的面积，即有林地面积。森林面积包括天然起源和人工起源的针叶林面积、阔叶林面积、针阔混交林面积和竹林面积，不包括灌木林地面积和疏林地面积。

森林覆盖率 指一个国家或地区森林面积占土地总面积的百分比。森林覆盖率是反映森林资源的丰富程度和生态平衡状况的重要指标。在计算森林覆盖率时，森林面积包括郁闭度0.2以上的乔木林地面积和竹林地面积，国家特别规定的灌木林地面积、农田林网以及“四旁”(村旁、路旁、水旁、宅旁)林木的覆盖面积。计算公式为：

森林覆盖率	=	森林面积	×100%

土地总面积

活立木总蓄积量 指一定范围内土地上全部树木蓄积的总量，包括森林蓄积、疏林蓄积、散生木蓄积和“四旁”树蓄积。

森林蓄积量 指一定森林面积上存在着的林木树干部分的总材积。它是反映一个国家或地区森林资源总规模和水平的基本指标之一，也是反映森林资源的丰富程度、衡量森林生态环境优劣的重要依据。

气候 指地球与大气之间长期能量交换与质量交换所形成的一种自然环境状态，它是多种因素综合作用的结果。气候既是人类生活和生产的环境要素之一，又是供给人类生活和生产的重要资源。气温、降水、湿度等气象要素的多年平均值是用来描述一个地区气候状况的主要参数，而各种气象要素某年、某月的平均值（或总量）则可以反映出该时期天气气候状况的重要特征。

气温 指空气的温度，我国一般以摄氏度（℃）为单位表示。气象观测的温度表是放在离地面约1.5米处，通风良好的百叶箱里测量的，因此，通常说的气温指的是离地面1.5米处百叶箱中的温度。其统计计算方法为：

月平均气温是将全月各日的平均气温相加，除以该月的天数而得。

年平均气温是将12个月的月平均气温累加后除以12而得。

降水量 指从天空降落到地面的液态或固态（经融化后）水，未经蒸发、渗透、流失而在地面上积聚的深度。其统计计算方法为：

月降水量是将全月各日的降水量累加而得。

年降水量是将12个月的月降水量累加而得。

土地调查面积 指行政区域内的土地调查总面积，包括农用地、建设用地和未利用地。

农用地 指直接用于农业生产的土地，包括耕地、园地、林地、牧草地及其他农用地。

湿地 指天然或人工、长久或暂时性的沼泽地、泥炭地或水域地带，包括静止或流动、淡水、半咸水、咸水体，低潮时水深不超过6米的水域以及海岸地带地区的珊瑚滩和海草床、滩涂、红树林、河口、河流、淡水沼泽、沼泽森林、湖泊、盐沼及盐湖。

造林总面积 指报告期内在荒山、荒地、沙丘、退耕地等一切可以造林的土地上，采用人工播种、飞机播种、植苗造林、分植造林等方法新植成片乔木林和灌木林，经过检查验收符合《造林技术规程》要求的单位面积株数，并按《中华人民共和国森林法实施条例》规定，成活率达85%以上（含85%，年降雨量在400毫米以下且无浇灌条件的地区造林成活率达70%以上）的总面积。“四旁”植树如一侧在四行以上，连片面积0.066公顷（一亩）以上，应统计在造林面积内。造林面积通常按所有制（国有、国有集体合作、集体和个人）、造林方式（人工、飞机播种）、主要林种用途（用材林、经济林、防护林、薪炭林、特种用途林）分组进行统计。

人工造林 指在宜林荒山荒地、宜林沙荒地、无立木林地、疏林地和退耕地等其他宜林地上通过播种、植苗和分植来提高森林植被覆被率的技术措施。

飞机播种 通过飞机播种，为宜林荒山荒地、宜林沙荒地、其他宜林地、疏林地补充适量的种源，并辅以适当的人工措施，在自然力的作用下使其形成森林或灌草植被，提高森林植被覆被率的技术措施。

用材林 指以生产木材为主要目的的森林和林木，包括以生产竹材为主要目的的竹林。

经济林 指以生产果品，食用油料、饮料、调料，工业原料和药材为主要目的的林木。经济林是人们为了取得林木的果实、叶片、皮层、胶液等产品作为工业原料或者供食用所营造的林木，如油茶、油桐、核桃、樟树、花椒、茶、桑、果等。

防护林 指以防护为主要目的的森林、林木和灌木丛。包括水源涵养林，水土保持林，防风固沙林，农田、牧场防护林，护岸林，护路林等。

薪炭林 指以生产燃料为主要目的的林木。

特种用途林 指以国防、环境保护、科学实验等为主要目的的森林和林木。包括国防林、实验林、母树林、环境保护林、风景林，名胜古迹和革命纪念地的林木，自然保护区的森林。

水资源总量 指评价区内降水形成的地表和地下产水总量，即地表产流量与降水入渗补给地下水量之和，不包括过境水量。

地表水资源量 指评价区内河流、湖泊、冰川等地表水体中可以逐年更新的动态水量，即当地天然河川径流量。

地下水资源量 指评价区内降水和地表水对饱水岩土层的补给量，包括降水入渗补给量和河道、湖库、渠系、渠灌田间等地表水体的入渗补给量。

地表水与地下水资源重复量 指地表水和地下水相互转化的部分，即天然河川径流量中的地下水排泄量和地下水补给量中来源于地表水的入渗补给量。

供水总量 指各种水源工程为用户提供的包括输水损失在内的毛供水量之和，不包括海水直接利用量。

用水总量 指分配给各类用户的包括输水损失在内的毛用水量之和，不包括海水直接利用量。

农业用水 指农田灌溉用水、林果地灌溉用水、草地灌溉用水和鱼塘补水。

工业用水 指工矿企业在生产过程中用于制造、加工、冷却、空调、净化、洗涤等方面的用水，按新水取用量计，不包括企业内部的重复利用水量。

生活用水 包括城镇生活用水和农村生活用水。城镇生活用水由居民用水和公共用水（含第三产业及建筑业等用水）组成；农村生活用水除居民生活用水外，还包括牲畜用水在内。

生态用水 仅包括人为措施供给的城镇环境用水和部分河湖、湿地补水，而不包括降水、径流自然满足的水量。

工业废水排放量 指经过企业厂区所有排放口排到企业外部的工业废水量。包括生产废水、外排的直接冷却水、超标排放的矿井地下水和与工业废水混排的厂区生活污水，不包括外排的间接冷却水（清污不分流的间接冷却水应计算在内）。

工业废水排放达标量 指报告期内废水中各项污染物指标都达到国家或地方排放标准的外排工业废水量，包括未经处理外排达标的，经废水处理设施处理后达标排放的，以及经污水处理厂处理后达标排放的。

工业废水排放达标率 指工业废水排放达标量占工业废水排放量的百分率，计算公式为：

工业废水排放达标率 ＝ 工业废水排放达标量×100%

工业废水排放量

化学需氧量(COD) 指用化学氧化剂氧化水中有机污染物时所需的氧量。COD值越高，表示水中有机污染物污染越重。

生活污水中化学需氧量(COD)排放量 指城镇居民每年排放的生活污水中的COD的量。用人均系数法测算。测算公式为：

城镇生活污水	城镇生活污水中	市镇非

中排放量	=	COD产生系数	×	农业人口×365

工业废气排放量 指报告期内企业厂区内燃料燃烧和生产工艺过程中产生的各种排入大气的含有污染物的气体的总量，以标准状态(273K，101325Pa)计算。测算公式为：

工业废气	燃料燃烧过程	生产工艺过程

排放量	＝	中废气排放量+ 中废气排放量

二氧化硫排放量 指报告期内工业SO_2_排放量与生活SO_2_排放量之和。

工业SO_2_排放量 指报告期内企业在燃料燃烧和生产工艺过程中排入大气的SO_2_总量，计算公式为：

工业SO_2_	燃料燃烧过程	生产工艺过程

排放量	＝ 中废气排放量 + 中废气排放量

工业SO_2_排放达标量 指排入大气的达到排放标准的工业二氧化硫量。

工业SO_2_排放达标率 指工业SO_2_排放达标量占工业SO_2_排放量的百分率。计算公式为：

工业SO_2_	工业SO_2_排放达标量

排放达标率 ＝	工业SO_2_排放量	×100%

工业固体废物产生量 工业固体废物产生量 指报告期内企业在生产过程中产生的固体状、半固体状和高浓度液体状废弃物的总量，包括危险废物、冶炼废渣、粉煤灰、炉渣、煤矸石、尾矿、放射性废物和其他废物等；不包括矿山开采的剥离废石和掘进废石（煤矸石和呈酸性或碱性的废石除外）。酸性或碱性废石指采掘的废石其流经水、雨淋水的pH 值小于4 或pH 值大于10.5 者。

工业固体废物排放量 指报告期内企业将所产生的固体废物排到固体废物污染防治设施、场所以外的数量，不包括矿山开采的剥离废石和掘进废石（煤矸石和呈酸性或碱性的废石除外）。

工业固体废物综合利用量 指报告期内企业通过回收、加工、循环、交换等方式，从固体废物中提取或者使其转化为可以利用的资源、能源和其他原材料的固体废物量（包括当年利用往年的工业固体废物贮存量），如用作农业肥料、生产建筑材料、筑路等。综合利用量由原产生固体废物的单位统计。

工业固体废物综合利用率 指工业固体废物综合利用量占工业固体废物产生量,包括综合利用往年贮存量的百分率。计算公式为：

工业固体废物

综合利用量

工业固体废物 ＝ 工业固体废物产生量×100%

综合利用率	综合利用往年贮存量

工业固体废物贮存量 指报告期内企业以综合利用或处置为目的，将固体废物暂时贮存或堆存在专设的贮存设施或专设的集中堆存场所内的数量。专设的固体废物贮存场所或贮存设施必须有防扩散、防流失、防渗漏、防止污染大气、水体的措施。

工业固体废物处置量 指报告期内企业将固体废物焚烧或者最终置于符合环境保护规定要求的场所，并不再回取的工业固体废物量,包括当年处置往年的工业固体废物贮存量。处置方式有填埋（其中危险废物应安全填埋）、焚烧、专业贮存场(库)封场处理、深层灌注、回填矿井及海洋处置（经海洋管理部门同意投海处置）等。

“三废”综合利用产品产值 指报告期内利用“三废” 作为主要原料生产的产品价值（现行价）；已经销售或准备销售的应计算产品价值，留作生产自用的不应计算产品价值。

城区面积 城区面积包括：（1）街道办事处所辖地域；（2）城市公共设施、居住设施和市政公用设施等连接到的其他镇（乡）地域；（3）常住人口在3000 人以上独立的工矿区、开发区、科研单位、大专院校等特殊区域。

城市建成区面积 指城市行政区内实际已成片开发建设、市政公用设施和公共设施基本具备的区域。对于核心城市来说，它包括核心区域和多个分散区域；对于一城多镇的城市来说，它包括若干个连片开发起来的区域。一般是指建成区外轮廓线所能包括的地区，也就是这个城市实际建设用地所达到的范围。

城区人口 指划定的城区范围的人口数。按公安部门的户籍人口统计为准。

城市污水排放量 指城市生活污水、工业废水的排放总量，包括从排水管道和排水沟（渠）排出的污水量。

城市污水处理量 指城市污水处理厂和处理装置实际处理的污水量。包括物理处理量、生物处理量和化学处理量。

城市污水集中处理率 指城市污水处理厂处理的污水量与城市污水排放总量的比率。计算公式：

城市污水处理率 ＝ 城市污水处理厂污水处理量×100%

城市污水排放总量

人均公园绿地面积 指报告期末区域内城市人口平均每人拥有的公园绿地面积。人口数采用年底人口数。其中,公园绿地指城市中向公众开放的、以游憩为主要功能，有一定的游憩设施和服务设施，同时兼有健全生态、美化景观、防灾减灾等综合作用的绿化用地。计算公式为：

人均公园绿地面积 ＝ 公园绿地面积×100%

城市人口数

建成区绿化覆盖率 指报告期末建成区内绿化覆盖面积与建成区面积的比率。计算公式为：

建成区绿化覆盖率 ＝ 建成区内绿化覆盖面积×100%

建成区面积

其中，绿化覆盖面积指城市中的乔木、灌木、草坪等所有植被的垂直投影面积。包括公共绿地、居住区绿地、单位附属绿地、防护绿地、生产绿地、道路绿地、风景林地的绿化种植覆盖面积、屋顶绿化覆盖面积以及零散树木的覆盖面积。乔木树冠下重叠的灌木和草本植物不能重复计算。

**Explanatory Notes on Principle Statistical Indicators**

**Natural Resources** refer to material resources that could be obtained from the nature by human being and used for production and living. Natural resources in general can be classified as renewable resources and non-renewable resources. Renewable resources refer to resources that could be renewed and recycled during a relatively short period of time, including land resource, water resource, climate resource, biology resource and marine resource. Non-renewable resources include resources that could not be renewed, such as minerals and geothermal resource.

**Land Resource** Land refers to the surface of the earth, consisting of mainly rocks and its weathering and earth. Land resource can be classified, by its utilization, as land for agriculture, land for construction and unused land. Land for agriculture includes cultivated land, plantation land, forestland, grassland and waters. Land for construction includes land for residential purpose, for manufacturing and mining, for transportation and for water-conservancy projects. Unused land refers to land other than land for agriculture and construction, including beaches, deserts, Gobi, glaciers and rock mountains.

**Area of Cultivated Land** refers to area of land reclaimed for the regular cultivation of various farm crops, including crop-cover land, fallow, newly reclaimed land and land laid idle for less than 3 years.

**Area of Afforested Land** refers to area for land for trees bamboo, bushes and mangrove, including forest-covered land, bush-covered land, sparse forest land, land planned for a forestation and nurseries of young trees.

**Area of Pasture** refers to area of grassland, grass-slopes and grass-covered hills with a vegetation-covering rate of over 5% that are used for animal husbandry or harvesting of grass. It includes natural, cultivated and improved grassland areas.

**Forest Resource** refers to forests, trees, forestland and wild animals, plants and microorganism that live on forest and trees. Trees include trees and bamboo. Forest refers to the population of clusters of trees and other plants, animals and microorganism as well as the earth and climate that have interactions with the trees.

**Forest Area** refers to the area of forest where trees and bamboo grow with canopy density above 0.2, including land of natural woods and planted woods, but excluding bush land and thin forest land. It reflects the total areas of afforestation.

**Forest Coverage Rate** refers to the ratio of area of afforested land to total land area. It is a very important indicator that reflects the status of abundance of forest resource and balance of the ecosystem.Forest area includes the area of trees and bamboo grow with canopy density above 0.2, the area of shrubby tree according to regulations of the government, the area of forest land inside farm land and the area of trees planted by the side of villages, farm houses and along roads and rivers. The formula for calculating forest coverage rate is as follows:

Forestry coverage rate （%） = Area of Afforested Land × 100%

Area of Total Land

**Total Standing Stock Volume** refers to the total stock volume of trees growing in land, including trees in forest, trees in sparse forest, scattered trees and trees planted by the side of villages, farm houses and along roads and rivers.

**Stock Volume of Forest** refers to total stock volume of wood growing in forest area, which shows the total size and level of forest resources of a country or a region. It is also an important indicator illustrating the richness of forest resource and the status of forest ecological environment.

**Climate** refers to the natural environment status formed by the long-term exchange of energy and mass between the earth and the atmosphere, and is the result of interaction of many factors. Climate is both one of the environment factors and also the important resources for living and production activities of the human being. The average values across several years of meteorological factors such as temperature, rainfall and humidity are used as important parameters to describe the climate of a region, while the average values（or total values） of a given year or month of meteorological factors reflect the key characteristics of climate for the period of time.

**Temperature** refers to the air temperature. China uses centigrade as the unit. The thermometry used for weather observation is put in a breezy shutter, which is 1.5 meters high from the ground. Therefore, the commonly used temperature refers to the temperature in the breezy shutter 1.5 meters away from the ground. The calculation method is as follows:

Monthly average temperature is the summation of average daily temperature of one month divided by the actual days of that particular month.

Annual average temperature is the summation of monthly average of a year divided by 12 months. Volume of Precipitation refers to the deepness of liquid state or solid state （thawed） water falling from the sky to the ground that has not been evaporated, infiltrated or run off. The calculation method is as follows:

Monthly precipitation is the summation of daily precipitation of a month.

Annual precipitation is the summation of 12 months precipitation of a year.

**Land for Agriculture Use** refers to land directly used for agriculture production, including land for cultivation, gardening, forests, herbage and other agriculture activities.

**Wetlands** refer to marshland and peat bog, whether natural or man-made, permanent or temporary; water covered areas, whether stagnant or flowing, with fresh or semi-fresh or salty water that is less than 6 meters deep at low tide; as well as coral beach, weed beach, mud beach, mangrove, river outlet, rivers, fresh-water marshland, marshland forests, lakes, salty bog and salt lakes along the coastal areas.

**Total Area of A forestation** refers to the total area of land suitable for a forestation, including barren hills, idle land, sand dunes, “grain for green” land, on which acres of arbores or bushes are planted through manual planting, airplane planting, plant seedlings, etc. in accordance with the required density standards of the Technical Procedures of A forestation, and with a survival rate of over 85% in line with the Implementing Rules of the Forest Law of the People’s Republic of China （or a survival rate of 75% in areas with less that 400 mm of annual rainfall and without irrigation facilities）. Included in this category are trees planted alone the roadsides, riversides, or next to houses that occupy an area over 0.066 hectares, or where more than 4 lines of trees are planted. Total area of a forestation is further classified by ownership （state-owned, state-collective, collective or private）, by approach of planting （manual, airplane）, and by type of forests （timber, by-products, protection, fuel, special use, etc.）.

**Area of Man-made Forests** refer to the area of stable growing forests, planted manually or by airplanes, with a survival rate of 80% or higher of the designed number of trees per hectare, or with a canopy density of 0.20 - or above after 3-5 years of manual planting or 5-7 years of airplane planting.

Timber Forests refer to forests which are mainly for the production of timber, including bamboo groves planted to harvest bamboos.

**By-product Forests** refer to forests that mainly produce fruits, nuts, edible oil, beverages, indigents, raw materials and medicine materials. By-product forests are planted to harvest the fruits, leaves, bark or liquid of trees, and consume them as food or raw materials for the manufacturing industry, such as tea-oil trees, tung oil trees, walnut trees, camphor trees, tea bushes, mulberry trees, fruit trees, etc.

**Protection Forests** refer to forests, trees and bushes planted mainly for protection or preservation purpose, including water resource conservation forests, water and soil conservation forests, windbreak and dune-fixing forests, farmland and pasture protection forests, riverside protection forests, roadside protection forests, etc.

**Fuel Forests** refer to forests planted mainly for fuels.

**Forests for Special Purpose** refer to forests planted mainly for national defense, environment protection or scientific experiments, including national defense forests, experimental forests, mother-tree forests, environment protection forests, scenery forests, trees in historical or scenic spots, roadside in natural reserves.

**Total Water Resources** refers to total volume of water resources measured as run-off for surface water from rainfall and recharge for groundwater in a given area, excluding transit water.

**Surface Water Resources** refers to total renewable resources which exist in rivers, lakes, glaciers and other collectors from rainfall and are measured as run-off of rivers.

**Groundwater Resources** refers to replenishment of aquifers with rainfall and surface water.

**Duplicated Measurement between Surface Water and Groundwater** refers to mutual exchange between surface water and groundwater, i.e. run-off of rivers includes some depletion into groundwater while groundwater includes some replenishment from surface water.

**Water Supply** refers to gross water supply by supply systems from sources to consumers, including losses during distribution.

**Water Use** refers to gross water use distributed to users, including loss during transportation, broken down into use by agriculture, industry, living consumption and ecological protection.

**Water Use by Agriculture** includes uses of water by irrigation of farming fields and by forestry, animal husbandry and fishing. Water use by forestry, animal husbandry and fishery includes irrigation of forestry and orchards, irrigation of grassland and replenishment of fishing farms.

**Water Use by Industry** refers to new withdrawals of water, excluding reuse of water within enterprises.

**Water Use by Living Consumption** includes use of water for living consumption in both urban and rural areas. Urban water use by living consumption is composed of household use and public use （including services, commerce, restaurants, cargo transportation, posts, telecommunications and construction）. Rural water use by living consumption includes both households and animals.

**Water Use by Ecological Protection** includes replenishment of rivers and lakes and use for urban environment.

**Waste Water Discharged by Industry** refers to the volume of waste water discharged by industrial enterprises through all their outlets, including waste water from production process, directly cooled water, groundwater from mining wells which does not meet discharge standards and sewage from households mixed with waste water produced by industrial activities, but excluding indirectly cooled water discharged （It should be included if the discharge is not separated from waste water）.

**Industrial Waste Water Meeting Discharge Standards** refers to volume of industrial waste water discharge which, with or without treatment, reaches national or local standards with regard to all pollutants. **Ratio of Industrial Waste Water Meeting Discharge Standards** refers to percentage of industrial waste water meeting discharge standards over total industrial waste water discharge. It is calculated as:

Ratio of industrial waste water	industrial waste water

meeting discharge standards	= meeting discharge standards

total industrial waste

water discharge

**Chemical Oxygen Demand**（**COD**） refers to the amount of oxygen required when chemical oxidants are used to oxidize organic pollutants in water. A higher value of COD corresponds to more serious pollution by organic pollutants.

**Volume of Chemical Oxygen Demand** （**COD**） **Generated by Urban Non-industrial Waster Water** refers to chemical oxygen demand generated through the annual discharge of non-industrial waste water by urban households. It is estimated as: **Industrial Waste Air Emission** refers to the discharge into atmosphere of waste air containing pollutants generated from fuel burning and production processes in enterprises within a given period of time. It is calculated at standard status （273K, 101325Pa） as:

Volume of chemical oxygen

demand （cod） generated =	Coefficient of COD	×	urban	× 365

by urban non - industrial	generated through urban	non - agricultural

waster water	non -industrial waste water	population

**Industrial Waste Air Emission** refers to the discharge into atmosphere of waste air containing pollutants generated from fuel burning and production processes in enterprises within a given period of time. It is calculated at standard status （273K, 101325Pa） as:

Industrial waste = emission through +	emission through

air emission	fuel burning	production process

**SO_2_ Emission** is calculated on the basis of consumption of coal by households and industrial activities.

**SO_2_ Emission through Industrial Activities** refers to volume of sulphur dioxide emission from fuel burning and production process by enterprises during a given period of time. It is calculated as:

SO_2_ emission	SO_2_ emission from	SO_2_ emission from

through industrial =	fuel burning	+	production process

activities

**SO_2_ Emission through Industrial Activities Meeting Discharge Standards** refers to volume of sulphur dioxide emission from fuel burning and production process by enterprises during a given period of time meeting discharge standards

**Ratio of SORRR2RRR Emission through Industrial Activities Meeting Discharge Standards** refers to percentage of sulphur dioxide emission from fuel burning and production process by enterprises meeting discharge standards over total volume of sulphur dioxide emission from fuel burning and production process by enterprises during a given period of time.

It is estimated as:

Ratio of SO_2_ emission through industrial activities meeting discharge Standards= SO_2_ emission through industrial activities meeting discharge standards ÷ SORRR2RRR emission through industrial activities.

**Industrial Solid Wastes Produced** refers to total volume of solid, semi-solid and high concentration liquid residues produced by industrial enterprises from production process in a given period of time, including hazardous wastes, slag, coal ash, gangue, tailings, radioactive residues and other wastes, but excluding stones stripped or dug out in mining - gangue and acid or alkaline stones not included （a stone is acid or alkaline according to the pH value of the water being below 4 or above 10.5 when the stone is in, or soaked by water）.

**Industrial Solid Wastes Discharged** refers to the volume of industrial solid wastes discharged by producing enterprises to disposal facilities or to other sites. The wastes exclude stones stripped or dug from mining （gangue and acid or alkaline waste stones not included）.

**Industrial Solid Wastes Utilized** refers to volume of solid wastes from which useful materials can be extracted or which can be converted into usable resources, energy or other materials by means of reclamation, processing, recycling and exchange （including utilizing in the year the stocks of industrial solid wastes of the previous year）. Examples of such utilizations include fertilizers, building materials and road materials. The information shall be collected by the producing units of the wastes.

**Ratio of Utilization of Industrial Solid Wastes** refers to the percentage of industrial solid wastes utilized over industrial solid wastes produced （including stocks of the previous years）. It is calculated as:

Rate of utilization of	volume of industrial

industrial solid wastes =	solid wastes utilized	×100%

industrial solid

wastes produced+

stock of previous years

**Stock of Industrial Solid Wastes** refers to the volume of solid wastes placed in special facilities or special sites for purposes of utilization or disposal. The sites or facilities should take measures against dispersion, loss, seepage, and air and water contamination.

**Industrial Solid Wastes Disposed** refers to the quantity of industrial solid wastes which are burnt or placed ultimately in the sites meeting the requirements for environmental protection and not salvaged or recycled （including disposition in the year of those wastes of previous years）. The disposition includes landfill （Safe landfills should be conducted for hazardous wastes）, incineration, containment spaces, deep underground disposal, backfill in mining pits and disposal at sea.

**Output Value of Products Made from Waste Gas, Waste Water and Solid Wastes** refers to the current value of products with waste gas, waste water and solid wastes as main materials of production. Products sold and ready to sell shall be included while those produced for own use shall not be included.

**Urban Area** includes 1）Area under Sub-District Offices 2）The area of other towns（villages） connected by urban public infrastructure, accommodations and municipal public infrastructure. 3）Independent industrial and mining areas, development areas, scientific research institutions, institutes of higher education and such special areas with a permanent residents above 3000 persons.

**Urban Completed Areas** refers to the areas of the districts which have been developed aggregately and with municipal public infrastructure and public infrastructure. For the core cities, it includes the core areas and many scattered areas. For a city with many towns, it included several districts which were developed into one district. Generally, it refers to all districts which can be covered by the outline of the developed areas, which is the size of actual land for construction of the city.

**Urban District Population** refers to the population of the defined urban districts. It is based on the household register statistics of the public security departments.

**Urban Sewage Discharge** refers to volume of the domestic sewage and industrial sewage, which includes the sewage discharged from the drainage pipe and drainage channel（ditch）.

**Treatment Rate of Urban Sewage** refers to the percentage of sewage volume disposed by the sewage treatment plants over the urban sewage discharge volume. It is calculated as:

Treatment Rate of Urban Sewage= sewage volume disposed by the sewage treatment plants ÷ urban sewage discharge volume×100%.

**Per Capita Public Green Land Area** refers to the public green area enjoyed by every urban resident in the end of the report period. The number of the population is the population at the year-end. The public green land refers to the green spaces opening to the public with certain recreation and service facilities and whose main function is providing recreation areas, at the same time, their comprehensive functions include improving ecological environment, beautifying scenery and preventing disasters.

It is calculated as:

Per capita public green land area= public green area ÷ urban population×100%

**Ratio of Green Covered Area in Completed Area** refers to percentage of the green coverage in the urban completed area over the urban completed area in the end of the report period.

It is calculated as:

Ratio of Green Covered Area in Completed Area= green coverage in the urban completed area ÷ urban completed area×100%

In which, the green coverage refer to the vertical shadow of all vegetation such as trees, shrubs and lawn. It includes the green coverage, roof greenery coverage and scattered trees of the public green area, residential quarter green area, units attached green area, green area for environmental protection, green space attached to urban road and square and scenic forest land. The overlapping of the bushes and herbaceous plants under the trees crown can’t be calculated repeatedly.
