# Supplementary material for: The measurement of competitiveness of forest green food industry in Yunnan Province
Source: PLoS One. 2021 Dec 10;16(12):e0261133. doi: 10.1371/journal.pone.0261133 (PMC8664197; doi:10.1371/journal.pone.0261133)
Supplement: S1 File — http://stats.yn.gov.cn/. (Z01) [file pone.0261133.s001.z01 › the Yunnan statistical yearbook (2016-2020)/2016/18/指标解释.docx]

主要统计指标解释

乡村户数指长期（一年以上）居住在乡镇(不包括城关镇)行政管理区域内的住户，还包括居住在城关镇所辖行政村范围内的农村住户。户口不在本地而在本地居住一年及以上的住户也包括在本地农村住户内；有本地户口，但举家外出谋生一年以上的住户，无论是否保留承包耕地都不包括在本地农村住户范围内。不包括乡村地区内的国家机关、团体、国有学校、企业、事业单位的集体户。

乡村人口数指乡村地区常住居民户数中的常住人口数，即经常在家或在家居住6个月以上，而且经济和生活与本户连成一体的人口。外出从业人员在外居住时间虽然在6个月以上，但收入主要带回家中，经济与本户连成一体，仍视为家庭常住户口；在家居住，生活和本户连成一体的国家职工、退休人员也为家庭常住人口。但是现役军人、中专及以上（走读生除外）的在校学生、以及常年在外（不包括探亲、看病等）且已经有稳定的职业与居住场所的外出从业人员，不应当做家庭常住人口。

乡村从业人员指乡村人口中16岁以上实际参加生产经营活动并取得实物或货币收入的人员，既包括劳动年龄内经常参加劳动的人员，也包括超过劳动龄但经常参加劳动的人员。但不包括户口在家的在外学生、现役军人和丧失劳动能力的人，也不包括待业人员和家务劳动者。从业人员年龄为16岁以上。从业人员按从事主业时间最长（时间相同按收入）分为农业从业人员、工业从业人员、建筑业从业人员、交运仓储及邮政从业人员、信息传输、计算机服务和软件业、批发与零售业从业人员、住宿和餐饮业从业人员、其他行业从业人员。

Explanatory Notes on Principal Statistical Indicators

Village Householdsrefer to households which reside in the administrative region of townships and towns(excludes county towns) during a long time(more than one year) and also include the rural households reside in the administrative region of county towns. Households without local registrations but have resided in local for more than more year are included in the rural households.Households with local registrations but have left home and worked out of local places for more than more year are excluded from the rural households no matter their cultivated lands are kept. Village Households exclude collective household of state organs and groups, state owned schools,enterprises and public institutions in the rural areas.

Village Populationrefer to permanent resident population of permanent resident households in the rural areas,which also refers to the population that often stay at home or reside at home for more than 6 months and whose economies and lives are integrated into their households. Employees who have left home for more than 6 months but bring their major salaries home and economies are integrated into their households are regarded as the family permanent residence population. Family permanent residence population also include national staff and retirees who live at home and lives are integrated into their households. Active duty servicemen, students of technical secondary school and above(exclude day students) and employees who has acquire steady job and fixed dwelling places out of their local places(not include those who go to visit their relatives and see a doctor)are exclude from the family permanent residence population.

Village Employeesrefer to village population who are above 16 years old, having participated in the activities of production and operation actually and getting physical or monetary income, including persons who always take part in the labour within labor age and older than labor age.Students whose registered permanent residence are at home and studying out of local places,active duty servicemen, persons who have lost the ability to work, job seekers and house workers are excluded. Employees are above 16 years old, and can be divided into employees engaging in agriculture, employees engaging in industry, employees engaging in construction, employees engaging in transportation, storage and post, employees engaging in information transmission, employees engaging in computer services and software, employees engaging in wholesale and retail, employees engaging in accommodation and catering services and employees engaging in other trades.
