# Supplementary material for: The measurement of competitiveness of forest green food industry in Yunnan Province
Source: PLoS One. 2021 Dec 10;16(12):e0261133. doi: 10.1371/journal.pone.0261133 (PMC8664197; doi:10.1371/journal.pone.0261133)
Supplement: S1 File — http://stats.yn.gov.cn/. (Z01) [file pone.0261133.s001.z01 › the Yunnan statistical yearbook (2016-2020)/2016/2/二、国民经济核算.doc]

主要统计指标解释

生产总值 即原国内生产总值，指按市场价格计算的一个国家（或地区）所有常住单位在一定时期内生产活动的最终成果（国家称国内生产总值，各省区市称地区生产总值）。生产总值有三种表现形态，即价值形态、收入形态和产品形态。从价值形态看，它是所有常住单位在一定时期内生产的全部货物和服务价值超过同期投入的全部非固定资产货物和服务价值的差额，即所有常住单位的增加值之和；从收入形态看，它是所有常住单位在一定时期内创造并分配给常住单位和非常住单位的初次收入之和；从产品形态看，它是所有常住单位在一定时期内最终使用的货物和服务价值与货物和服务净出口价值之和。在实际核算中，生产总值有三种计算方法，即生产法、收入法和支出法。

生产法 是从生产的角度衡量常住单位在核算期内新创造价值的一种计算方法。即从生产的全部货物和服务总产品的价值中，扣除生产过程中投入的中间产品的价值，得到增加值。全社会所有常住单位增加值的总和就是生产总值。计算公式为：

增加值=总产出-中间投入

国（地区）外净要素收入 是指本国（或本地区）居民对国（或本地区）外从事投资和提供劳务所得的要素收入，与外国（或本地区）居民对本国（或本地区）从事投资和提供劳务所得的要素收入的差额。

三次产业 三次产业的划分是世界上较为常用的产业结构分类，但各国的划分不尽一致。中国的三次产业划分是：

第一产业：是指农（种植）、林、牧、渔业（不含农、林、牧、渔服务业）。

第二产业：是指采矿业（不含开采辅助活动），制造业（不含金属制品、机械和设备修理业），电力、热力、燃气及水生产和供应业，建筑业。

第三产业即服务业，是指除第一产业、第二产业以外的其他行业。

总产出 指常住单位在核算期内生产的货物和服务的价值总和，总产出中既包括核算期内新增加的价值，也包括中间投入的转移价值，它反映了国民经济各个部门生产活动的总规模。

中间投入 指常住单位在生产货物或提供服务的过程中，消耗和使用的所有原材料、燃料动力等货物和各种服务的价值。中间投入也称中间消耗，货物投入是生产过程中消耗或转换的有形的物质产品，不包括固定资产。服务投入是在生产过程中消耗的各种服务，包括金融保险、运输邮电、文化教育等等。

劳动者报酬 指劳动者因从事生产活动所获得的全部报酬。包括劳动者获得的各种形式的工资、奖金和津贴，既包括货币形式的，也包括实物形式的，还包括劳动者所享受的公费医疗和医药卫生费、上下班交通补贴、单位支付的社会保险费、住房公积金等。对于个体经济来说，其所有者所获得的劳动报酬和经营利润不易区分，这两部分统一作为劳动者报酬处理。

生产税净额 指生产税减生产补贴后的余额。生产税指政府对生产单位从事生产、销售和经营活动以及因从事生产活动使用某些生产要素（如固定资产、土地、劳动力）所征收的各种税、附加费和规费。生产补贴与生产税相反，指政府对生产单位的单方面转移支出，因此视为负生产税，包括政策亏损补贴、价格补贴等。

固定资产折旧 指一定时期内为弥补固定资产损耗按照规定的固定资产折旧率提取的固定资产折旧，或按国民经济核算统一规定的折旧率虚拟计算的固定资产折旧。它反映了固定资产在当期生产中的转移价值。各类企业和企业化管理的事业单位的固定资产折旧是指实际计提的折旧费；不计提折旧的政府机关、非企业化管理的事业单位和居民住房的固定资产折旧是按照统一规定的折旧率和固定资产原值计算的虚拟折旧。原则上，固定资产折旧应按固定资产当期的重置价值计算，但是目前我国尚不具备对全社会固定资产进行重估价的基础，所以暂时只能采用上述办法。

营业盈余 指常住单位创造的增加值扣除劳动者报酬、生产税净额和固定资产折旧后的余额。它相当于企业的营业利润加上生产补贴，但要扣除从利润中开支的工资和福利等。

支出法地区生产总值 是从最终使用的角度反映一个国家或地区一定时期内生产活动最终成果的一种方法，包括最终消费支出、资本形成总额及货物和服务净出口三部分。计算公式为：

支出法地区生产总值=最终消费支出+资本形成总额+货物和服务净出口

最终消费支出 指常住单位为满足物质、文化和精神生活的需要，从本国经济领土和国外购买的货物和服务的支出。它不包括非常住单位在本国经济领土内的消费支出。最终消费支出分为居民消费支出和政府消费支出。

居民消费支出 指常住住户在一定时期内对于货物和服务的全部最终消费支出。居民消费支出除了直接以货币形式购买的货物和服务的消费支出外，还包括以其他方式获得的货物和服务的消费支出，即所谓的虚拟消费支出。居民虚拟消费支出包括如下几种类型：单位以实物报酬及实物转移的形式提供给劳动者的货物和服务；住户生产并由本住户消费了的货物和服务，其中的服务仅指住户的自有住房服务和付酬的家庭雇员提供的家庭和个人服务；金融机构提供的金融媒介服务。

政府消费支出 指政府部门为全社会提供的公共服务的消费支出和免费或以较低的价格向居民住户提供的货物和服务的净支出，前者等于政府服务的产出价值减去政府单位所获得的经营收入的价值，后者等于政府部门免费或以较低价格向居民住户提供的货物和服务的市场价值减去向住户收取的价值。

资本形成总额 指常住单位在一定时期内获得减去处置的固定资产和存货的净额，包括固定资本形成总额和存货增加两部分。

固定资本形成总额 指常住单位在一定时期内获得的固定资产减处置的固定资产的价值总额。固定资产是通过生产活动生产出来的，且其使用年限在一年以上、单位价值在规定标准以上的资产，不包括自然资产。可分为有形固定资本形成总额和无形固定资本形成总额。有形固定资本形成总额包括一定时期内完成的建筑工程、安装工程和设备工器具购置（减处置）价值，以及土地改良、新增役、种、奶、毛、娱乐用牲畜和新增经济林木价值。无形固定资本形成总额包括矿藏的勘探、计算机软件等获得减处置。

存货变动 指常住单位在一定时期内存货实物量变动的市场价值，即期末价值减期初价值的差额，再扣除当期由于价格变动而产生的持有收益。存货变动可以是正值，也可以是负值，正值表示存货上升，负值表示存货下降。存货包括生产单位购进的原材料、燃料和储备物资等存货，以及生产单位生产的产成品、在制品和半成品等存货。

货物和服务净出口 指货物和服务出口减货物和服务进口的差额。出口包括常住单位向非常住单位出售或无偿转让的各种货物和服务的价值；进口包括常住单位从非常住单位购买或无偿得到的各种货物和服务的价值。由于服务活动的提供与使用同时发生，一般把常住单位从非常住单位得到的服务作为进口，非常住单位从常住单位得到的服务作为出口。货物的出口和进口都按离岸价格计算。

**Explanatory Notes on Principal Statistical Indicators**

**Gross Regional Product** was called gross domestic Product under national level before. At national level,it is still called gross domestic product , It refers to the final products at market prices produced by all resident entities in a country (or territory) during a certain period of time. Gross regional product is expressed in three different forms, i.e. value, income, and product respectively. Gross regional product in its value form refers to the difference between the total value of all goods and services produced by all resident entities during a certain period of time and that of goods and services of the nature of non-fixed assets input in the same period, i.e. it is the sum of added value of all resident entities. Gross regional product in the form of income refers to the total initial income created by all resident entities and distributed to resident and non-resident entities in a certain period of time. Gross regional product in the form of product refers to the balance of the value of all goods and services for final consumption by all resident units minus the net export value of goods and services during a given period of time. In the practice of national accounting, gross regional product is calculated by three approaches, i.e. production approach, income approach and expenditure approach.

**Production Approach** refers to the calculation method for measuring the newly increased value of resident entities in the accounting period from the perspective of production, i.e. the value of total goods and services produced minus that of intermediate products input in the process of production is added value. The sum of added value created by all resident entities makes GDP. The formula is as follows: Added Value = Total Output –Intermediate Input

**Net Factor Income from Abroad** refers to the difference between factor income earned by residents of a country (or territory) from their investment and labor services in foreign countries (or territories) and that earned by residents of foreign countries (or territories) from their investment and labor services in that country (or territory).

**Three Industries** Classification of economic activities into three industries is a common practice in the world,although the grouping varies to some extent form country to country. In China, economic activities are categorized as follows:

Primary industry refers to agriculture(planting) , forestry, animal husbandry and fishery industries (not including services in support of agriculture, forestry, animal husbandry and fishery industries).

Secondary industry refers to mining and quarrying(not including support activities for mining), manufacturing(not including repair service of metal products, machinery and equipment), production and supply of electricity, heat, gas and water, and construction.

Tertiary industry refers to all other economic activities not included in the primary or secondary industries.

**Total Output** refers to the total value of goods and services produced by resident entities in the accounting period, including newly increased value and transfer value in intermediate input, which reflects the general scale of productive activities of each sector of the national economy.

**Intermediate Input** also known as intermediate consumption refers to the value of goods such as raw materials, fuels, power, etc. and various services consumed and used in the process of production or provision of services. Goods input refers to tangible material products consumed or transferred in production; and services input are those consumed in production, including banking, insurance, communications and transportation, culture,education, etc.

**Compensation of Employees** refers to the total payment of various forms to employees for the productive activities they are engaged in. It includes wages, bonuses and allowances, which the employees earn in cash or in kind. It also includes the free medical services provided to the employees and the medicine expenses, transport subsidies and social insurance, and housing fund paid by the employers. As regards the individual economy, since compensation of employees is not easily distinguishable from the operating surplus, both parts are treated as compensation of employees.

**Net Taxes on Production** refers to taxes on production less subsidies on production. The taxes on production refers to the various taxes, extra charges and fees levied on the production units on their production, sale and business activities as well as on the use of some factors of production, such as fixed assets, land and labour in the production activities they are engaged in. In contrast to taxes on production, subsidies on production refer to the unilateral government transfer to the production units and are therefore regarded as negative taxes on production.They include subsidies on the loss due to implementation of government policies, price subsidies, etc.

**Depreciation of Fixed Assets** refers to the depreciation of fixed assets in a given period, drawn in accordance with the stipulated depreciation rate for the purpose of compensating the wear-and-tear loss of the fixed assets or the depreciation of fixed assets imputed in accordance with the stipulated unified depreciation rate in the national economic accounting system. It reflects the value of transfer of the fixed assets in the production of the current period. The depreciation of fixed assets in various enterprises and institutions managed as enterprises refers to the depreciation expenses actually drawn. In government agencies and institutions not managed as enterprises which do not draw the depreciation expenses, as well as for the houses of residents, the depreciation of fixed assets is the imputed depreciation, which is calculated in accordance with the stipulated unified depreciation rate. In principle,the depreciation of fixed assets should be calculated on the basis of the re-purchased value of the fixed assets.However, currently the conditions in China do not facilitate the revaluation of all the fixed assets. Therefore, only the above-mentioned methods can be adopted at present.

**Operating Surplus** refers to the balance of the value added created by the resident units after deducting the labourers remuneration, net taxes on production and the depreciation of fixed assets. It is equivalent to the business profit of the enterprises plus subsidies to production, but the wages and welfare expenses paid from the profits should be deducted.

**Gross Regional Product by Expenditure Approach** refers to the method of measuring the final results of production activities of a country or region during a given period from the perspective of final uses. It includes final consumption expenditure, gross capital formation and net export of goods and services. The formula for computation is.:

Gross Regional Product by expenditure approach = final consumption expenditure + gross capital formation +net export of goods and services

**Final Consumption Expenditure** refers to the total expenditure of resident units for purchases of goods and services from both the domestic economic territory and abroad to meet the needs of material, cultural and spiritual life. It does not include the expenditure of non-resident units on consumption in the economic territory of the country. The final consumption expenditure is broken down into household consumption expenditure and government consumption expenditure.

**Household Consumption Expenditure** refers to the total expenditure of resident households on the final consumption of goods and services. In addition to the consumption of goods and services bought by the households directly with money, the household consumption expenditure also includes expenditure on goods and services obtained by the households in other ways, i.e. the so-called imputed consumption expenditure, which includes the following: a) the goods and services provided to households by employers in the form of payment in kind and transfer in kind; b) goods and services produced and consumed by the households themselves, in which the services refer to the owner-occupied housing and services offered by payed family employees; c) financial intermediate services provided by financial institution.

**Government Consumption Expenditure** refers to the consumption expenditure spent for the provision of 40 National Accounts public services provided by the government to the whole country and the net expenditure on the goods and services provided by the government to households free of charge or at reduced prices. The former equals to the output value of the government services minus the value of operating income obtained by the government departments.The latter equals to the market value of the goods and services provided by the government free of charge or at reduced prices to the households minus the value received by the government from the households.

**Gross Capital Formation** refers to the fixed assets acquired less disposals and the net value of inventory, thus including gross fixed capital formation and changes in inventories.Gross Fixed Capital Formation refers to the value of acquisitions less those disposals of fixed assets during a given period. Fixed assets are the assets produced through production activities with unit value above a specified amount and which could be used for over one year. Natural assets are not included.Gross fixed capital formation can be categorized into total tangible fixed capital formation and total intangible fixed capital formation. Total tangible fixed capital formation includes the value of the construction projects and installation projects completed and the equipment, apparatus and instruments purchased (less those disposed) as well as the value of land improved,the value of draught animals, breeding stock and animals for milk, for wool and for recreational purposes and the newly increased forest with economic value. Total intangible fixed capital formation includes the prospecting of minerals and the acquisition of computer software minus the disposal of them.

**Changes in Inventories** refers to the market value of the change in the physical volume of inventory of resident units during a given period, i.e. the difference between the values at the beginning and at the end of the period minus the gains due to the change in prices. The changes in inventories can have a positive or a negative value. A positive value indicates an increase in inventory while a negative value indicates a decrease in inventory.The inventory includes raw materials, fuels and reserve materials purchased by the production units as well as the inventory of finished products, semi-finished products and work-in-progress.

**Net Export of Goods and Services** refers to the exports of goods and services subtracting the imports of goods and services. Exports include the value of various goods and services sold or gratuitously transferred by resident units to non-resident units. Imports include the value of various goods and services purchased or gratuitously acquired resident units from non-resident units. Because the provision of services and the use of them happen simultaneously, the acquisition of services by resident units from abroad is usually treated as import while the acquisition of services by non-resident units in this country is usually treated as export. The exports and imports of goods are calculated at FOB.
