# Supplementary material for: The measurement of competitiveness of forest green food industry in Yunnan Province
Source: PLoS One. 2021 Dec 10;16(12):e0261133. doi: 10.1371/journal.pone.0261133 (PMC8664197; doi:10.1371/journal.pone.0261133)
Supplement: S1 File — http://stats.yn.gov.cn/. (Z01) [file pone.0261133.s001.z01 › the Yunnan statistical yearbook (2016-2020)/2016/3/三、固定资产投资.doc]

主要统计指标解释

固定资产投资 是以货币形式表现的在一定时期内建造和购置固定资产的工作量以及与此有关的费用的总称。该指标是反映固定资产投资规模、结构和发展速度的综合性指标,又是观察工程进度和考核投资效果的重要依据。固定资产投资按登记注册类型可分为国有、集体、个体、联营、股份制、外商、港澳台商、其他等。固定资产投资统计口径为各种登记注册类型的企业、事业、行政单位及个体户进行的计划总投资（或实际需要总投资）500万元以上的建设项目投资。

房地产开发投资 指各种登记注册类型的房地产开发公司、商品房建设公司及其他房地产开发法人单位和附属于其他法人单位实际从事房地产开发或经营活动的单位统一开发的包括统代建、拆迁还建的住宅、厂房、仓库、饭店、宾馆、度假村、写字楼、办公楼等房屋建筑物和配套的服务设施，土地开发工程（如道路、给水、排水、供电、供热、通讯、平整场地等基础设施工程）的投资；不包括单纯的土地交易活动。

固定资产投资按国民经济行业分 根据建设项目建成投产后的主要产品或主要用途及社会经济活动性质来确定国民经济行业。一般情况下，一个建设项目或一个企业、事业单位只能属于一种国民经济行业。

固定资产投资按隶属关系分 是按建设单位或企业、事业、行政单位的主管上级机关确定的。

（1）中央：是指中共中央、人大常委会和国务院各部、委、局、总公司以及直属机构直接领导的建设项目和企业、事业、行政单位。这些单位的固定资产投资计划由国务院各部门直接编制和下达，建设中所需物资、主要设备以及建设中的问题都由中央有关部门安排和解决。

（2）地方：是由省（自治区、直辖市）、地区（州、盟、省辖市）、县（旗、县级市）三级政府及业务主管部门直接领导和管理的建设项目、企业、事业、行政单位。地方项目还包括不隶属以上各级政府及主管部门的建设项目和企业、事业单位，如外商投资企业和无主管部门的企业等。

固定资产投资按建设性质分 根据整个建设项目情况来确定。建设项目的性质一般分为新建、扩建、改建和技术改造、迁建、恢复等。

（1）新建：一般指从无到有开始建设的企业、事业和行政单位或建设项目。现有企业、事业、行政单位一般不属于新建。但如有的单位原有基础很小，经过建设后新增的固定资产价值超过该企、事业、行政单位原有固定资产价值（原值）三倍以上的也应作为新建。

（2）扩建：指在厂内或其他地点，为扩大原有产品的生产能力（或效益）或增加新的产品生产能力，而增建主要的生产车间（或主要工程）、分厂、独立的生产线。行政、事业单位在原单位增建业务用房（如学校增建教学用房、医院增建门诊部、病房等）也作为扩建。

现有企、事业单位为扩大原有主要产品生产能力或增加新的产品生产能力，增建一个或几个主要生产车间（或主要工程）、分厂，同时进行一些更新改造工程的，也应作为扩建。

（3）改建和技术改造：指现有企业、事业单位，对原有设施进行技术改造或更新（包括相应配套的辅助性生产、生活福利设施） 的建设项目。现有企业、事业单位为适应市场变化的需要，而改变企业的主要产品种类（如军工企业转产民用品等） 的建设项目，应作为改建。原有产品生产作业线由于各工序（车间）之间能力不平衡，为填平补齐充分发挥原有生产能力而增建不增加本企业主要产品设计能力的车间，也应作为改建。技术改造是指企业、事业单位在现有基础上，用先进的技术代替落后的技术，用先进的工艺和装备代替落后的工艺和装备，以改变企业落后的技术经济面貌，实现以内涵为主的扩大再生产，达到提高产品质量、促进产品更新换代、节约能源、降低消耗、扩大生产规模、全面提高社会经济效益的目的。技术改造具体包括以下内容：机器设备和工具的更新改造；生产工艺改革、节约能源和原材料的改造；厂房建筑和公共设施的改造；保护环境进行的“三废”治理改造；劳动条件和生产环境的改造等。

固定资产投资按构成分 固定资产投资活动按其工作内容和实现方式分为建筑工程，安装工程，设备、工具、器具购置，其他费用四个部分。

（1）建筑工程：指各种房屋、建筑物的建造工程，又称建筑工作量。包括各种房屋建造工程；各种用途设备基础和各种工业窑炉的砌筑工程及金属结构工程；为施工而进行的各种准备工作和临时工程以及完工后的清理工作等；铁路、道路的铺设，矿井的开凿及石油管道的架设等；水利工程；防空地下建筑等特殊工程；列入房屋工程预算内的暖气、卫生、通风、照明、煤气等设备的价值及装设油饰工程；列入建筑工程预算内的各种管道（蒸汽、压缩空气、石油、给排水等管道）、电力、电讯电缆导线等的敷设工程；房地产开发单位进行的商品房屋开发建设工程、土地开发工程。

（2）安装工程：指各种设备、装置的安装工程，又称安装工作量。包括各种需要安装设备的装配和安装，与设备相连的装设工程及附属于被安装设备的其他工程；为测定安装工程质量，对设备进行的试运工作。但不包括被安装设备本身的价值。

（3）设备、工具、器具购置：指建设单位或企、事业单位购置或自制的，达到固定资产标准的设备、工具、器具的价值。新建单位及扩建单位的新建车间，按照设计或计划要求购置或自制的全部设备、工具、器具，不论是否达到固定资产标准均计入“设备、工具、器具购置”中。

（4）其他费用：指在固定资产建造和购置过程中发生的，除上述几项内容以外的各种应分摊计入固定资产的费用。

新增固定资产 指已经完成建造和购置过程，并已交付生产或使用单位的固定资产的价值。包括已经建成投入生产或交付使用的工程投资和达到固定资产标准的设备、工具、器具的投资及有关应摊入的费用。属于增加固定资产价值的其他建设费用，应随同交付使用的工程一并计入新增固定资产。

施工房屋面积 指报告期内施工的全部房屋建筑面积。包括本期新开工的面积和上期开工跨入本期继续施工的房屋面积，以及上期已停缓建在本期复工的房屋面积。本期竣工和本期施工后又停缓建的房屋，其建筑面积仍计入本期施工房屋面积中。

房屋新开工面积 指报告期内新开工的全部房屋建筑面积，以单位工程为核算对象。房屋新开工面积指整栋房屋的全部建筑面积，不能分割计算。

竣工房屋面积 指在报告期内房屋建筑按照设计要求已全部完工，达到住人和使用条件，经验收鉴定合格（或达到竣工验收标准），可正式移交使用的各栋房屋建筑面积的总和。

竣工房屋价值 指在报告期内竣工房屋本身的建造价值。竣工房屋价值按房屋设计和预算规定的内容计算。

施工项目 指报告期内进行过建筑或安装施工活动的项目。凡是报告期内施过工的建设项目，不论施工时间长短，均作为施工项目统计。施工项目个数可以反映一定时期固定资产投资的实际规模，与同期全部建成投产项目个数相比，可以从建设速度的角度反映固定资产投资的效果。

全部建成投产项目 报告期内按设计文件规定建成主体工程和相应配套的辅助设施，形成生产能力或工程效益，经验收合格，并且已正式投入生产或交付使用的建设项目。

新增生产能力 指通过固定资产投资活动而增加的设计能力或工程效益,它是用实物形态表示的固定资产投资的成果。新增生产能力的计算,是以能独立发挥生产能力或效益的工程为对象。当工程建成,经有关部门鉴定合格,正式移交投入生产,即可计算新增生产能力。

规划用地面积 指根据经有关部门批准的项目规划，建设项目需要使用的土地面积。

实际征用和购置土地面积 指报告期内通过征用等各种方式获得使用权的土地面积。

实际征用和购置土地成交价款 指报告期内征用和购置土地进行土地使用权交易活动的最终金额。征用和购置的土地成交价款与征用和购置土地面积同口径，目的是正确计算平均土地征用和购置价格。

固定资产投资的资金来源 根据固定资产投资的资金来源不同,分为上年末结余资金和本年资金来源。其中本年资金来源又分为六种：

（1）国家预算资金包括中央预算资金和地方预算资金。其中中央预算资金分为财政拨款和财政安排的贷款两部分。

（2）国内贷款指报告期固定资产投资项目单位向银行及非银行金融机构借入的用于固定资产投资的各种国内借款。包括银行利用自有资金及吸收的存款发放的贷款、上级主管部门拨入的国内贷款、国家专项贷款（包括煤代油贷款、劳改煤矿专项贷款等），地方财政专项资金安排的贷款、国内储备贷款、周转贷款等。银行贷款、非银行金融机构贷款等。

（3）债券（是企业（公司）或金融机构通过发行各种债券筹集到的用于固定资产投资的资金。包括由银行代理国家专业投资公司发行的重点企业债券和基本建设债券。指企业或金融机构为筹集用于固定资产投资的资金向投资者出具的承诺按一定发行条件还本付息的债务凭证，包括金融债券和债券。

（4）利用外资指报告期收到的用于固定资产建造和购置投资的境外资金（包括设备、材料、技术在内）。计算利用外资时，需要折算成人民币，折算中所使用的外汇汇率按现汇计算，即按使用外汇时的汇率计算。

（5）自筹资金指固定资产投资单位报告期收到的，由各地区、各部门及企业、事业单位筹集用于固定资产投资的预算外资金，包括中央各部门、各级地方和企业、事业单位的自有资金。

（6）其他资金来源指在报告期收到的除以上各种资金之外其他用于固定资产投资的资金。包括社会集资、个人资金、无偿捐赠的资金及其他单位拨入的资金等。

**Explanatory Notes on Principal Statistical Indicators**

**Total Investment in Fixed Assets** refers to the volume of activities in construction and purchases of fixed assets and related fees, expressed in monetary terms during the reference period. It is a comprehensive indicator which shows the size, structure and growth of the investment in fixed assets, providing a basis for observing the progress of construction projects and evaluating results of investment. Total investment in fixed assets includes, by type of ownership, the investment by State-owned units, collective-owned units, individuals, joint ownership units, share-holding units, as well as investments by entrepreneurs from foreign countries and from Hong Kong, Macao and Taiwan, and by other units. Its statistical range is construction projects involving a total planned （or required）investment over 5 million yuan by enterprises of various types of ownership, institutions, administrative units and individuals in urban areas. In other words, all investments that take place in county towns and urban areas, investment in construction projects under the direct leadership and management of government agencies at and above county levels and investments by enterprises and institutions at and above county levels are covered in urban investment in fixed assets.

**Investment in Real Estate Development** refers to investment by real estate development companies, commercialized buildings construction companies and other real estate development units of various types of ownership in the construction of buildings, such as residential buildings, factory buildings, warehouses, hotels, guesthouses, holiday villages, office buildings, and the complementary service facilities and land development projects, such as roads, water supply, water drainage, power supply, heating supply, telecommunications, land leveling and other infrastructural projects. It does not include activities in pure land transactions.

**Investment in Fixed Assets by Sector** The classification of construction projects by sector is determined by the major products or the purpose of the projects when they are put into production or use, and by the nature of their social economic activities. In general, one project or one enterprise or institution can only be classified into one sector.

Investment in Fixed Assets by Jurisdiction of Managementrefers to the classification of investment by the competent authorities under which investment is made by construction units, enterprises, institutions or administrative units.

（1）**Central investment** refers to the investment in projects or by enterprises, institutions or administrative units which are under the direct leadership and management of the State Council and of the national commissions, ministries, agencies and State-owned large corporations. Various ministries and departments of the State Council prepare and implement plans for investment in fixed assets by those departments, and arrange and ensure the supply of materials and key equipment required for the projects.

（2）**Local investment** refers to the investment in projects or by enterprises, institutions or administrative units which are under the direct leadership and management of departments under the provincial, prefecture and county governments. Also included are projects by foreign-invested enterprises and enterprises without competent managing authorities.

**Investment in Fixed Assets by Type of Construction** Construction projects in general can be classified, by the type of construction, into new construction, expansion, reconstruction and technical transformation, moving and restoration.

（1）**New construction in General** refers to construction projects, which start from scratch, of enterprises, institutions, administrative agencies. Construction in existing enterprises, institutions or agencies is generally not considered as new construction. In case the size of the existing unit is quite small, and the value of newly added fixed assets is more than three times of the original value, the expansion will be considered as new construction.

（2）**Expansion** refers to construction of new major production workshop, branch factory or independent production line within a factory or in other locations, for the purpose of increasing the production capacity （or improving efficiency） or adding new production capacity. Newly constructed accommodation for the operation of institutions and administrative organizations（such as newly constructed buildings for teaching in schools, buildings for clinics or wards in hospitals, etc.）are also classified as expansion.

Also included in expansion are investments by existing enterprises or institutions in building major production line（s）or branch factory（ies）along with some work on innovation, for the purpose of expanding the production capacity of original products or producing new products.

（3）**Reconstruction and technical transformation** refers to construction projects by existing enterprises or institutions in innovation or technical transformation of the old facilities（including auxiliary production equipment and welfare facilities）. Also considered as reconstruction is the construction of new workshops by the existing enterprises or institutions to change the variety of products to meet the market demand （such as the production of civil products by defense industries）, or to bring the designed production capacity into full play through a more balanced production process on production lines. Technical transformation refers to replacement of old technology or equipment by new technology or equipment, in order to expand the reproduction through improvement of technology contents in production, to improve product quality, to promote new products, to save energy, to reduce consumption, to expand the production scale and to improve overall social-economic efficiency. Contents of technical transformation include: updating of machinery, equipment and tools; reforming production process by using energy or materials saving technology; construction of factory workshops and transformation of public facilities; improvement of working conditions and environment, etc.

**Investment in Fixed Assets by Structure** By their contents and the mode of implementation, investment activities are classified into 3 categories, i.e. construction and installation, purchase of equipment and instrument, and other expenses.

（1）**Construction**（**work volume of construction**）refers to the construction of houses and buildings, They include construction of houses; equipment foundations, industrial kilns and stoves, and metal structure work; preparation works and temporary works for project construction, and clearing up works post project construction; pavement of railways and roads, drilling of mines and putting up of oil pipes; construction of water conservancy; construction of underground air-raid shelters and construction of other special projects; value of equipment for heating, sanitation, ventilation, lighting, gas, painting, etc. that are covered by the budget of housing projects; laying out of various pipelines （for steam, compressed air, petroleum, tap water and sewage） and wiring and cabling for electric power and for communications; installation of various machinery and equipment; testing operation for pre-testing the quality of installation projects, and land and other development work conducted by real estate developers for commercialized housing.

（2）**Installation** （**work volume of installation**）refers to the installation of various kinds of equipment and instruments, the installation projects connected with the equipment and other projects attach to the installation equipment; preparing work of the equipment to test the quality of the installation projects. The value of equipment installed is itself not included in the value of installation projects.

（3）**Purchase of equipment and instrument s**refers to the total value of equipment, tools, and instruments purchased or self-produced which come up to the cut-off point for fixed assets by the construction units or investing enterprises or institutions. Equipment, tools and instruments purchased or self-produced for new workshops by newly established or expanded units are categorized as "purchase of equipment and instruments" no matter whether they come up to the cut-off point for fixed assets.

（4）**Other Expenses** refer to expenses arising during the construction or purchase of fixed assets other than those mentioned above.

**Newly Increased Fixed Assets** refer to the newly increased value of fixed assets, constructed or purchased, that have been transferred to the investors. This is an indicator that demonstrates the results of investment in fixed assets in monetary terms, and an important indicator to reflect the speed of construction and to calculate the efficiency of investment.

**Floor Space under Construction** refers to total floor space of all buildings under construction during the reference period, including floor space of newly started buildings during the reference period, floor space of construction extended from the previous period to the current period, and floor space of construction suspended during the previous period and resumed in the current period. Floor space of construction completed in the current period, and floor space of construction started and then suspended in the current period are also included in the floor space under construction of the current year.

**Floor Space of Newly Started** refers to the total floor space of buildings of newly started in the reference period and its accounting coverage is the unit construction. It is the total floor space of the whole building and it should be calculated as a whole.

**Floor Space Completed** refers to the floor space of all buildings completed in the reference period, which has been appraised and accepted（or come up to the designed standards）and has been transferred to owner units.

**Value of Floor Space Completed** refers to the value of floor space of all buildings completed in the reference period, it is calculated according to the building design and the content of the budget regulation.

**Projects under Construction** refer to projects with construction and installation activities undertaken in the reference period. The number of projects under construction can reflect the actual size of investment in fixed assets during a given period, and when compared with the number of projects completed and put into use during the same period, it demonstrates the results of investment in fixed assets from the angle of the speed of the construction. Depending on the nature of construction activities, projects under construction can also be classified into projects beginning construction in current year, winding-up projects in current year and stopped or suspended projects in previous years （with resumption of work in current year）.

**Projects Completed and Put into Use** refers to the construction projects which have been completed the main work and responding supporting facilities which has built up the productivity or project benefit in accordance with the design documents, have been checked and accepted after relevant examination; and have been formally delivered for use in the reference period.

**Newly Increased Production Capacity**（**or Project Efficiency**）refers to the increase in design capacity （or project efficiency） through investment in fixed assets, which reflects the accomplishment of investment in fixed assets in physical form, and its calculating coverage is the construction which can produce the production capacity and project efficiency independently and has been appraised and accepted （or come up to the designed standards）and has been transferred to use formally when it is completed.

**Plan land area for Use** refers to the land area of the construction projects and the project planning which have been approved by relevant department.

**Actual Requisitioning and Purchased Land Area** refers to the area of the land obtained by requisition and other ways in the reference period.

**Actual Sales Amount of Requisitioning and Purchasing Land** refers to the final amount of money spent on requisitioning and purchasing land in the reference period. In order to calculating the average requisitioning land and purchasing price correctly, the coverage of requisitioning and purchasing land area is same as that of actual price of requisitioning and purchasing land.

**Sources of Funds for Investment in Fixed Assets** are categorized as surplus fund from the year-end of preceding year and source of funds this year ,depending on the sources of investment. Source of funds this year are categorized as follows:

（1）**Fund from the State budget consists of budgetary appropriation includes central budget funds and local budget funds.central budget funds includes funds** from the budget of the central government, capital construction fund （operation fund and non-operational fund）, special expenses （e.g. expenses on substituting petroleum with coal）, loans from repayment, discount fund, expenses on innovation and trial production of new products, expenses on urban construction, expenses on temporary construction from business departments, development fund for less developed areas, as well as local budgetary fund transferred from the central budget.

（2）**Domestic loans** refer to loans of various forms borrowed by investing units from banks and non-bank financial institutions during the reference period for the purpose of investment in fixed assets, including loans issued by banks from their self-owned funds and deposit, loans appropriated by higher authorities, special loans by government, loans arranged by local government from special funds, domestic reserve loan, and working loan.

（3）**Bond** refers to the funds raised by enterprises （corporations） or financial institutions through bonds issue and used for the investment in fixed assets, including the key enterprises bond and fundamental construction bond issued by banks deputized for national professional investment corporations.

（4）**Foreign investment** refers to foreign funds received during the reference period for the construction and purchase of investment in fixed assets （covering equipment, materials and technology）. In calculating the utilization of foreign capital, foreign currencies are converted into Chinese Renminbi applying the current exchange rate when the foreign capitals are actually used.

（5）**Self-raised funds** refer to extra-budgetary funds for investment in fixed assets received during the reference period by investing units from central government ministries, local governments, enterprises and institutions, including their self-raised funds.

（6）**Others** refer to funds for investment in fixed assets received from sources other than those listed above, including capital raised through issuing bonds by enterprises or financial institutions, funds raised from individuals and through donations, and funds transferred from other units.
