# Supplementary material for: The measurement of competitiveness of forest green food industry in Yunnan Province
Source: PLoS One. 2021 Dec 10;16(12):e0261133. doi: 10.1371/journal.pone.0261133 (PMC8664197; doi:10.1371/journal.pone.0261133)
Supplement: S1 File — http://stats.yn.gov.cn/. (Z01) [file pone.0261133.s001.z01 › the Yunnan statistical yearbook (2016-2020)/2016/4/经贸.docx]

主要统计指标解释

批发业  指向其他批发或零售单位（含个体经营者）及其他企事业单位、机关团体等批量销售生活用品、生产资料的活动，以及从事进出口贸易和贸易经纪与代理的活动，包括拥有货物所有权，并以本单位(公司)的名义进行交易活动,也包括不拥有货物的所有权，收取佣金的商品代理、商品代售活动；还包括各类商品批发市场中固定摊位的批发活动，以及以销售为目的的收购活动。

零售业  指百货商店、超级市场、专门零售商店、品牌专卖店、售货摊等主要面向最终消费者（如居民等）的销售活动，以互联网、邮政、电话、售货机等方式的销售活动，还包括在同一地点，后面加工生产，前面销售的店铺（如面包房）；谷物、种子、饲料、牲畜、矿产品、生产用原料、化工原料、农用化工产品、机械设备（乘用车、计算机及通信设备除外）等生产资料的销售不作为零售活动；多数零售商对其销售的货物拥有所有权，但有些则是充当委托人的代理人，进行委托销售或以收取佣金的方式进行销售。

批发和零售业商品购进、销售、库存额  指各种登记注册类型的批发和零售业企业(单位)以本企业(单位)为总体的，从国内、国外市场购进的商品总量，销售和出口的商品总量，库存的商品总量等情况。该指标可以反映商品流转过程中商品的购进、销售、库存之间的比例关系和存在的问题。

商品购进额  指从本企业以外的单位和个人购进（包括从国外直接进口）作为转卖或加工后转卖的商品金额（含增值税）。商品购进包括：（1）从工农业生产者、批发和零售业企业、住宿和餐饮业企业、出版社或报社的出版发行部门和其他服务业企业购进的商品；（2）从机关团体、事业单位购进的商品；（3）从海关、市场管理部门购进的缉私和没收的商品；（4）从居民收购的废旧商品等。不包括：（1）企业为本单位自身经营用，不是作为转卖而购进的商品，如材料物资、包装物、低值易耗品、办公用品等；（2）未通过买卖行为而收入的商品，如接受其他部门移交的商品、借入的商品、收入代其他单位保管的商品、其他单位赠送的样品、加工回收的成品等；（3）经本单位介绍，由买卖双方直接结算，本单位只收取手续费的业务；（4）销售退回和买方拒付货款的商品；（5）商品溢余。

商品销售额  指对本单位以外的单位和个人出售的商品金额（包括售给本单位消费用的商品，含增值税）。商品销售包括（1）售给城乡居民和社会集团消费用的商品；（2）售给农业、工业、建筑业、服务业等国民经济各行业用于生产、经营用的商品，包括售予批发和零售业作为转卖或加工后转卖的商品；（3）对国（境）外直接出口的商品。不包括：（1）未通过买卖行为付出的商品，如随机构变动移交给其他企业单位的商品、借出的商品、归还受其他单位委托代保管的商品、付出的加工原料和赠送给其他单位的样品等；（2）经本单位介绍，由买卖双方直接结算，本单位只收取手续费的业务；（3）购货退回的商品；（4）商品损耗和损失；（5）出售本单位自用的废旧物资。

商品库存额  对于批发和零售业法人单位和个体经营户，是指报告期末取得所有权的全部商品金额（含增值税）；对于批发和零售业产业活动单位，是指报告期末实际在库且归属法人具有所有权的全部商品金额（含增值税）。库存商品包括：(1)存放在本单位(如门市部、批发站、采购站、经营处)的仓库、货场、货柜和货架中的商品；(2)挑选、整理、包装中的商品；(3)已记入购进而尚未运到本单位的商品，即发货单或银行承兑凭证已到而货未到的商品；(4)寄放他处的商品，如因购货方拒绝付款而暂时存在购货方的商品；(5)委托其他单位代销(未作销售或调出)尚未售出的商品；(6)代其他单位购进尚未交付的商品。不包括：所有权不属于本单位的商品；委托外单位加工的商品；外贸企业代理其他单位从国外进口，尚未付给订货单位的商品；代国家储备部门保管的商品。

社会消费品零售总额  指企业（单位、个体户）通过交易直接售给个人、社会集团非生产、非经营用的实物商品金额，以及提供餐饮服务所取得的收入金额。个人包括城乡居民和入境人员，社会集团包括机关、社会团体、部队、学校、企事业单位、居委会或村委会等。

住宿业  指为旅行者提供短期留宿场所的活动，有些单位只提供住宿，也有些单位提供住宿、饮食、商务、娱乐一体的服务，不包括主要按月或按年长期出租房屋住所的活动。

餐饮业  指通过即时制作加工、商业销售和服务性劳动等，向消费者提供食品和消费场所及设施的服务。

营业额  指住宿和餐饮业单位在经营活动中因提供服务或销售商品等取得的收入。包括：客房收入、餐费收入、商品销售额（含增值税）和其他收入。其中，客房收入指住宿和餐饮业单位在经营活动中因提供住宿服务取得的收入。餐费收入指本单位为顾客提供就餐服务取得的收入，包括：经烹饪、调制加工后出售的各种食品，如主食、炒菜、凉拌菜等的收入。

入境游客  指报告期内来中国（大陆）观光、度假、探亲访友、就医疗养、购物、参加会议或从事经济、文化、体育、宗教活动的外国人、港澳台同胞等游客（即入境旅游人数）。统计时，入境游客按每入境一次统计1人次。入境旅游人数包括入境过夜游客和入境一日游游客。

出境人数（出境游客）  指中国（大陆）居民因公或因私出境前往其他国家、中国香港特别行政区、澳门特别行政区和台湾省观光、度假、探亲访友、就医疗养、购物、参加会议或从事经济、文化、体育、宗教活动的人数（即出境游客）。统计时，出境游客按每出境一次统计1人次。

国内游客  指报告期内在中国（大陆）观光游览、度假、探亲访友、就医疗养、购物、参加会议或从事经济、文化、体育、宗教活动的中国（大陆）居民人数，其出游的目的不是通过所从事的活动谋取报酬。统计时，国内游客按每出游一次统计1人次。

国际旅游(外汇)收入  指入境游客在中国（大陆）境内旅行、游览过程中用于交通、参观游览、住宿、餐饮、购物、娱乐等全部花费。

国内旅游收入(旅游总花费)  指国内游客在国内旅行、游览过程中用于交通、参观游览、住宿、餐饮、购物、娱乐等全部花费。

**Wholesale Trade** refers to the activities of selling wholesale commodities for daily use and capital goods to enterprises of wholesale and retail trades (including self-employed individuals) and other enterprises, institutions and government organs and organizations, and the activities of engaging in import and export and acting as a trade agent. The wholesaler may have the ownership of the commodities for wholesale and trade in the name of its own (a company), and the wholesaler can act as commission agent or commodity broker without the ownership of commodities. Also included are the wholesale activities at the fixed stalls in wholesale market and the acquisition for sales purpose.

**Retail Trade** refers to the activities of department store, supermarket, franchised store, brand store, retail stall and on-the-spot-making-selling store selling commodities to the final consumers (residents) by any means including internet, post, telephone, sales machine. It also includes shops with sales and production located in the same places (such as bakeries). Retail trade excludes the activities of sales of capital goods such as grain, seed, feed, livestock, mineral products, raw material for production, industrial chemicals, chemical products for agricultural use, machine and equipment (excluding vehicles, computers and communication equipment). Most retailers have the ownership of commodities to sell, but some are acting as agents or brokers to make transactions for a commission.

**Purchase, Sales and Stock of Commodities by Wholesale and** **Retail Trades** refer to the total volume of commodities purchased, total volume of sales and exports, and the stock of commodities by wholesale and retail enterprises (establishments) of different status of registration from domestic and overseas markets. This indicator reflects the relationship among purchase, sales and stock of commodities in the circulation of goods and reveals the existing problems.

**Total Purchases of Commodities** refer to the total value of purchases of commodities by enterprises (establishments) from other establishments or individuals (including direct import from abroad) for the purpose of re-selling, either with or without further processing of the commodities purchased. The commodities include: (1) commodities purchased from agricultural and industrial producer, wholesaler, retailer, publishing house and other service business; (2) commodities purchased from institutions and government departments; (3) confiscated goods purchased from the customs authorities or market management agencies; (4) second-hand goods and wastes purchased from residents; The commodities exclude (1) commodities purchased by enterprises (establishments) for use in their own business operation, commodities obtained without buying or selling procedures such as materials, consumable goods of low value, office appliance, etc. (2) received goods without trading, such as goods handed over from others, borrowed goods, preserved goods for others, donated goods from others, processed and retrieved goods, etc. (3) goods of direct settlement between buyer and seller with handling fees introduced by others, (4) goods returned or refused to pay by the buyer, (5) excessive goods.

**Total Sales of Commodities** refer to value of commodities sold by the establishments to other establishments and individuals (including goods sold for self consumption, including the value-added tax). The commodities include: (1) commodities sold to urban and rural residents and social groups for their consumption; (2) commodities sold to establishments in all industries for their production and operation, including  agriculture, industry, construction, and catering services including commodities sold to wholesale and retail establishments for re-selling, with or without further processing; and (3) commodities for direct export to abroad. Excluded are (1) extended commodities without trading, such as goods handed over to other enterprises and institutions because of the change of organizations, lent goods, returned goods preserved for others, extended processing materials and samples donated to others, (2)  goods of direct settlement between buyer and seller with handling fees introduced by others, (3) goods returned after purchase, (4) damaged and spoiled goods, (5) waste and used goods of self use,

**Total Stock of Commodities** For the legal entities and self-employed individuals engaged in wholesale and retail trade, it refers to total value (including VAT) of commodities possessed at the end of the reference period; and for wholesale and retail establishments, it refers to the value (including VAT) of all commodities actually in stock and owned by their legal persons at the end of reference period. The commodities in stock includes: (1) commodities located in storage, garages, counters, and shelves of operating places of wholesale and retail trades (such as sale stores, wholesale centres, procurement stations and operating offices); (2) commodities in the process of being selected, sorted, and packed; (3) commodities not arrived but recorded as purchase in the account, i.e. commodities not arrived but payment receipts for the commodities from the sellers or the banks arrived; (4) commodities deposited in other places rather than places mentioned above, for instance: commodities in the hold of purchasers temporarily due to the refusal of payment; (5) commodities entrusted to other units to sell but not sold yet; (6) commodities purchased for other units but not delivered yet. Commodities not included as stock are those not owned by the enterprises (units), commodities on commission for processing, imported commodities of agency of foreign trade enterprise but not yet delivered to ordering units and finally those put in stock on behalf of the state reserves units.

**Large Commodity Markets with Transaction Value over 100 Million Yuan** refers to the commodity markets with an annual transaction at and above 100 million. The commodity market refers to the markets approved and managed by related departments, where there are fixed sites, facilities, managers and administration offices, where there are a certain number of traders to operate for three month and above or all the year, where the commodities including the articles for daily consumption and capital goods and services are traded in a centralized, independent and open way. Such market includes markets of daily goods and market of capital goods, etc.

**Total Retail Sales of Consumer Goods** refer to the amount obtained by enterprises (units, self-employed individuals) through direct sales of non-production and non-business physical commodity to individuals, social institutions, and revenue from providing catering services. Individuals include rural and urban households, population from abroad, social institutions include government agencies, social organizations, military units, schools, institutions, neighbourhood (village) committees.

**Hotel Services** refer to the accommodation services provided to visitors. Some units may provide only accommodation while others provide a combination of accommodation, meals, business services and/or recreational facilities. It excludes activities related to the provision of long-term primary residences in facilities such as apartments typically leased on a monthly or annual basis.

**Catering Services**  refer to the activities of providing foods, serving locations and facilities to customers through instant processing, commercial sales and service-type labor.

**Business Revenue** refers to revenue of hotels and catering services received from providing services or selling commodities through business activities, including income from hotels, from catering services, from selling of commodities (including VAT) and from other services. Income from hotels refers to income of hotels and catering services by providing lodging services through business activities. Income from catering services refers to income from providing catering services, including selling of cooked or prepared foods, such as staple food, cooked dishes, or cold dishes.
