# Supplementary material for: The measurement of competitiveness of forest green food industry in Yunnan Province
Source: PLoS One. 2021 Dec 10;16(12):e0261133. doi: 10.1371/journal.pone.0261133 (PMC8664197; doi:10.1371/journal.pone.0261133)
Supplement: S1 File — http://stats.yn.gov.cn/. (Z01) [file pone.0261133.s001.z01 › the Yunnan statistical yearbook (2016-2020)/2016/5/主要指标解释.doc]

主要统计指标解释

财政收入 指国家财政参与社会产品分配所取得的收入，是实现国家职能的财力保证。

地方财政一般预算收入 是指按照一定的形式和程序，由地方各级财政部门组织并纳入地方财政一般预算管理的各项收入。主要包括：

1.税收收入：包括国内增值税、营业税、企业所得税、个人所得税、资源税、城市维护建设税、各银行总行、各保险公司总公司集中交纳的部分、房产税、印花税、城镇土地使用税、土地增值税、车船税、耕地占用税、契税、烟叶税等。

2.非税收入：包括专项收入、行政事业性收费、罚没收入、国有资本经营收入、国有资源（资产）有偿使用收入和其他收入。

财政支出 指国家财政将筹集起来的资金进行分配使用，以满足经济建设和各项事业的需要。

地方财政一般预算支出 是指地方各级财政部门对所集中的预算收入有计划地分配和使用而安排的支出。主要包括：

1.一般公共服务：指政府提供基本公共管理与服务的支出，包括人大事务、政协事务、政府办公厅（室）及相关机构事务、发展与改革事务、统计信息事务、财政事务、税收事务、审计事务、海关事务、人力资源事务、纪检监察事务、人口与计划生育事务、商贸事务、知识产权事务、工商行政管理事务、国土资源事务、海洋管理事务、测绘事务、地震事务、气象事务、民族事务、宗教事务、港澳台侨事务、档案事务、共产党事务、民主党派事务及工商联事务、群众团体事务、彩票事务等。

2.公共安全：指政府维护社会公共安全方面的支出，包括武装警察、公安、国家安全、检察、法院、司法行政、监狱、劳教、国家保密、缉私警察等。

3.教育：指政府教育事务支出，包括教育行政管理、学前教育、小学教育、初中教育、普通高中教育、普通高等教育、初等职业教育、中专教育、技校教育、职业高中教育、高等职业教育、广播电视教育、留学生教育、特殊教育、干部继续教育、教育机关服务等。

4.科学技术：指用于科学技术方面的支出，包括科学技术管理事务、基础研究、应用研究、技术研究与开发、科技条件与服务、社会科学、科学技术普及、科技交流与合作等。

5.文化教育与传媒：指政府在文化、文物、体育、广播影视、新闻出版等方面的支出。

6.社会保障和就业：指政府在社会保障与就业方面的支出，包括社会保障和就业管理事务、民政管理事务、财政对社会保险基金的补助、补充全国社会保障基金、行政事业单位离退休、企业改革补助、就业补助、抚恤、退役安置、社会福利、残疾人事业、城市居民最低生活保障、其他城镇社会救济、农村社会救济、自然灾害生活救助、红十字事务等。

7.城乡社区事务：指政府城乡社区事务支出，包括城乡社区管理事务支出、城乡社区规划与管理支出、城乡社区公共设施支出、城乡社区住宅支出、城乡社区环境卫生支出、建设市场管理与监督支出等。

8.农林水事务：指政府农林水事务支出，包括农业支出、林业支出、水利支出、扶贫支出、农业综合开发支出等。

9.交通运输：指政府交通运输和邮政业方面的支出，包括公路运输支出、水路运输支出、铁路运输支出、民用航空运输支出、邮政业支出等。

10.工业商业金融等事务：指政府对工业、商业及金融等方面的支出，包括采掘业支出、制造业支出、建筑业支出、工业和信息产业监管支出、国有资产监管支出、商业流通事务支出、金融业监管支出、旅游业管理与服务支出等。

**Explanatory Notes on Principle Statistical Indicators**

**Government Revenue** refers to income for the government finance through participating in the distribution of social products. It is the financial guarantee to ensure government functioning.

**Local Government Budgetary Revenue** refers to incomes which were organized and managed by the financial departments of local governments at all levels according to certain forms and procedures.

The contents of the local government budgetary revenue include the following main items:

**1.Tax Revenues**, including domestic value added tax （VAT）, domestic consumption tax, VAT and consumption tax from imports, VAT and consumption tax rebate for exports, business tax, corporate income tax, individual income tax, resource tax, city maintenance and construct tax, house property tax, stamp tax, urban land use tax, land appreciation tax, tax on vehicles and boat operation, ship tonnage tax, vehicle purchase tax, tariffs, farm land occupation tax, deed tax, and tobacco leaf tax, etc.

**2.Non-tax revenue**, including special program receipts, charge of administrative and institutional units, penalty receipts and others non-tax receipts.

**Government Expenditure** refers to the distribution and use of the funds which the government finance has raised, so as to meet the needs of economic construction and various causes.

**Local Government Budgetary Expenditure** refers to the planned distribution and use of the raised budgetary revenue by the financial departments of local government at all levels.

It includes the following main items

**1.Expenditure for general public services:** It refers to the spending on the basic public management and services which provided by governments, including the expense on affairs of People’s Congress, affairs of People’s Political Consultative Conference, affairs of government general office and relative institutions, affairs of development and reform, affairs of statistics, affairs of finance, affairs of taxation, affairs of audit, affairs of customs, affairs of human resources and social security, affairs of discipline inspection and supervision, affairs of population and family planning, affairs of commerce and trade, affairs of intellectual property, affairs of administration for industry and commerce, affairs of land and resources, affairs of oceanic administration, affairs of surveying and mapping, affairs of earthquake, ethnic affairs, religious affairs, affairs of Hong Kong, Macao, Taiwan, and Overseas Chinese, affairs of archives administration, affairs of Chinese Communist Party, affairs of democratic parties and federation of industry and commerce, affairs of mass organization, and affairs of lottery, etc.

**2.Expenditure for public security:** It refers to the spending of government on maintaining social and public security, including the expense on armed police force, public security, state security, prosecution, courts, justice, prison, labour education and rehabilitation, protection of state secrecy, anti-smuggling police, etc.

**3.Expenditure for education:** It refers to the spending of government on education, including the expense on the administration of education, pre-primary education, primary education, secondary education, high school education, regular higher education, primary vocational education, secondary vocational education, technical school education, vocational high school education and higher vocational education, radio and television education, student abroad education, special education, on the job training of cadres, education authorities services, etc.

**4.Expenditure for Science and Technology:** It refers to the spending of government on science and technology （S&T）, including the expense on the administration of S&T, basic research, applied research, research and development, conditions and services of S&T, popularization of social science, science and technology, exchanges and cooperation of S&T, etc.

**5.Expenditure for Culture, Sport and Media:** It refers to the spending of government on culture, cultural heritage, sports, radio, film, television, press and publication, etc.

**6.Expenditure for Social Safety Net And Employment Effort:** It refers to the spending of government on social safety net and employment, including the expense on administration of social safety net and employment, civil affairs, budgetary subsidy on the social insurance funds, subsidy on National Social Security Fund, retirees of administrative units and institutions, subsidy on enterprise reform, subsidy on employment effort, pension, placement of ex-serviceman, social welfare, the handicapped undertakings, the system of cost of living allowances for urban residents, other urban social relief, rural social relief, living relief of natural disasters, affairs of Red Cross Society, etc.

**7.Expenditure for Urban and Rural Community Affairs:** It refers to the spending of government on urban and rural community affairs, including the expense on administration of urban and rural community, planning and management of urban and rural community, public facilities of urban and rural community, housing of urban and rural community, sanitation of urban and rural community, management and supervision on the construction market, etc.

**8.Expenditure for Agriculture, Forestry and Water Conservancy:** It refers to the spending of government on agriculture, forestry and water conservancy, including the expense on agriculture, forestry, water conservancy, poverty alleviation, comprehensive agricultural development, etc.

**9.Expenditure for Transportation:** It refers to the spending of government on transportation and postal services, including the expense on road transportation, waterway transportation, railway transportation, civil aviation transportation, and postal services.

**10.Expenditure for Industry, Commerce and Banking:** It refers to the spending of government on industry, commerce and banking, including the expense on mining, manufacturing, construction, industry and information technology supervision and administration, State-owned assets supervision and administration, commerce and circulation affairs, financial intermediation supervision and administration, tourism administration and service, etc.
