# Supplementary material for: The measurement of competitiveness of forest green food industry in Yunnan Province
Source: PLoS One. 2021 Dec 10;16(12):e0261133. doi: 10.1371/journal.pone.0261133 (PMC8664197; doi:10.1371/journal.pone.0261133)
Supplement: S1 File — http://stats.yn.gov.cn/. (Z01) [file pone.0261133.s001.z01 › the Yunnan statistical yearbook (2016-2020)/2016/6/指标解释.docx]

主要统计指标解释

货物进出口总额  指实际进出我国国境的货物总金额。包括对外贸易实际进出口货物，来料加工装配进出口货物，国家间、联合国及国际组织无偿援助物资和赠送品，华侨、港澳台同胞和外籍华人捐赠品，租赁期满归承租人所有的租赁货物，进料加工进出口货物，边境地方贸易及边境地区小额贸易进出口货物(边民互市贸易除外)，中外合资企业、中外合作经营企业、外商独资经营企业进出口货物和公用物品，到、离岸价格在规定限额以上的进出口货样和广告品(无商业价值、无使用价值和免费提供出口的除外)，从保税仓库提取在中国境内销售的进口货物，以及其他进出口货物。该指标可以观察一个国家在对外贸易方面的总规模。我国规定出口货物按离岸价格统计，进口货物按到岸价格统计。

商品经营单位所在地进、出口额  指在所在地海关注册登记的有进出口经营权的企业实际进、出口额。

商品目的地进口额和商品货源地出口额  目的地进口额指进口货物的消费、使用或最终抵运地的实际进口额；货源地出口额指出口货物的产地或原始发货地的实际出口额。

服务进出口  指常住单位与非常住单位之间相互提供的服务。包括运输服务、旅游服务、通信服务、建筑服务、保险服务、金融服务、计算机和信息服务、咨询服务、广告宣传服务、电影音像服务、专有权利使用费和特许费、其他商务服务。不包括政府服务。

外商直接投资  是指外国投资者在我国境内通过设立外商投资企业、合伙企业、与中方投资者共同进行石油资源的合作勘探开发以及设立外国公司分支机构等方式进行投资。外国投资者可以用现金、实物、无形资产、股权等投资，还可以用从外商投资企业获得的利润进行再投资。

外商其他投资  指除对外借款和外商直接投资以外的各种利用外资的形式。包括企业在境内外股票市场公开发行的以外币计价的股票发行价总额，国际租赁进口设备的应付款，补偿贸易中外商提供的进口设备、技术、物料的价款，加工装配贸易中外商提供的进口设备、物料的价款。

对外直接投资  指我国企业、团体等(简称境内投资主体) 在国外及港澳台地区以现金、实物、无形资产等方式投资，并以控制国(境)外企业的经营管理权为核心的经济活动。对外直接投资的内涵主要体现在一经济体通过投资于另一经济体而实现其持久利益的目标。

对外承包工程  根据《对外承包工程管理条例》，对外承包工程是指中国的企业或者其他单位承包境外建设工程项目的活动。

对外劳务合作  指组织劳务人员赴其他国家或地区为国外的企业或机构工作的经营性活动。

**Explanatory Notes on Main Statistical Indicators**

**Total Import and Export of Goods**  refer to the real value of commodities imported and exported across the border of China. They include the actual imports and exports through foreign trade, imported and exported goods under the processing and assembling trades and materials, supplies and gifts as aid given gratis between governments and by the United Nations and other international organizations, and contributions donated by overseas Chinese, compatriots in Hong Kong and Macao and Chinese with foreign citizenship, leasing commodities owned by tenant at the expiration of leasing period, the imported and exported commodities processed with imported materials, commodities trading in border areas (excluding mutual exchange goods), the imported and exported commodities and articles for public use of the Sino-foreign joint ventures, cooperative enterprises and ventures with sole foreign investment. Also included is import or export of samples and advertising goods for which CIF or FOB value are beyond the permitted ceiling (excluding goods of no trading or use value and free commodities for export), imported goods sold in China from bonded warehouses and other imported or exported goods. The indicator of the total imports and exports at customs can be used to observe the total size of external trade in a country. In accordance with the stipulation of the Chinese government, imports are calculated at CIF, while exports are calculated at FOB.

**Import or Export Value by Location of China’s Foreign Trade Managing Units**  refers to actual value of imports and exports carried out by corporations which have been registered by the local Customs house and are vested with right to run import export business.

**Import Value of Commodities by Place of Destination and Export Value of Commodities by Place of Origin in China**  The former indicator refers to the value of import commodities of the places of their consumption, utilization or the places of their final destination. The latter indicator refers to the value of export commodities of the places of their origin or the places of the commodities dispatched.

**Import and Export of Services**  refers to services provided between resident and non-resident units, including services on transportation, tourism, communications, construction, insurance, banking, computer and information, consultancy, advertising and publicity, as well as film, audio and video services, royalty for patents, trade marks and other special rights, other commercial services, but excluding government services.

**Foreign Direct Investment** refers to foreign investment in China through the establishment of foreign invested enterprises, cooperative exploration and development of petroleum resources with domestic investors and the establishment of branch organizations of foreign enterprises. Foreign investment can be made in forms of cash, physical investment, intangible assets and equity, in addition with reinvestment of the foreign enterprises with the profits gained from the investment.

**Other Foreign Investment** refers to all forms of utilization of foreign capitals other than foreign borrowings and foreign direct investment. It includes the total value of stock shares in foreign currencies issued by enterprises at domestic or foreign stock exchanges, rent payable for the imported equipment through international leasing arrangement, cost of imported equipment, technology and materials provided by foreign counterparts in compensation trade and processing and assembly trade.

**Overseas Direct Investment**  refers to investment made by domestic enterprises and organizations (referred to as domestic investors) in foreign countries and Hong Kong SAR, Macao SAR and Taiwan province in forms of cash, physical investment and intangible assets, and the economic activities centring on operation and management of those enterprises are under the control of domestic investors. The content of overseas direct investment mainly reflects one economic entity by investing in another economic entity to achieve its goal of lasting interest.

**Overseas Contracted Projects** refer to activities of contracting overseas construction projects by Chinese enterprises or any other units, which are stipulated in the *Regulations on Administration of Foreign Contracted Project*.

**Overseas Labour Services** refer to operational activities of organizing labour force to go abroad providing services to foreign enterprises or agencies.
