# Supplementary material for: The measurement of competitiveness of forest green food industry in Yunnan Province
Source: PLoS One. 2021 Dec 10;16(12):e0261133. doi: 10.1371/journal.pone.0261133 (PMC8664197; doi:10.1371/journal.pone.0261133)
Supplement: S1 File — http://stats.yn.gov.cn/. (Z01) [file pone.0261133.s001.z01 › the Yunnan statistical yearbook (2016-2020)/2016/7/指标解释.docx]

主要统计指标解释

**农、林、牧、渔业总产值**指以货币形式表现的农林渔业全部产品总量和对农、林、牧、渔业生产活动进行的各种支持性服务活动的价值。它反映了一定时期（通常指一年）农、林、牧、渔业生产及其服务的总成果和总规模。

1957年以前的农业总产值包括了厩肥和农民自给性手工业（如农民自制衣服、鞋、袜，自己从事粮食初步加工等）。1958 年及以后的农业总产值，林业中增加了村及村以下竹木采伐产值；牧业中取消了厩肥产值；副业中取消了农民自给性手工业产值，增加了村及村以下的工业产值；渔业中增加了机械化捕鱼产值。1980 年及以后农业总产值，在副业中增加了农民商品性家庭手工业的产值。从1984 年起村及村以下办工业产值划归工业。1993 年取消副业产值，农业总产值改为农、林、牧、渔业总产值。原副业产值的采集野生植物和农民家庭兼营商品性工业划归农业产值；捕猎野兽野禽划归牧业产值。2003 年开始，增加农林牧渔服务业产值，同时取消农民家庭兼营商品性工业，竹木采运由村及村以下扩大到全社会口径。

**农、林、牧、渔业中间消耗**指各种经济类型的农业生产单位和农户在农业生产经营过程中投入（或消耗）的各种物质产品和劳务价值的总和。包括中间物质消耗和中间劳务消耗两个部分。计入中间消耗必须具备以下两个条件：一是与总产出相对应的生产过程中消耗的物质产品和劳务活动；二是本期投入并一次性消耗的不属于固定资产的非耐用品。

农、林、牧、渔业增加值指农、林、牧、渔及农林牧渔服务业生产货物或提供服务活动而增加的价值。增加值的计算方法有两种，一是生产法：农、林、牧、渔业增加值=农、林、牧、渔业总产出-农、林、牧、渔业中间消耗；二是分配法：农、林、牧、渔业增加值=固定资产折旧+劳动者报酬+生产税净额（生产税-生产补贴）+营业盈余。

自来水受益村数包括取水、净水、输配水三部分组成的自来水供给的，或由取水和输配水两部分的符合饮用卫生标准的简易自来水年末实际受益的村委会个数。

通汽车村数指拥有乡级以上公路通过，并通达客运或货运汽车的村委会个数。

粮食产量指全社会的产量。包括国营农场等全民所有制经营的、集体统一经营的和农民家庭经营的粮食产量，还包括工矿企业家属办的农场和其他生产单位的产量。粮食除包括稻谷、小麦、玉米、高粱、谷子及其他杂粮外，还包括薯类和大豆。其产量计算方法,豆类按去豆荚后的干豆计算；薯类（包括甘薯和马铃薯,不包括芋头和木薯）1963 年以前按每4 公斤鲜薯折1 公斤粮食计算，从1964 年以后按5公斤鲜薯折1 公斤粮食计算。其他粮食一律按脱粒后的原粮计算。

谷物指稻谷、小麦、玉米、谷子、高粱和其他谷物，不包括薯类和豆类。其他谷物指除稻谷、小麦、玉谷、高粱以外的一些子实主要用作粮食的作物，包括大麦、元麦（青稞）、莜麦、荞麦、糜子等。

油料产量指全部油料作物的生产量。包括花生、油菜籽、芝麻、向日葵子、胡麻子（亚麻子）和其他油料。不包括大豆、木本油料和野生油料。花生以带壳干花生计算。

水产品产量指人工养殖的水产品天然生长的水产品的捕捞量。包括海水的鱼类、虾蟹类、贝类和藻类以及淡水的鱼类、虾蟹类、贝类，不包括淡水水生植物。

猪、牛、羊产量指当年的猪、牛、羊的肉产量。即屠宰后除去头、蹄、下水后带骨肉（即胴体重）的重量。

期初(末)畜禽存栏头(只)数指报告期初(末)农村各种合作经济组织和国营农场、农民个人、机关、团体、学校、工矿企业、部队等单位以及城镇居民饲养的大牲畜、猪、羊、家禽等畜禽的存栏数。数据上报方式及数据调整情况同猪、牛、羊肉产量。

灌溉面积指有效灌溉面积，即具有一定的水源，地块比较平整，灌溉工程或设备已经配套，在一般年景下当年能够正常灌溉的耕地面积。

迹地更新面积在新、旧采伐迹地和火烧迹地上，进行人工更新或人工促进天然更新的面积（包括乔木林和灌木林）称迹地更新面积。迹地更新面积不包括未经人工措施的天然更新面积以及补植面积。

农作物播种面积指实际播种或移植有农作物的面积。凡是实际种植有农作物的面积，不论种植在耕地上还是种植在非耕地上，均包括在农作物播种面积中。在播种季节基本结束后，因遭灾而重新改种和补种的农作物面积，也包括在内。它是反映我国耕地面积利用情况的一个重要指标。目前，农作物播种面积主要包括粮食、棉花、油料、糖料、麻类、烟叶、蔬菜和瓜类、药材和其他农作物九大类。

农用化肥施用量指本年内实际用于农业生产的化肥数量，包括氮肥、磷肥、钾肥和复合肥。化肥施用量要求按折纯量计算数量。折纯量是指把氮肥、磷肥、钾肥分别按含氮、含五氧化二磷、含氧化钾的百分之百成分进行折算后的数量。复合肥按其所含主要成分折算。公式为：

折纯量=实物量×某种化肥有效成分含量的百分比

农业机械总动力指主要用于农、林、牧、渔业的各种动力机械的动力总和。包括耕作机械、排灌机械、收获机械、农用运输机械、植物保护机械、牧业机械、林业机械、渔业机械和其他农业机械（内燃机按引擎马力折成瓦（特）计算、电动机按功率折成瓦（特）计算）。不包括专门用于乡、镇、村、组办工业、基本建设、非农业运输、科学试验和教学等非农业生产方面用的动力机械与作业机械。这个指标的统计数据主要来源于农机部门。

Explanatory Notes on Principal Statistical Indicators

Gross Output Value of Farming, Forestry, Animal Husbandry and Fishery refers to the total value of products of farming, forestry, animal husbandry and fishery and the value of support services for production of farming, forestry, animal husbandry and fishery, which reflects the total scale and result of agricultural production and services during a given period (generally one year).

The gross agricultural output value before 1957 included output value of barnyard manure and farmers’ self-supporting handicraft industry (e.g., self-made clothing, shoes, socks, initial grain processing, etc.). Since 1958, output value of felling timber and bamboo by villages and organizations has been included in below village that of forestry; output value of barnyard manure has been excluded from that of animal husbandry; output value of farmers’ self-supporting handicraft industry has been excluded from that of sideline production, while output value of industries run by villages and organizations had been included in it; output value of mechanized fishing has been included in that of fishery. Since 1980, output value of farmers’ commercial household handicraft industry has been added to that of sideline production. Since 1984, output value of industries run by villages or organizations below village has been classified into that of industry. Since 1993, output value of sideline production has been cancelled and gross agricultural output value has been changed to gross output value of farming, forestry, animal husbandry and fishery; output value of wild plants gathering and commercial industry run by rural households has been incorporated to agricultural output value; output value of animal and bird hunting has been classified into that of animal husbandry. Since 2003, output value of services of farming, forestry, animal husbandry and fishery has been added while farmers’ commercial household industry has been cancelled, and transporting and felling timber and bamboo by villages and organizations below village has been expanded to all levels.

Intermediate Consumption of Farming, Forestry, Animal Husbandry and Fishery refers to the total value of material products and labor input (or consumed) by various agricultural production entities and rural households in the process of agricultural production and operation. It is composed of intermediate material consumption and intermediate labor consumption. Items calculated into intermediate consumption should satisfy the following two conditions: first, they are material products and labor consumed in the process of production against total output; second, they are is non-durable goods that do not belong to fixed assets but input and consumed up one time in the present phase.

Added Value of Farming, Forestry, Animal Husbandry and Fishery refers to the added Value produced by manufacturing goods or supplying service in farming, forestry, animal husbandry, fishery and service in support of farming,forestry, animal husbandry, fishery .There are two methods to calculate value added: one is the method of production: added value of farming, forestry, animal husbandry and fishery = total output of farming, forestry, animal husbandry and fishery - intermediate consumption of farming, forestry, animal husbandry and fishery; the other is the method of distribution: added value of farming, forestry, animal husbandry and fishery = depreciation of fixed assets +remuneration of laborers + net production tax (production tax — production subsidy) + business surplus.

Number of Villages Benefiting from Tap Water Supply refers to the number of villages practically enjoying tap water supply composed of water intakes, water treatment and water conveyance and distribution or up-to-standard potable water supply composed of water intakes and water conveyance and distribution at the year-end.

Number of Villages Accessible to Motor Vehicle refers to the number of villages with town-level road passing through and transport service.

Grain Yield refers to the total yield in the whole country including grain produced by state farms, collective entities, rural households, industrial enterprises and mines. Grain includes rice, wheat, maize, sorghum, millet and other cereals as well as tubers and soybeans. Output of beans refers to dry beans without pods. Output of tubers (sweet potatoes and potatoes, not including taros and cassava) was converted into that of grain at the ratio 4:1, i.e. 4 kilograms of fresh tubers was equivalent to 1 kilogram of grain up to 1963. Since 1964 the ratio for conversion has been 5:1. Tubers supplied as vegetables (such as potatoes) in cities and suburbs are calculated as fresh vegetables and their output is not included in the output of grain. Other kinds of grain are calculated as husked grain.

Cereals refer to rice, wheat, maize millet, sorghum and other kinds of grain, but tubers and beans are not included. Other kinds of grain refer to some crops whose seeds are mainly used for food such as barley, highland barley, naked oats, buckwheat, broom corn millet, etc.

Output of Oil-bearing Crops refers to the total production of oil-bearing crops of various kinds, including peanuts (dry, in shell), rapeseeds, sesame, sunflower seeds, flax seeds, and other oil-bearing crops. Soybeans, oil-bearing woody plants, and wild oil-bearing crops are not included.

Output of Aquatic Products refers to catches of both artificially cultured and naturally grown aquatic products, including fish, shrimps, crabs and shellfish in sea and inland water as well as seaweed. Freshwater plants are not included.

Output of Pork, Beef, and Mutton refers to the weight of the meat of slaughtered hogs, cattle, sheep and goats with head, feet, and offal taken away.

Number of Livestock or Poultry in Stock at Beginning (or End) of Period refers to the total number of large animals, pigs, sheep, fowls, etc. raised by rural cooperative organizations, State farms, rural individuals, government agencies, schools, industrial and mining enterprises, army, and urban residents at the beginning (or end) of the reference period. Data reporting system and data adjustment are the same as that in the output of pork, beef and mutton.

Irrigated Area refers to area under effective irrigation, i.e., area of cultivated land which is relatively level and has water source and complete sets of irrigation facilities to lift and move adequate water for irrigation purpose under normal conditions.

Reforested area refers to the area created by artificial reforestation or artificial measures promoting regeneration (including arbor and shrubbery forests) in new and old cut-over areas and burned areas. Reforested area excludes natural forest regeneration area and reinforcement planting area.

Sown Area of Crops refers to area of land sown or transplanted with crops regardless of being in cultivated area or non-cultivated area. Area of land resown due to natural disasters is also included. This is an important indicator that can reflect the utilization condition of the cultivated land in China. At present, the sown area of crops mainly include the following 9 categories of crops: grain, cotton, oil-bearing crops, sugar crops, flax crops, tobacco,

vegetables and melons, medicinal materials and other farm crops.

Consumption of Chemical Fertilizers in Agriculture refers to the quantity of chemical fertilizers applied in agriculture in the year, including nitrogenous fertilizer, phosphate fertilizer, potash fertilizer, and compound fertilizer. The consumption of chemical fertilizers is calculated in terms of volume of effective components by means of converting the gross weight of the respective fertilizers into weight containing effective component (e.g. nitrogen content in nitrogenous fertilizer, phosphorous pentoxide contents in phosphate fertilizer, and potassium oxide contents in potash fertilizer). Compound fertilizer is converted in regard to its major components. The formula is:

Volume of effective component= physical quantity× effective component of certain chemical fertilizer (%)

Total Power of Agricultural Machinery refers to total mechanical power of machinery used in agriculture, forestry, animal husbandry and fishery, including machinery for ploughing, irrigation and drainage, harvesting, transport, plant protection, animal husbandry, forestry and fishery and other agricultural machineries. (For the power of internal combustion engines, it is converted from its horsepower into watts while for electric motors the output power is converted into watts.) Machinery employed for non-agricultural purposes, such as the machines used in township-run and village-run industry, construction, non-agricultural transport, scientific experiments and teaching, are not included. Data are mainly from agricultural machinery agencies.
