# Supplementary material for: The measurement of competitiveness of forest green food industry in Yunnan Province
Source: PLoS One. 2021 Dec 10;16(12):e0261133. doi: 10.1371/journal.pone.0261133 (PMC8664197; doi:10.1371/journal.pone.0261133)
Supplement: S1 File — http://stats.yn.gov.cn/. (Z01) [file pone.0261133.s001.z01 › the Yunnan statistical yearbook (2016-2020)/2016/8/八、工业和能源.doc]

主要统计指标解释

工业 我国的工业包括以下四个方面的生产活动：

1.对自然资源的开采，如：采矿、晒盐等，但不包括禽兽捕猎、水产捕捞和森林采伐。

2.对农副产品的加工、再加工，如：粮油加工、食品加工、缫丝、纺织、制革等。

3.对采掘品的加工、再加工，炼铁、炼钢、轧钢、化工生产、石油加工、机械制造、木材加工等，以及电力、水、燃气的生产和供应等。

1984年以前农村的村及村以下办工业归属农业，1984年及以后划归工业。

独立核算法人工业企业和工业活动单位 工业统计调查单位分为三类：独立核算法人工业企业和工业活动单位及工业个体经营户。

独立核算法人工业企业 是指从事工业生产经营活动的单位。应同时具备以下条件：

1.依法成立,有自己的名称、组织机构和场所，能够承担民事责任。

2.独立拥有和使用资产，承担负债，有权与其他单位签订合同。

3.独立核算盈亏，并能够编制资产负债表。

工业活动单位 是指在一个场所从事一种或主要从事一种工业生产活动的经济单位。一般应同时具备以下三个条件：

1.具有一个场所，从事一种或主要从事一种工业活动。

2.单独组织工业生产、经营或业务活动。

3.单独核算收入和支出。

规模以上工业 2011年年报规模以上工业是指年主经营业务收入2000万元及以上工业企业。

国有经济（全民所有制工业） 是指生产资料归国家所有的一种经济类型。包括中央和地方各级国家机关、事业单位和社会团体使用国有资产和使用自有资金投资举办的工业企业。1957年以前的公私合营和私营工业,后均改造为国营工业，这部分工业资料不单独列时，均包括在国有经济内。

集体经济 是指生产资料归公民、集体所有的一种经济类型，包括城乡所有使用集体投资举办的工业企业,以及部分个人通过集体自愿放弃所有权并依法经工商行政管理机关认定为集体所有制的工业企业。

私营经济 是指生产资料归公民私人所有，以雇佣劳动力为基础的一种经济类型，包括私营独资企业,私营合伙企业和私营有限责任公司。

个体经济 是指生产资料归劳动者个人所有，以个体劳动为基础，劳动成果归劳动者个人占有和支配的一种经济类型。

“三资工业” 包括外商投资经济和港、澳、台投资经济。

其他经济 指除国有经济，集体经济，联营经济，股份有限公司，股份制经济，外商投资经济，港、澳、台投资经济，私营、个体经济等以外的其他经济。

轻工业 指提供生活消费品和制作手工工具的工业，是为满足人们吃、穿、用需要的工业。按其所使用的原料不同，可分为两大类：

1. 以农产品为原料的轻工业 是指直接或间接以农产品为基本原料的轻工业。主要包括食品制造、饮料制造、烟草加工、纺织、缝纫、皮革和毛皮制作、造纸以及印刷等工业。

2. 以非农产品为原料的轻工业 是指以工业品为原料的轻工业,主要包括文教体育用品、化学药品制造、合成纤维制造、日用化学制品、日用玻璃制品、日用金属制品、手工工具制造、医疗器械制造、文化和办公用机械制造等工业。

重工业 指生产生产资料的工业，是为国民经济各部门提供物质技术基础的工业。按其生产和产品用途，可分为下列三大类：

1. 采掘工业 是指对自然资源的开采，包括石油开采、煤炭开采、金属矿开采和非金属矿开采等工业。

2. 原材料工业 是指提供国民经济各部门使用的原料、动力和燃料的工业。包括金属冶炼及加工、炼焦及焦炭化学、化工原料、水泥、人造板以及电力、石油和煤炭加工等工业。

3. 加工工业 是指对原材料进行加工制造的工业。包括装备国民经济各部门的机械设备制造工业、金属结构、水泥制品等工业，以及为农业提供的生产资料和化肥、农药等工业。

根据上述划分原则，修理业中修理作业对象是重工业的划为重工业，反之划为轻工业。

大、中、小和微型企业 从2011年年报开始，企业规模的划分标准执行工业和信息化部、国家统计局、发展和改革委、财政部制定的《统计上大中小微型企业划分办法》，按照企业从业人员、主营业务收入，将企业划分为大型、中型、小型和微型。

工业总产值 是以货币表现的工业企业在一定时间内生产的工业产品总量，它反映工业生产的总规模和总水平。它包括：在本企业内不再进行加工，经检验、包装入库的成品价值、对外加工费收入、自制半成品、在制品期末期初差额价值。工业总产值采用“工厂法”计算，即以工业企业作为一个整体，按企业工业生产活动的最终成果计算，企业内部不允许重复计算，不能把企业内部各个车间（分厂）生产的成果相加。但在企业之间、行业之间、地区之间存在重复计算。

轻重工业总产值的划分也是按“工厂法”计算的，即一个工业企业在正常情况下生产的主要产品的性质属于轻工业，则该企业的全部总产值作为轻工业总产值；一个工业企业生产的主要产品的性质属于重工业，则该企业的全部总产值作为重工业总产值。

工业总产值计算遵循以下规定：

1.凡用自备原材料生产的产品，不论其加工的繁简程度如何，一律按全价，即工业总产值包括自备原材料的价值。

2.凡承接来料加工生产的产品，加工企业一律按财务上结算的加工费计算工业总产值，即不包括定货者来料的价值。

3.自制半成品、在制品期末期初差额价值，原则上应计入工业总产值，不再按生产周期是六个月以上还是六个月以下来区分是否计入工业总产值。

4.现价工业总产值一律采用不含销项税额的价格计算。

工业增加值 是指工业企业在一定时期内以货币表现的工业生产活动的最终成果。

工业企业主要财务指标

1. 资产总计 指企业过去的交易或者事项形成的、由企业拥有或者控制的、预期会给企业带来经济利益的资源。资产一般按流动性分为流动资产和非流动资产。其中流动资产可分为货币资金、交易性金融资产、应收票据、应收账款、预付款项、其他应收款、存货等；非流动资产可分为长期股权投资、固定资产、无形资产及其他非流动资产等。

2. 固定资产原价（原值） 指企业在建造、购置、安装、改建、扩建、技术改造某项固定资产时所支出的全部货币总额。一般包括买价、包装费、运杂费和安装费等。

3. 流动资产平均余额 流动资产是指可以在一年或者超过一年的一个营业周期内变现或者耗用的资产，包括现金及各种存款、短期投资、存货等；流动资产平均余额指全部流动资产报告期平均余额。计算公式为：

流动资产月平均余额=月初、月末流动资产余额之和÷2

流动资产季平均余额=季内各月流动资产平均余额之和÷3

流动资产年平均余额=1至12月各月流动资产平均余额之和÷12

工业企业主要经济效益指标

1. 全员劳动生产率 指根据产品的价值量指标的平均每一职工在单位时间内的产品生产量。目前我国的全员劳动生产率是用工业总产值或工业增加值除以同一时期全部职工的平均人数来计算的。计算公式为：

全员劳动生产率=工业总产值/全部职工平均人数×12/累计月份

或=工业增加值/全部职工平均人数×12/累计月份

2. 工业产品销售率 指报告期工业销售产值与同期全部工业总产值之比，反映工业产品生产已实现销售的程度。计算公式为：

工业产品销售率（%）=报告期现价工业销售产值/报告期现价工业总产值×100%

3. 工业资产利税率 指报告期已实现的利税总额与同期的资产（流动资产和固定资产净值）之比，反映企业资金运用的经济效益。计算公式为：

工业资产利税率（%）=报告期止累计实现利税总额/报告期平均流动资产+固定资产净值平均余额×12/累计月数×100%

4. 工业增加值率 指报告期工业增加值与同期工业总产值之比，反映降低中间消耗的经济效益。计算公式为：

工业增加值率（%）=报告期工业增加值/（报告期现价工业总产值（新规定）+报告期销项税额）×100%

5. 工业成本费用利润率 指报告期实现利润总额与成本费用之比，反映降低成本的经济效益。计算公式为。

工业成本费用利润率（%）=报告期实现利润总额/报告期成本费用总额×100%

成本费用是产品销售成本、产品销售费用、管理费用、财务费用之和

6. 流动资产周转率 指一定时期内流动资产完成的周转次数,反映流动资产的周转速度。计算公式为：

流动资产周转率=报告期止累计产品销售收入/报告期平均流动资产×12/累计月数

7. 资产负债率 又称债务比率，该比率反映在企业资产总额中有多少资产是通过借债而得到的。是反映企业长期偿债能力的指标之一，也可以用于衡量企业利用债权人提供资金进行经营活动的能力以及企业在清算时保护债权人利益的程度。计算公式为：

资产负债率=负债总额÷资产总额×100%

8. 总资产贡献率 是反映全部资产的获利能力，是企业管理水平和经营业绩的集中体现。计算公式为：

总资产贡献率（%）=利润总额＋利息支出/平均资产总额×12/累计月数×100%

平均资产总额为资产总计期初、期末之和的算术平均值。即：

平均资产总额=（期初资产总额＋期末资产总额）÷2

9. 资本保值增值率 反映企业资产的变动状况，是企业发展能力的集中体现。计算公式为：

资产保值增值率（%）= 期末所有者权益/期初所有者权益×100%

能源生产总量 指一定时期内全国（地区）一次能源生产量的总和。一次能源生产量包括原煤、原油、天然气、水电及其他动力能发电量（如风能、地热能等），不包括生物质能、太阳能等的利用和由一次能源加工转换而成的二次能源产量。能源生产总量是观察全国（地区）能源生产水平、规模、构成和发展速度的总量指标。

能源消费总量 指一定时期内全国（地区）用于生产和生活的各种能源消费量的总和。能源消费总量包括原煤和原油及其制品、天然气、电力的消费量，不包括生物质能和太阳能等的利用。能源消费总量分为三部分，即终端能源消费量、能源加工转换损失量和能源损失量。它是观察能源消费水平、构成和增长速度的总量指标。

1. 终端能源消费量 指一定时期内全国（地区）物质生产部门、非物质生产部门和生活消费的各种能源数量,不包括用于加工转换的中间能源消费量、加工转换损失量和损失量。

2.能源加工转换损失量 指一定时期内全国（地区）投入加工转换的各种能源数量之和与产出各种能源产品及其他石油制品和其他焦化产品之和的差额。它是观察能源在加工转换过程中损失量变化的指标。

3.能源损失量 指一定时期内能源在生产、输送、储存过程中发生的经营管理损失和由于客观原因造成的各种损失量，不包括各种气体能源放空、放散量。

能源生产弹性系数 是研究能源生产量的增长与国民经济增长之间的关系的指标。其计算公式为：

能源生产弹性系数=能源生产总量年平均增长速度/国民经济年平均增长速度

国民经济年平均增长速度,可根据不同的目的或需要,用工农业总产值、生产总值等指标来计算，本资料是采用生产总值指标计算的。

电力生产弹性系数 是研究电力生产量的增长与国民经济增长之间关系的指标。一般来说，电力的发展应当快于国民经济的发展，也就是说电力应超前发展。其计算公式为：

电力生产弹性系数=电力生产量年平均增长速度/国民经济年平均增长速度

能源消费弹性系数 是反映能源消费增长速度与国民经济增长之间比例关系的指标。其计算公式为：

能源消费弹性系数=能源消费年平均增长速度/国民经济年均增长速度

能源节约量 指在满足相等需求，达到相等目标的条件下节约和少用的能源数量。它是评价和考核节约能源工作好坏的重要指标。包括由于提高管理水平和技术水平，使单位产品能耗降低而节约能源的数量，以及由于调整产业结构、产品结构等使产值能耗降低而少用的能源数量。

能源节约率 是反映能源节约程度的综合性指标。能源节约率一般按年计算，如果要研究一个时期内能源节约程度的一般水平，可计算平均能源节约率指标。计算公式为：

能源节约率=（报告期单位能源消费量/基期单位能源消费量－1）×100%

年平均节能率=（n报告期单位能源消费量/基期单位能源消费源-1）×100%

公式中：单位能源消费量可以按生产总值、国民收入或工业总产值等计算

n 为基期与报告期间隔的年份数。

能源加工转换效率 指一定时期内能源经过加工转换后，产出的各种能源产品及其他石油制品和其他焦化产品的数量与同期内投入加工转换的各种能源数量的比率。它是观察能源加工转换装置和生产工艺先进与落后、管理水平高低等的重要指标。

能源折算标准 各种能源由于原始计算单位不同,热值也不一样。因此,必须折算成同一标准计算单位，才能进行汇总、对比和分析。国际上习惯采用两种标准计算单位：一种为标准煤，另一种为标准油。目前我国采用标准煤为能源的计算单位。标准煤亦称煤当量，就是将不同品种、不同含热量的能源按各自不同的含热量折合成为一种标准含量的统一计量单位的能源。各类能源折算标准煤是按1 公斤标准煤的热值为7000 千卡进行折算的。

当量热值当量 热值又称理论热值（或实际发热值），是指某种能源一个度量单位本身所含热量。其热值的计算可根据试样在充氧的弹筒中（放有浸没氧弹的水的容器）完全燃烧所放出的热量（用燃烧后水温升高计算出来的）进行实测。

等价热值 是指加工转换产出的某种二次能源与相应投入的一次能源的当量，即获得一个度量单位的某种二次能源所消耗的，以热值表示的一次能源量。也就是消耗一个度量单位的某种二次能源，就等价于消耗了以热值表示的一次能源量。等价热值是个变动值，随着能源加工转换工艺的提高和能源管理工作的加强，转换损失逐渐减少，等价热值会不断降低。等价热值是对二次能源及消耗工质而言，因此一次能源不存在折算问题，因此也无所谓等价热值。

等价热值=二次能源具有的能量÷转换效率

转换效率=二次能源产出标准量÷加工转换投入能源标准量

原材料、能源消费量 指在报告期内实际使用的原材料、能源的数量，包括企业主营活动和附营活动实际使用的数量。消费的核算原则为 “谁消费谁统计”，即按使用权来统计。核算方法为当进入第一道生产工序，改变原来的形态或性能、或已实际投入使用，即作消费统计。

原材料、能源库存量 指在报告期期初、期末实际结存的原材料、能源数量。库存的核算原则为“谁支配，谁统计”，即按所有权来统计。核算方法是指企业有权支配动用的某一时点实际结存的原材料、能源的数量。

**Explanatory Notes on Principal Statistical Indicators**

**Industry** refers to the material production sector which is engaged in the following four aspects:

1. Extraction of natural resources, such as mining, salt production, but not including hunting, fishing and logging;

2. Processing and reprocessing of farm and sideline produces, such as rice husking, food processing, flour milling, wine making, oil pressing, cotton ginning, silk reeling, spinning and weaving, and leather making;

3. Manufacturing of extracted products, such as steel making, iron smelting, chemicals manufacturing, petroleum processing, machine building, timber processing; water and gas production and electricity generation and supply;

Prior to 1984, the rural industry run by villages and cooperative organizations under village was classified into agriculture. Since 1984, it has been grouped into industry.

**Corporate Industrial Enterprises with Independent Accounting System and Industrial Activity Entities** Units of industrial statistics and inquiry are classified into two categories:corporate industrial enterprises with independent accounting system and industrial activity entities.

**Corporate Industrial Enterprises with Independent Accounting System** refer to enterprises engaging in industrial production activities, which meet the following requirements:

1. Established legally, having their own names, organizations, location, and being able to take civil liability;

2. Legally possessing and having the right to their assets independently, to assume liabilities, and to sign contracts with other entities;

3. Being able to calculate profit and loss independently and prepare their own balance sheets.

**Industrial Activity Entities** refer to the economic entities located in one single place and engaged entirely or primarily in one kind of industrial activity. Which generally meet the following requirements:

1. Having regular location and entirely or mainly engaging in one kind of industrial activity;

2. Operating and managing their industrial production activities independently;

3. Having independent accounting system for income and expenditures.

**Industrial Enterprises above Designated Size** refer to industrial enterprises with main business revenue reaches and exceeds 20 million yuan.

**State-owned Enterprises** （**Whole People Owned Industry**） refers to a type of industrial enterprises where the means of production are owned by the state. It includes the industrial enterprises run by the central and local state agencies at all levels and by institutions and social groups in using state-owned assets and self-owned funds. Joint state-private industries and private industries, which existed before 1957, have been transformed into state industries. Statistics on these enterprises has been included in the state-owned industries since 1957 when separation of data was no longer necessary.

**Collective-owned Enterprises** refers to a type of industrial enterprises where the means of production are owned collectively, including urban and rural enterprises invested by collectives and some enterprises which were formerly owned privately but have been registered in industrial and commercial administration agencies as collective entities through raising funds from the public.

**Private Enterprises** refers to a type of economic entities where the means of production are owned privately and employed labor force is taken as their basis. It includes private solely-funded enterprises, private partnership enterprises and private limited liability companies.

**Individual Enterprises** refers to a type of economic entities where the means of production are owned by individual laborer, individual labor is taken as their basis and labor fruits are owned by individual laborer.

3-**Capital Industry** includes foreign investment economy, Hong Kong, Macao and Taiwan investment economy.

**Other Enterprises** refers to other enterprises excluding state-owned, collective-owned, private-owned and individual enterprises. It includes joint ownership enterprises, joint stock enterprises, foreign funded enterprises, and enterprises funded by Hong Kong, Macao and Taiwan, etc.

**Light Industry** refers to the industry that produces consumer goods and hand tools, satisfying people’s need of eating, clothing and using. It consists of two categories, depending on the materials used:

1. Industries using farm products as raw materials. These are branches of light industry which directly or indirectly use farm products as basic raw materials, including food and beverages production, tobacco processing, textile, clothing, fur and leather making, paper making, printing, etc.;

2. Industries using non-farm products as raw materials. These are branches of light industry which use manufactured goods as raw materials, including the manufacture of cultural, educational articles and sports goods, chemicals, synthetic fiber, chemical products for daily use, glass products for daily use, metal products for daily use, hand tools, medical apparatus and instruments, and cultural and clerical machinery.

**Heavy Industry** refers to the industry, which produces capital goods, and provides various sectors of the national economy with necessary material and technical basis. It consists of the following three branches according to the purpose of production or the use of products:

**1. Mining, quarrying and logging industry** refers to the industry that extracts natural resources, including extraction of petroleum, coal, metal and non-metal ores and logging;

**2. Raw materials industry** refers to the industry that provides various sectors of the national economy with raw materials, fuels and power. It includes smelting and processing of metals, coking and coke chemistry, chemical materials and building materials such as cement, plywood, and power, petroleum refining and coal dressing;

**3. Manufacturing industry** refers to the industry that processes raw materials. It includes machine building industry which equips sectors of the national economy, industries of metal structure and cement products, industries producing means of agricultural production, such as chemical fertilizers and pesticides.

According to the above principle of classification, repairing trades which are engaged primarily in repairing products of heavy industry are classified into heavy industry while those engaged in repairing products of light industry are classified into light industry.

**Large, Medium, Small and Microenterprises** Since the bulletin of 2011,the standard of grouping the enterprises' scale has been changed according to Statistical Method of Classing Large, Medium, Small and Mini-sized Enterprises established by Ministry of Information and Industry, State Statistical Bureau, National Development and Reform Commission, Ministry of Finance. According to partitioning standards are number of employees, main operation revenue, enterprises are classified into large-sized, medium-sized, small-sized and microenterprises.

**Gross Industry Output Value** is the total volume of industrial products produced during a given period in monetary terms, which reflects the total achievements and overall scale of industrial production. It includes value of finished products, which are not to be further processed in the enterprises and have been inspected, packed and put in storage, income from external processing, and differential value of self-made semi-finished products and products in process at the end and beginning of the report period. The gross industrial output value is calculated by the “factory method”, i.e. an industrial enterprise is treated as the basic amounting unit in calculating the gross industrial output value; no double calculations are to be made within the same enterprise, e.g. the output value of the different workshops （branch factories） of an enterprises should not be added, however, this method does not exclude the possibility of double counting among different enterprises, industries and regions.

Output value of light and heavy industries is also classified by the “factory” method, i.e. if the major products of an industrial enterprise belongs to light industry products, the gross output value of that enterprise is classified wholly into light industry; the same principle applies to heavy industry.

**Calculation of gross Industry output value according to the following rules:**

1. Products produced with self-prepared raw material are calculated at all-round price in reporting the gross industrial output value, irrespective complexity of simplicity of production, i.e., the gross industrial output value includes the value of self-prepared raw material.

2. Products processed with supplied materials are calculated, according to processing charges financially settled in reporting the gross industrial output value, i.e., the gross industrial output value excludes the value of orders’ material.

3. Differential value of self-made semi-finished products and product in process at the end and beginning of the report period should be calculated into the gross industrial output value in principle and the old method in which inclusion or exclusion of the differential value is determined by whether the production cycle is over or below six months is not applied.

4. Current gross industrial output value is all calculated at price without sales tax.

**Added Value of Industry** refers to the final results of industrial production of industrial enterprises in monetary terms during the report period.

**Principal Finance Indicators of Industrial Enterprises**

**1.Total Assets** refer to all resources that are owned or controlled by enterprises through previous trades or transactions with expectation of making economic profits. Classified by the degree of liquidity,total assets include current assets, ad non-current assents.Current assets can be classified	into	monetary	assets,trading	financial	assets,notes receivable,accounts receivable,advanced payment, other prepaid money and inventories.Non-current assets can be divided into long-term equity investment, fixed assets,intangible assets and other non-current qssets.

**2. Original Value** of Fixed Assets refers to the value of payment made by enterprises, in building, purchase, installation, reconstruction, expansion, and technical transformation of a particular item of fixed assets, which includes expenses on purchase, package, transportation, and installation, etc.

**3. Net value** of fixed assets refers to the balance of the original value of fixed assets minus the amount of accumulated depreciation.

**4. Average balance of current assets:** current assets refer to the assets which can be liquidated or consumed within an operating cycle of one year or over one year, including cash and various deposits, short-term investment, inventory, etc.; average balance of current assets refers to the average balance of all the current assets in the report period. The formulae are as follows:

Monthly Average Balance of Current Assets=Sum of Balance of Current Assets at Beginning and End of Month÷2

Quarterly Average Balance of Current Assets=Sum of Balance of Current Assets in Each Month of Quarter÷3

Annual Average Balance of Current Assets=Sum of Balance of Current Assets in Each Month from January to December÷12

**Principal Indicators on Economic Performances of Industrial Enterprises**

**1. Overall Labor Productivity** refers to the average output per employed person of industrial enterprises in unit time in value terms. At present, gross industrial output value or added value of industry and average number of staff and workers of an industrial enterprise in a given period are used to calculate overall labor productivity. The formula used is as follows:

Overall Labor Productivity = Gross Industrial Output Value/Average Number of Staff and Workers× 12/Aunmulated Months or = Added Value of Industry/Average Number of Staff and Workers×12/Aunmulated Months

**2. Ratio of Sales to Gross Output Value** refers to the ratio of industrial sales value in the report period to gross industrial output value in the same period, which reflects the linkage between the industrial production and the realized sales. The formula is as follows:

Ratio of Sales to Gross Output Value = （Industrial Sales Value at Current Price in the Report Period/Gross Industrial Output Value at Current Price in the Report Period）×100%

**3. Ratio of Profits and Tax to Assets** refers to the ratio of total realized profits and tax in the report period to assets （net value of current assets and fixed assets） in the same period, which reflects the economic efficiency of fund utilization. The formula is as follows:

Ratio of Profits and Tax to Assets （%） = ［Total Accumulated Profit and Tax by the End of Report Period/（Average Current Assets in the Report Period + Average Balance of Net Value of Fixed Assets）］×12/Accumulated Months×100%

**4. Value-added Rate of Industry** refers to the ratio of added value of industry in the report period to gross output value in the same period, which reflects the economic efficiency of reduction in intermediate input and is calculated as follows:

Value-added Rate of Industry （%） = （Added Value of Industry in the Report Period/Gross Industrial Output Value at Current Price） in the Report Period + Sales Tax in the Report Period）×100%

**5. Ratio of Profits to Total Costs and Expenses** refers to the ratio of profits realized in the report period to the total costs and expenses in the same period, which reflects the economic efficiency of cost reduction. It is calculated as follows:

Ratio of Profits to Total Costs and Expenses （%） = （Total Profits Realized in the Report Period/Total Costs and Expenses in the Report Period）×100%

Costs and expenses are the sum of product sales cost, product sales expenses and financial expenses.

**6. Turnover Rate of Current Assets** refers to the number of times of turnover of current assets in a given period of time, which reflects the speed of the turnover of current assets. It is calculated as follows:

Turnover Rate of Current Assets （%） = （Accumulated Sales Revenue of Products by the End of Report Period/Average Current Assets in the Report Period）×12/Accumulated Months

**7. Ratio of Debts to Assets** reflects the proportion of assets obtained by borrowing in the total assets of an enterprise. It is one of the indicators reflecting the debt repaying capability of an enterprise in the long run and can also be used to measure the operating capability of an enterprise with the capital from creditors and the degree to which an enterprise can protect the interest of creditor during liquidation. The formula is as follows:

Ratio of Debts to Assets （%） = （Total Debts/Total Assets）×100%

**8. Contribution Rate of Total** Assets reflects the profit-making capability of all assets and it is a key indicator manifesting the performance and management level of an enterprise. The formula is as follows: Contribution Rate of Total Assets （%） = ［（Total Profits and Tax + Interest Payment）/Total Average Assets］× 12/Accumulated Months×100%

Total average assets refer to the arithmetic average value of total assets at the beginning and end of period, i.e. Total Average Assets = （Total Assets at the Beginning of Period + Total Assets at the End of Period）÷2.

**9. Rate of Asset Hedge and Increment** reflects the variation of assets of an enterprise and manifests the development capability of an enterprise. The formula is as follows:

Rate of Asset Hedge and Increment （%） = （Owner’s Equity at the End of Period/ Owner’s Equity at the Beginning of Period）×100%

**Total Energy Production Volume** refers to the total production volume of primary energy by all energy production enterprises in the country in a given period of time. It is a comprehensive indicator to show the capacity, scale, composition and development of energy production of the country. The production volume of primary energy includes that of coal, crude oil, natural gas, hydro-power and electricity generated by nuclear energy and other means such as wind power and geothermal power, but excludes that of fuels of low calorific value, bio-energy, solar energy and the secondary energy converted from the primary energy.

**Total Domestic Energy Consumption** refers to the total consumption of energy of various kinds by material production sectors, non-material production sectors and households in the country in a given period of time. It is a comprehensive indicator to show the scale, composition and development of energy consumption. The total energy consumption includes that of coal, crude oil and their products, natural gas and electricity, but excludes that of fuels of low calorific value, bio-energy and solar energy. It can be divided into three parts:

***1.Final energy consumption*** refers to the total energy consumption by material production sectors, non-material production sectors and households in the country （region） in a given period of time, but excludes the consumption in conversion of the primary energy into the secondary energy and the loss in the process of energy conversion.

***2.Loss in the process of energy conversion*** refers to the total input of various kinds of energy for conversion, minus the total output of various kinds of energy in the country in a given period of time. It is an indicator to show the loss that occurs in the process of energy conversion.

***3.Loss of energy*** refers to the total loss of energy during the course of energy transport, distribution and storage and the loss caused by any objective reason in a given period of time. The loss of various kinds of gas due to gas discharges and stocktaking is excluded.

**Elasticity Ratio of Energy Production** is an indicator to show the relationship between the growth rate of energy production and that of the national economy. The formula is as follows:

Elasticity Ratio of Energy Production = Average Annual Growth Rate of Energy Production/Average Annual Growth Rate of National Economy.

The average annual growth rate of the national economy can be calculated by gross output value of industry and agriculture, gross output value or other indicators, depending upon the purposes or needs. Gross output value is used in calculation of the ratio in this chapter.

**Elasticity Ratio of Electricity Production** is an indicator to show the relationship between the growth rate of electricity production that of the national economy. The formula is as follows:

Elasticity Ratio of Electricity Production = Average Annual Growth Rate of Electricity Production/Average Annual Growth Rate of National Economy.

**Elasticity Ratio of Energy Consumption** is an indicator to show the relationship between the growth rate of energy consumption and that of the national economy. The formula is as follows:

Elasticity Ratio of Energy Consumption = Average Annual Growth Rate of Energy Consumption/Average Annual Growth Rate of National Economy.

**Quantity of Energy Conservation** refers to the quantity of energy saved and less used in a certain period. It is an important indicator to appraise and examine the work of energy conservation. It includes the quantity of energy saved in unit product by improving management level and technology level and the quantity of energy less used due to the adjustment of industrial structure and product structure.

**Ratio of Energy Conservation** is a comprehensive indicator reflecting the degree of energy conservation. Ratio of energy conservation is usually calculated annually. The indicator of average ratio of energy conservation can be calculated for the study of the energy conservation in a certain period. The formula is as follows:

Ratio of Energy Conservation = ［（Unit Energy Consumption in the Report Period/Unit Energy Consumption in the Base Period） – 1］ ×100%

Annual Average Ratio of Energy Conservation = the N-the Root of ［（Unit Energy Consumption in the Report Period/Unit Energy Consumption in the Base Period） – 1］×100%

In which: The unit energy consumption can be calculated according to gross output value, national product or gross industrial output value, etc. The n represents the number of years between base period and report period.

**Efficiency of Energy Processing and Conversion** refers to the ratio of the total output of energy products of various kinds after processing and conversion to the total input of energy of various kinds for processing and conversion in the same report period. It is an important indicator to show the current conditions of energy processing and conversion equipment, production technique and management.

**Energy Conversion Standard** Different units are often used to compute different caloric value of various energy sources, so a uniform standard computing unit has to be conversed to summarize, compare and analyze energy. There are two standard computing units practiced internationally: one is standard coal and the other is standard oil. Currently, standard coal is adopted as the computing unit in China for energy calculation, Standard coal also call calorie value equivalent, it refers a uniform standard energy of communistically unit is converted into by differed kinds, different caloric value of various energy .Every kind of energy is converted into standard coal according to one kilogram standard coal quail caloric of 7,000 kilocalories.

**Equivalent Caloricity** also called theoretic caloricity （or actual calorific value） refers to the heat value contained in one measurement unit of certain energy. The calculation of the heat value is according to the actual measurement of the heat released by complete burning of the test specimen in the cylinder filled with oxygen （container with the oxygen bomb immersed by water）.

**Equivalent Heat Value** refers to certain secondary energy produced by conversion and its equivalent value of corresponding input of the primary energy, which means the quantity of the primary energy expressed by heat value and consumed to obtain one measurement unit of certain secondary energy. It is variation value. With the advancement of the energy conversion technology and the improvement of the energy management, the conversion loss becomes less and less, and the equivalent heat value will reduce gradually. Equivalent heat value is specific to secondary energy and consumed actuating medium, so when it comes into primary energy, there is no equivalent heat value.

Equivalent Heat Value = Energy Content of Secondary Energy÷Conversion Efficiency

Conversion Efficiency = Output Standardized Quantity of Secondary Energy ÷ Energy Standardized Quantity Input by Conversion

**Consumption of Raw Materials and Energy** refers to the quantity of raw materials and energy actually used in the report period. It includes the volume actually used in the main business line and sideline activities of an enterprise. The calculation principle of consumption is: “The one who consumes energy is responsible for conducting statistics on its consumption”, i.e., statistics is made according to the use right. The calculation method is that when raw materials or energy enter the first production sequence and the original form or property is changed or they are put into actual use, they are treated as consumption statistics.

**Inventory of Raw Materials and Energy** refers to the quantity of raw materials and energy actually stored in the beginning and end of the report period. The calculation principle of inventory is: “The one who disposes energy is responsible for conducting statistics on its inventory”, i.e., statistics is made according to the ownership. The calculation method refers to the quantity of raw materials and energy actually stored at a certain time that can be disposed by an enterprise.
