# Supplementary material for: The measurement of competitiveness of forest green food industry in Yunnan Province
Source: PLoS One. 2021 Dec 10;16(12):e0261133. doi: 10.1371/journal.pone.0261133 (PMC8664197; doi:10.1371/journal.pone.0261133)
Supplement: S1 File — http://stats.yn.gov.cn/. (Z01) [file pone.0261133.s001.z01 › the Yunnan statistical yearbook (2016-2020)/2016/9/九、建筑业和房地产业.doc]

主要统计指标解释

建筑施工企业 指从事房屋、构筑物和设备安装生产活动的独立施工单位。

建筑业总产值（自行完成施工产值） 指建筑施工企业在一定时期内所完成的以货币表现的生产总量。是反映全部生产规模、水平和成果的综合指标。

房屋建筑施工面积 指在报告期内施工的全部房屋建筑面积，包括本期新开工的房屋面积、上期施工跨入本期继续施工的房屋面积、上期停缓建在本期恢复施工的房屋面积、本期竣工的房屋面积及本期施工后又停缓建的房屋面积。

房屋建筑竣工面积 指在报告期内房屋建筑按照设计要求全部完工，达到了住人和使用条件，经验收鉴定合格，正式移交使用单位的房屋建筑面积。

自有机械设备年末总台数 指归本企业所有，属于本企业固定资产的生产性机械设备年末总台数。包括施工机械、生产设备、运输设备以及其他设备。

自有机械设备年末总功率 指本企业自有施工机械、生产设备、运输设备以及其他设备等列为在册固定资产的生产性机械设备年末总功率，按设定能力或查定能力计算。包括机械本身的动力和为该机械服务的单独动力设备，如电动机等。计算单位用千瓦，动力换算可按1马力=0.735千瓦折合成瓦数。电焊机、变压器、锅炉不计算动力。

营业收入 指企业经营主要业务和其他业务所确认的收入总额。包括主营业务收入与其他业务收入。

利润总额 指建筑施工企业在一定时期内实现的利润。

主营业务收入 指企业确认的销售商品、提供服务等主营业务收入。

建筑业增加值 指建筑企业在报告期内以货币表现的建筑生产经营活动的最终成果。

产值利润率 是指报告期内企业实现的利润总额占同期建筑业总产值的百分比。

房地产开发投资 指各种登记注册类型的房地产开发公司、商品房建设公司及其他房地产开发法人单位和附属于其他法人单位实际从事房地产开发或经营活动的单位统一开发的包括统代建、拆迁还建的住宅、厂房、仓库、饭店、宾馆、度假村、写字楼、办公楼等房屋建筑物和配套的服务设施，土地开发工程（如道路、给水、排水、供电、供热、通讯、平整场地等基础设施工程）的投资；不包括单纯的土地交易活动。

商品房销售面积 指报告期内出售商品房屋的合同总面积(即双方签署的正式买卖合同中所确定的建筑面积)。由现房销售建筑面积和期房销售建筑面积两部分组成。商品房销售额 指报告期内出售商品房屋的合同总价款(即双方签署的正式买卖合同中所确定的合同总价)。该指标与商品房销售面积同口径，由现房销售额和期房销售额两部分组成。

经济适用房 指根据经济适用房计划安排建设的政策性住宅。经济是指房屋建筑造价和销售价格低于一般商品住宅；适用是指适合中低收入家庭购买使用。经济适用房主要是由国家统一下达投资计划，房地产公司开发，对外销售；用地一般采用行政划拨或招标投标方式，免收土地出让金；对各种经批准的收费减半征收，开发利润不超过3%；销售价格实行政府指导价。该指标可以分析房地产投资结构，反映中低收入家庭商品住宅的供求平衡情况。

不可销售面积 指报告期房地产公司竣工的用于拆迁还建的房屋面积；接受委托、定向开发建设，并收取一定的管理费所建设的统建代建房屋竣工面积；竣工的学校、幼儿园、派出所、居委会、商店等公益设施建筑面积。

待售面积 指报告期末已竣工的可供销售或出租的商品房屋建筑面积中，尚未销售或出租的商品房屋建筑面积，包括以前年度竣工和本期竣工的房屋面积，但不包括报告期已竣工的拆迁还建、统建代建、公共配套建筑、房地产公司自用及周转房等不可销售或出租的房屋面积。按照商品房待售时间的长短可以划分为待售一年以下、待售一至三年（含一年）和待售三年以上（含三年）。

**Explanatory Notes on Principle Statistical Indicators**

**Statistical Entities in Construction** refers to corporate enterprises engaged in construction of buildings and structures and equipment installation.

**Gross Output Value of Construction** （**Output Value of Self-completed Projects**） refers to the total volume of construction products, expressed in monetary terms and completed by construction and installation enterprises during a given period of time. It is a comprehensive indicator reflecting the whole production scale, level and fruits.

**Floor Space of Buildings Under Construction** refers to the floor space of buildings under construction during the report period, including newly started buildings, buildings started in the preceding period and continued during the current period, and buildings suspended in the preceding period but restarted in the current period, buildings completed during the current period, and buildings under construction and then suspended during the current period.

**Floor Space of Buildings Completed** refers to the floor space of buildings that are completed in the report period in accordance with the requirements of the design, up to the standard of putting them into use, and have been checked and accepted by concerned departments as qualified ones.

**Total Number of Machinery and Equipment Owned at Year-end** refers to the number of machines and equipment owned by enterprises, and listed as their fixed assets by the end of the year, including machinery and equipment for construction, production and transportation.

**Total Power of Machinery and Equipment Owned at Year-end** refers to the total power of machinery and equipment for construction, production and transportation owned by enterprises, and listed as their fixed assets by the end of the year, which is calculated on the basis of the designed or verified capacity, covering the power of machinery and equipment and separate power equipment serving them （such as electric motors）, but excluding welders, transformers and boilers. The unit used for the calculation of power is kilowatt, with horsepower converted to kilowatt by 1 horsepower=0.735kilowatt.

**Operating Revenue** refers to the sum of income from principal business and other business of enterprises, including income from settlement of projects and other operating income.

Total Profits refer to the profits made by construction enterprises in a certain period of time.

**Income from Principal Business** refers to income received by merchandising and providing service of enterprises.

**Added Value of Construction** refers to the final results of production and operation of construction enterprises in monetary terms in the report period.

**Ratio of Profit to Output Value** refers to the ratio of the total profits to the gross output value of construction in the report period.

**Investment in Real Estate Development** refers to investment by real estate development companies, commercialized buildings construction companies and other real estate development units of various types of ownership in the construction of buildings, such as residential buildings, factory buildings, warehouses, hotels, guesthouses, holiday villages, office buildings, and the complementary service facilities and land development projects, such as roads, water supply, water drainage, power supply, heating supply, telecommunications, land leveling and other infrastructural projects. It does not include activities in pure land transactions.

**Area of Commercialized Housing Sold** refers to total contracted area of commercialized housing （i.e. area of floor space as designated in the formal contracts signed by both sides） during the reference time. It constitutes floor space of completed housing and floor space of future housing.

**Value of Commercialized Housing Sold** refers to the total contracted value （i.e. value of sales/purchase for selling/purchase of commercialized housing as designated in the contract signed by both sides） during the reference time. This indicator has the same coverage as the area of commercialized housing sold, which constitutes floor space of completed housing and floor space of housing yet to be completed.

Economically Affordable Housing refers to housing constructed according to the State Plan for economically affordable housing. The features of houses of this category are low cost of construction and low prices, and therefore are affordable to mid-income and low income households. Economically affordable housing projects are developed by real estate companies under the State Investment Plan, with the land provided through government allocation or tendering procedures. Developers are exempted from land utilization fees and enjoy another 50% exemption of all other legitimate fees, while their profits are limited to less than 3%, and the completed houses are sold under government-guided prices. This indicator helps to analyze the investment structure of the real estate industry and the demand and supply of housing for mid-income and low income households.

**Non-Marketable Area** refers to the building area used for removal and rebuilding which completed by real estate companies in reference period; completed building area of unified construction and acting construction which constructed by accepting commission, orientating development construction and charging certain management overhead; building area of completed schools, kindergartens, police stations, neighborhood committees, stores and such public utility constructions.

**Floor Space of Vacant Building** refers to the area of commercialized buildings haven't been sold or rent in the area of completed marketable and rentable commercialized buildings in the reference period, including the completed building area in the former years and in the current period, while excluding the completed removal and rebuilding buildings, unified and deputized construction buildings, public matched installations buildings and area of the buildings for self-use and revolving buildings of the real estate companies which are unmarketable or can't be rented. According to the vacant time, the buildings can be categorized as follows: buildings vacant less than one year, vacant for one to three years （including one year）, vacant more than three years （including three years）.
